# Ousiometrics: The essence of meaning aligns with a power-danger-structure framework instead of valence-arousal-dominance

**DOI:** 10.1126/sciadv.adr4039

**Published:** 2026-05-06

**Authors:** Peter Sheridan Dodds, Thayer Alshaabi, Mikaela Irene Fudolig, Julia Witte Zimmerman, Juniper Lovato, Shawn Beaulieu, Joshua R. Minot, Michael V. Arnold, Andrew J. Reagan, Christopher M. Danforth

**Affiliations:** ^1^ Computational Story Lab, Vermont Advanced Computing Center, University of Vermont, Burlington, VT 05405, USA.; ^2^ Vermont Complex Systems Institute, MassMutual Center of Excellence for Complex Systems and Data Science, University of Vermont, Burlington, VT 05405, USA.; ^3^ Department of Computer Science, University of Vermont, Burlington, VT 05405, USA.; ^4^ Santa Fe Institute, 1399 Hyde Park Rd, Santa Fe, NM 87501, USA.; ^5^ Complexity Science Hub, Metternichgasse 8, 1030 Vienna, Austria.; ^6^ Howard Hughes Medical Institute, Janelia Research Campus, Ashburn, VA 20147, USA.; ^7^ Advanced Bioimaging Center, University of California Berkeley, Berkeley, CA 94720, USA.; ^8^ School of Computer and Mathematical Sciences, University of Adelaide, Adelaide, SA 5005, Australia.; ^9^ Computational Ethics Lab, University of Vermont, Burlington, VT 05405, USA.; ^10^ MassMutual Data Science, Amherst, MA 01002, USA.; ^11^ Department of Mathematics & Statistics, University of Vermont, Burlington, VT 05405, USA.

## Abstract

From work emerging through the middle of the 20th century, the essence of meaning has become widely accepted as being described by the three orthogonal dimensions of valence, arousal, and dominance. These essential dimensions have become the cornerstone of sentiment analysis across many fields. By reexamining first types and then tokens for the English language, and through the use of automatically annotated histograms—“ousiograms”—we find here that the essence of meaning conveyed by words is instead best described by a goodness-power-aggression-danger-structure (GPADS) circumplex framework; that large-scale English language corpora reveal a systematic bias toward safe, low-danger words; and that the power-danger-structure framework is the minimal framework that represents essential meaning. We find remarkable congruences between the GPADS framework and other spaces including mental states and fictional archetypes, and we construct and demonstrate a prototype ousiometer.

## Teaser

The three orthogonal axes of essential meaning are weak-powerful, safe-dangerous, and structured-unstructured.

## INTRODUCTION

As encoded by human language, meaning spans a high-dimensional semantic space that is continually expanding and evolving, bearing complex hierarchical and networked structures (*1*–*3*). In attempting to understand any quantified complex system, a most basic step is to apply a method of dimensional reduction. If we distill meaning to its essence, boiling off all higher dimensions of meaning—the focus of our work here—do we find fundamental dimensions of meaning space that are interpretable and, moreover, reliably experienced, conceptualized, and conveyed by people (*4*–*10*)?

The short answer is yes—Osgood *et al.*’s methods (*6*) (see below) have proved to be successful and the Osgood framework has come to be widely used across many fields, but as we will show, Osgood’s framework does not fully hold up to a modern computational analysis using large-scale datasets and new methodologies. Here, we define “ousiometrics” to be the quantitative study of the essential meaningful components of an entity, however represented and perceived. Used in philosophical and theological settings, the word “ousia” comes from Ancient Greek $o\overset{,}{\upsilon }$σία and is the etymological root of the word “essence” whose more modern usage is our intended reference.

Introducing the terminology of ousiometrics in particular helps us distinguish from general fields that study meaning such as semantics or semiotics. For framing purposes, being able to thematically name specialized tools and instruments will be helpful in the presentation of our work. In particular, we will develop and explore a series of “ousiograms” that are annotated representations of two-dimensional slices of meaning space. For our purposes here, and in the tradition of Osgood *et al.* (*5*, *6*) and the many who have followed (*9*–*19*), our measurement of essential meaning will rest on—and is constrained by—the map presented by language.

### How the measurement of essential meaning has been operationalized

To help explain the purpose of our paper—why our approach to ousiometrics is warranted—we outline the relevant history of measuring essential meaning, and describe the long-standing problematic aspects of experiment design and measurement instruments. The quantitative measurement of the essence of meaning was primarily developed by researchers in the middle of the 1900s, particularly by Osgood and colleagues, with their foundational work published in the 1950s (*5*, *6*). The majority of ensuing research (*7*, *8*, *13*) has been built around human evaluation of words or phrases using the instrument of the semantic differential (*5*, *6*, *20*). In a typical study, surveyed participants are asked to rate individual words on Likert scales with end points described by “bipolar adjectival pairs” such as {soft ⇔ hard}, {rough ⇔ smooth}, and {cold ⇔ hot}. Throughout, we will indicate semantic differentials as per the examples of the preceding sentence, bracketing and connecting bipolar adjectival pairs with the symbol ⇔. The measurement of essential meaning is thus operationalized via surveys that ask people to respond to isolated words and phrases, given some semantic differential and some scale.

Modern support for semantic differentials comes from findings that large language models (LLMs) encode semantic axes consistent with human judgment. Grand *et al.* (*21*) demonstrate that “semantic projection”—mapping embeddings onto differential axes like size, age, and wealth—recovers feature-level conceptual knowledge. 

Each semantic differential is considered a dimension (an axis) in a potentially high-dimensional space, and researchers then apply some variant of factor analysis to the average scores, such as principal components analysis (PCA) or singular value decomposition (SVD), or more sophisticated methods (*22*–*26*). In general, factor analysis determines a set of dimensions that are linear combinations of the study’s semantic differentials, which must then be interpreted. As we detail below, we are able to use basic SVD, and it is in the interpretation step that we develop a new systematic methodology.

On the basis of a range of studies, Osgood *et al.* (*6*) identified three orthogonal dimensions for the essence of meaning. In order of variance explained for the studies at the time, the three dimensions were dubbed:

1) Evaluation (e.g., {positive ⇔ negative}),

2) Potency (e.g., {dominant ⇔ submissive}), and

3) Activity (e.g., {active ⇔ passive}).

Although the “EPA” (evaluation-potency-activation) framework has been challenged in various ways (*9*, *27*, *28*), as have semantic differentials themselves (*9*, *19*), researchers were increasingly drawn to take the EPA framework as a ground truth when carrying out new studies (*13*, *15*, *19*). In the focused context of studying emotion, a theoretical concept of a three-dimensional representation of emotion goes back to (at least) Wundt in the late 1800s (*29*, *30*). For emotion, the EPA dimensions were re-ordered and recast by Mehrabian and Russell as: (i) pleasure or valence, (ii) arousal, and (iii) dominance (PAD or VAD) (*11*, *12*). To make clear that this was the authors’ intention, from the summary of (*12*):

“Semantic differential studies, in particular, have shown that human judgments of diverse samples of stimuli can be characterized in terms of three dimensions: evaluation, activity, and potency. We have termed the corresponding emotional responses pleasure, arousal, and dominance.”

Subsequent work has tended to use the term valence instead of pleasure, and we will follow the VAD nomenclature. The VAD framework has seen extensive application across diverse disciplines. In psychology (*15*, *31*), neuroscience (*32*, *33*), and natural language processing (NLP) (*34*, *35*), VAD provides dimensional models to capture affective meaning. Other areas include sentiment analysis (*36*, *37*), emotion detection (*38*, *39*), marketing (*11*, *40*), human-computer interaction (*36*, *41*)(*41*), and education (*42*, *43*).

Now, while VAD was intended to be a scoped version of EPA, the two frameworks have been conflated. Generally, VAD has become the framework presented in studies, even when essential meaning, rather than emotion, has been the focus (*13*, *15*, *19*). Elsewhere, the original connection between VAD and EPA has been overlooked or considered broken, leading to reanalyses about whether or not the match between EPA and VAD holds at all (*44*). Nevertheless, to be consistent with the direction taken by the literature, we will refer to VAD rather than the more general EPA going forward.

### The major problems with measuring essential meaning

We describe a set of problems that we contend have thwarted the full development of ousiometry over time.

#### Scale

Given that the EPA framework was developed before and during the 1950s, the foundational studies were limited in size, in both lexicon analyzed and the number of participants surveyed. For example, as part of the research that led to the EPA framework, Osgood *et al.* (*6*) report on a study of 20 concept nouns evaluated on 50 semantic differentials by 100 undergraduates. Published in 1980, Russell’s circumplex model of affect (which we examine later in the “Congruences: Russell’s circumplex model of emotion” section) was based on the scoring of 28 words and phrases (*9*). The Affective Norms for English Words (ANEW) study of the late 1990s moved the lexicon size up to 1034, but with VAD as the accepted fundamental framework, and still using surveys of undergraduates. In work carried out around 2010 involving two of the present authors, an order of magnitude jump to more than 10,000 English words was conducted online through Amazon’s Mechanical Turk with 50 evaluations per word along the single semantic differential of valence interpreted as happiness (discussed further below) (*14*). This dataset, labMT (language assessment by Mechanical Turk), was later expanded to 10 languages, each with more than 10,000 words scored online by participants around the world (*45*). Crucially, and in contrast to the ANEW word lists, the labMT words analyzed were chosen according to frequency of usage (again, discussed further below). In 2013, Warriner *et al.* (*15*) published scores for close to 14,000 English words with VAD scores. Last, in 2018, Mohammad (*19*) produced what will be the basis of our analysis here, the NRC VAD lexicon: more than 20,000 English words and phrases with VAD scores.

Thus, it is only in the last 15 years that studied lexicons have begun to represent the scale of human vocabularies. We are consequently now well placed to perform the necessary work of re-examining the findings of the field’s foundational research.

#### The focus on types alone and not tokens

We use the standard type-token language for describing entities (*46*): Type refers to an entity’s class (or species) while token refers to an entity itself as an instance of that class. Beyond language, the type-token distinction appears across all complex systems with heavy-tailed distributions of component frequencies. Perhaps in settings not involving words and texts, the problems with studying only types would be more apparent. For example, in determining some overall measure of a forest, we would not want to assign equal weight to the most common and the most rare species. Here, we will study both lexicons (types) and large-scale texts (tokens), gaining separate results from both.

Almost all essential meaning studies have been at the level of types, each word or concept given equal weighting. However, we must consider the weight of types in a text according to the frequency of their corresponding tokens (*46*). Only then can we make defensible observations about a whole space of communication. The ANEW study (*13*), for example, is based on 1034 expert chosen words, which proved to be a poor fit for natural language (*47*). By contrast, with careful consideration of word usage, we were able to show that the Pollyanna principle (*48*) manifests a linguistic positivity bias across 24 corpora spanning 10 languages (*45*).

#### The use of Likert scales for semantic differentials

The use of a Likert scale for evaluations of semantic differentials has long been standard practice. Relatively recently, best-worst scaling has been suggested to be a more robust instrument than the Likert scale, as well as a far more efficient one (*49*). To our great benefit, Mohammad’s survey of more than 20,000 words and phrases preferentially uses best-worst scaling, finding appreciable improvement in split-half reliabilities over studies using Likert scales.

#### Limitations of factor analysis for a large number of categorical dimensions

While tables of factor analysis weightings can be exhaustively informative for small-scale studies, we will not be able to make much sense of point clouds of tens of thousands of unlabeled words in two or three dimensions. Here, we will show how a kind of automatically annotated histogram—an ousiogram—coupled with ranked word lists provides an instrument that will help us explore, describe, and support our assessments of the dimensions of essential meaning.

#### The misalignment between expert-chosen, end-point descriptors and dimensions of essential meaning

We come to a critical problem with any essential meaning study that starts from a presumption of the EPA/VAD framework. We go back to basics and outline the four-step experimental process that has been used to extract essential dimensions of meaning in the first place:

1) Participants are asked to rate a set of $N_{\text{types}}$ types (e.g., words and images) using a set of $N_{\text{differentials}}$ semantic differentials defined by bipolar adjectival pairs. Some examples from Osgood *et al.*’s [p. 43 in (*6*)] 50 semantic differentials for the study mentioned above include {large ⇔ small}, {clean ⇔ dirty}, {brave ⇔ cowardly}, {bass ⇔ treble}, and {near ⇔ far}.

2) Some variant of factor analysis (e.g., PCA and SVD) is then used to obtain an ordered set of dimensions that are linear combinations of the semantic differential dimensions.

3) Researchers interpret the main dimensions and ascribe them with both descriptive names (e.g., “evaluation”) and, crucially, sets of “end-point descriptors” (e.g., happiness, pleasure, and contentedness for high valence and unhappiness, annoyance, and negativeness for low valence). These new semantic differentials are not then described by simple bipolar adjectival pairs but rather clusters of words and phrases at each end.

4) Researchers reduce the meaning space to two or three of the most prominent dimensions (e.g., by variance explained through singular values).

With ousiometric dimensions so determined (e.g., EPA), researchers then move on to new studies using only a modified version of step 1:

1) Participants are asked to rate a set of *N*_types_ types along two or three expert-chosen dimensions that are defined by expert-identified sets of end-point descriptors.

As such, there is then no assurance that the expert-identified end-point descriptors will be construed by participants in a way that imposes the expert-defined dimensions. We observe that across many studies, raters have been presented with end-point descriptors that render the three VAD dimensions with problematic imprecision (*6*, *9*, *13*, *19*, *44*). For example, for the ANEW study, valence was described to participants as a {happy ⇔ unhappy} scale as follows (emphasis added):

“At one extreme of [this {happy ⇔ unhappy}] scale, you are happy, pleased, satisfied, contented, hopeful. … The other end of the scale is when you feel completely unhappy, annoyed, unsatisfied, melancholic, despaired, or bored.”

The meaning captured by both ends is broad, the numbers of descriptors differ, and the word “bored” clearly overlaps with the dimension of arousal. For the NRC VAD lexicon, raters were guided by end-point descriptors (“paradigm terms”) that were taken from (*6*, *9*, *13*). As for the ANEW study, the end points for each dimension combine to create coarse semantic limits. We list all descriptors for the six end-points used in (*19*) in table S1. For example, for low arousal, there is clear semantic separation between “sluggishness” and “calmness,” as there is for “weak” and “guided” for low dominance.

Our remedy is simple: Always carry out steps 1 to 4 above even when attempting to impose a minimal ousiometric framework. Factor analysis will then accommodate a reasonable lack of exactness in how dimensions are prescribed. If we find that the VAD framework is in fact perfectly prescribable, we will have done the work needed to make this clear.

#### Presuming that the VAD framework does capture essential meaning and that the three dimensions are orthogonal

As we have observed, Osgood *et al.*’s (*6*) EPA/VAD framework has become generally accepted as valid. However, modern, large-scale VAD evaluations of words and phrases have increasingly pointed toward the VAD framework being nonorthogonal. Leaving aside problematic sampling of words, the ANEW study (*13*) found evidence that arousal was mildly positively correlated with the magnitude of valence. The near 14,000 lemma VAD study of Warriner *et al.* (*15*) found correlations between the three VAD dimensions, the strongest being between valence and dominance with $r_{\text{Va},\text{Dm}}≃0.72$ (Pearson’s correlation), which prompted the authors to call into question the orthogonality of the VAD framework.

Most recently, using best-worst scaling for the NRC VAD lexicon, Mohammad (*19*) found a somewhat weaker correlation of $r_{\text{Va},\text{Dm}}≃0.49$, and then asserted that valence and dominance were only “slightly correlated,” a view with which we do not agree. In reference to the valence-dominance correlation in the Warriner *et al.* study (*15*), Mohammad stated:

“Given that the split-half reliability score for their dominance annotations is only 0.77, the high V-D correlations raises the suspicion whether annotators sufficiently understood the difference between dominance and valence.”

Thus, the suggestion here is that the problem is not that the VAD framework is not orthogonal, but that participants failed to grasp the definitions of dimensions. Our position, per problem 5 above, is that imposing VAD dimensions experimentally through end-point descriptors is a difficult task and that factor analysis is always required, and in challenging the VAD framework, we will show that these observed correlations are real and understandable, and ultimately lead to a revised framework we will identify to be power-danger-structure (PDS). We note that we do not use the expanded NRC VAD lexicon described in (*37*) as the lexicon merges words scored with best-worst scaling and traditional Likert scale.

### Roadmap for the paper

We first describe the datasets we analyze and explore. We make the key distinction between text corpora that are type based (i.e., lexicons) or token based (written or recorded expression) (*46*).

Through a series of integrated figures, we then demonstrate our four main findings: (i) The framework of valence-arousal-dominance (VAD) is far from being an orthogonal system (*15*), and this failure is due to the difficulties of constructing semantic differentials for essential dimensions of meaning (“Ousiograms” and “The VAD framework is not orthogonal” sections). (ii) A goodness-aggression-structure (GAS) framework and a PDS framework both provide two alternative, interpretable, and interconnected orthogonal systems that agree with earlier circumplex formulations, and which we will combine as a goodness-power-aggression-danger-structure (GPADS) framework (“Assessing the failure of the VAD framework”, “The GAS framework”, “The PDS framework”, and “A cube model of meaning for the GPADS framework” sections). (iii) Only the PDS framework aligns with the essential meaning patterns of real corpora when we properly account for frequency of usage by considering tokens. (iv) Diverse, large-scale text corpora present a systematic, low-danger “safety bias” (the “The linguistic ‘safety bias’ of disparate large-scale corpora” section).

With the GAS, PDS, and GPADS frameworks established, we identify remarkable congruences in two other areas that are far removed from how people respond to isolated words: (i) the influential circumplex model of affect (*9*) (the “Congruences: Russell’s circumplex model of emotion” section); and (ii) archetypometrics—the data-driven determination of fictional character archetypes (*50*) (the “Congruences: Fictional characters” section). Last, we briefly summarize our results and offer thoughts on future work.

## ANALYSIS, RESULTS, AND DISCUSSION

### Description of datasets

We build our findings in two stages using two distinct kinds of word lists: (i) types: A lexicon for the English language (each word is of equal importance), and (ii) tokens: Frequency-rank distributions (*51*) for large-scale corpora (words are ranked by frequency of usage with the most used word having rank 1; words with tied frequency are given an average of corresponding ranks if they were not tied). In general, observations made solely by examining a lexicon (the level of types) will be given a stringent test when confronted by real-world word usage (the level of tokens).

The type stage: As indicated in the Introduction, we use the NRC VAD lexicon comprising around 20,000 words and terms (*19*). The lexicon was compiled from a variety of sources and largely contains lowercase, Latin-character words along with some 2-, 3-, and 4-grams (an *n*-gram is a phrase made up of *n*-terms). Proper nouns and function words have generally been excluded. Some words are evidently hashtag constructions from social media (with the hashtag removed). The lexicon is a union of existing lexicons, some of which were expert-compiled [e.g., the ANEW study (*13*)] and others based on frequency of usage. While the presence of expert-compiled lexicons is not ideal, we will see that the coverage of real corpora is sufficient for the purposes of our work here.

For each term in the NRC VAD lexicon, scores within the VAD framework (*11*, *12*) were assessed by survey using best-worst scaling (*49*). Terms were presented in groups of four and participants were asked to rank the highest and lowest according to one of the three VAD dimensions [see (*19*) for full details]. Each term’s score is in the interval [0,1]. To accommodate SVD, we remove the mean from each dimension, which by the nature of best-worst scaling is $\frac{1}{2}$. We thus shift the VAD scores from [0,1] to [$-\frac{1}{2}$,$+\frac{1}{2}$].

The token stage: With findings from studying the NRC VAD lexicon, we then analyze seven corpora—where frequency of word usage now matters—which vary broadly in kind, formality, scale, and historical time frame.

1) English Fiction (1900 to 2019) from the Google Books project, with each book contributing words equally, and then each year’s size-rank distribution weighted equally (*52*, *53*);

2) Jane Austen’s six novels with all books merged, sourced from the Gutenberg Project, http://gutenberg.org;

3) The majority of Arthur Conan Doyle’s Sherlock Holmes stories with all stories merged (four novels and 44 short stories taken from https://sherlock-holm.es/, missing the 12 short stories contained in *The Case-Book of Sherlock Holmes*);

4) *The New York Times* (1987 to 2007) frequency-rank distributions merged without reweighting across all years (*54*);

5) Wikipedia (English language, March 2019 snapshot) (*55*);

6) RadioTalk (transcriptions of talk radio broadcasts in the United States, October 2018 to March 2019) (*56*); and

7) Twitter (approximately 10% of all tweets identified as English in 2020—including retweets—with each day’s frequency-rank distribution contributing equally) (*57*).

### Ousiograms

Complex systems are often manifested from a set of distinct, named entities—types—whose frequencies of occurrence as interacting tokens roughly obey a heavy-tailed distribution, and whose characteristics reside in some high-dimensional space (*51*, *58*–*61*). Language is a canonical example with words as types and meanings as one of their characteristics. One approach to better understanding such high-dimensional complex systems is thorough dimensional reduction where we maintain the set of all types but seek to distill the characteristics of these types down to an essential few.

To inform and help validate our analysis, we will use ousiograms. We define an ousiogram as a systematically and informatively annotated two-dimensional histogram for two essential quantities of a complex system’s component entities. The entities represented in ousiograms may be either types or tokens (*46*), with types contributing equally while a token’s contribution would be proportional to the frequency of that token’s appearance within a given system.

**Fig. 1.**
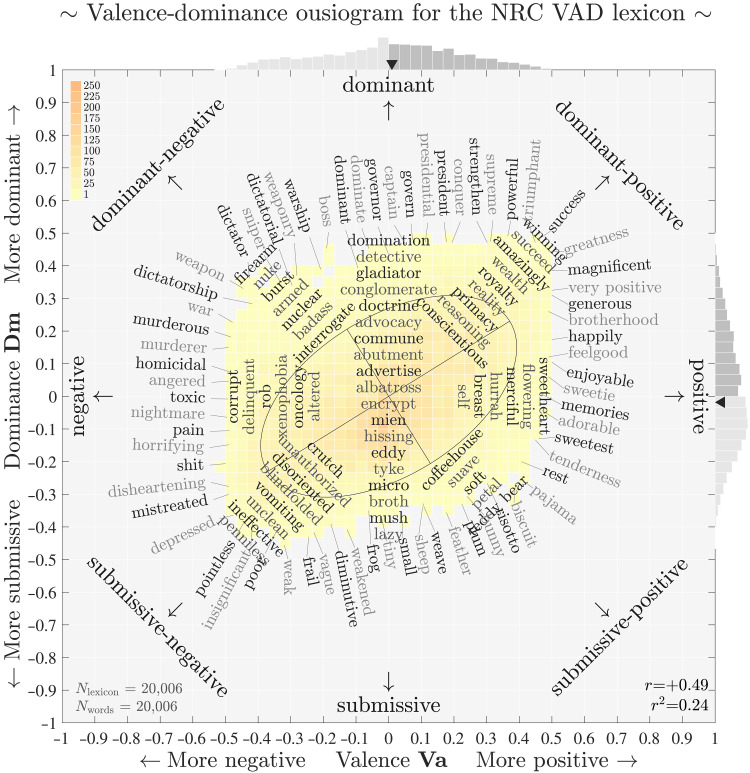
Valence-dominance ousiogram for the NRC VAD lexicon of around 20,000 words scored within the valence-arousal-dominance (VAD) framework using best-worst scaling. Ousiograms are annotated two-dimensional histograms of two essential dimensions describing any collection of labeled entities. Here, we arrange words according to their valence-dominance scores, collapsing the third dimension of arousal. We use a bin width of 1/30, and we have shifted all **Va**, **Ar**, and **Dm** scores from [0,1] to [$-\frac{1}{2}$,$+\frac{1}{2}$]. To enable comparisons, we use limits of [−1,1] throughout the paper. We plot marginal distributions of **Va** and **Dm** along the top and right sides, with darker gray indicating positive values, and solid dark triangles locating the medians of **Va** and **Dm**. The ellipse represents the axes determined by SVD acting on the **Va-Dm** plane, and shows a strong departure from the **Va** and **Dm** axes. We label words around the edge of the Va**-**Dm distribution aligned with normals to the distribution’s convex hull, and add example words at internal locations along the main axes and the two diagonals. Upon inspection, the words shown are reasonably located according to their essential values of **Va** and **Dm**. See figs. S1 and S3 for large-scale ousiograms of **Va-Ar** and **Ar-Dm**. Labeled words are not restricted in their value of the third dimension, arousal **Ar**, which may vary unevenly. Alternating shades of gray are for readability. For these larger ousiograms, we automatically label the four cardinal and intercardinal directions with their end-point adjective (e.g., “dominant-positive” in the northeast corner).

**Fig. 2.**
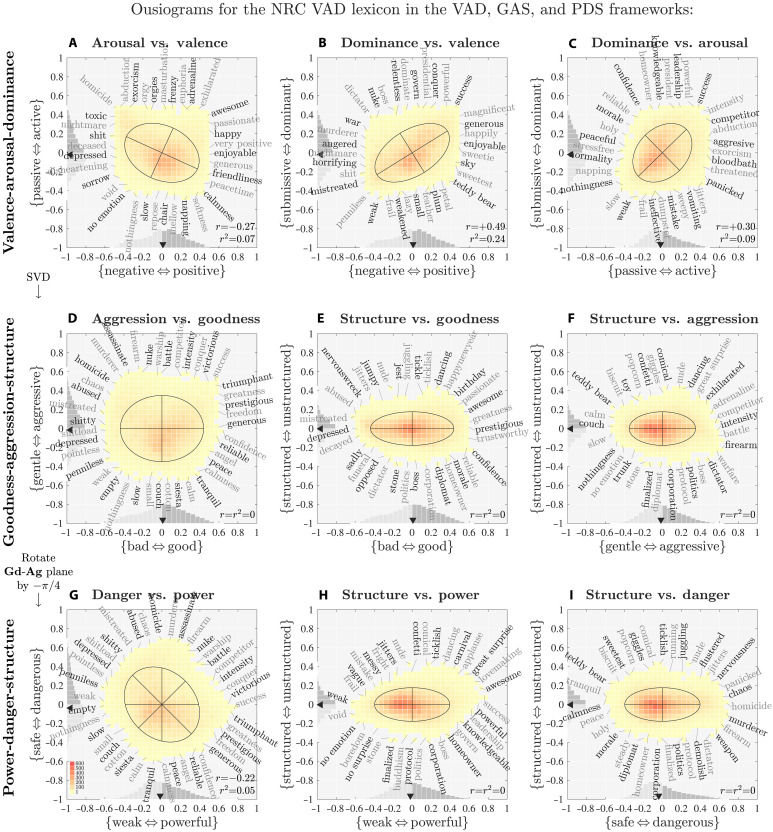
Ousiograms showing the analytic sequence moving from the valence-arousal-dominance (VAD) framework (top row) to the goodness-aggression-structure (GAS) and power-danger-structure (PDS) frameworks (second and third rows). Row 1 (**A** to **C**): Ousiograms for the three pairs of variables **Va**, **Ar**, and **Dm** for ~20,000 words in the VAD NRC lexicon (*19*) [(B) corresponds to Fig. 1]. We determine the ellipses by using singular value decomposition (SVD) in each plane, ignoring the third dimension. The ill fit of the VAD framework is apparent for the misalignments of ellipse axes. Word annotations along the edges of the nine pairwise distributions, coupled with ranked words lists by component size (figs. S29 to S33), enable interpretation of the new frameworks of GAS and PDS. Row 2 (**D** to **F**): We perform SVD on the full matrix formed by the **Va**, **Ar**, and **Dm** scores, and identify goodness **Gd**, aggression **Ag**, and structure **St**, with the first two dimensions accounting for more than 90% of explained variance. Row 3 (**G** to **I**): Rotating the goodness-aggression plane by −π/4, we uncover a framework with {weak ⇔ powerful} and {safe ⇔ dangerous}. The GA and PD dimensions interlink to form an interpretable circumplex model GPADS. See Fig. 3 for a larger, more detailed power-danger ousiogram. As any lexicon reflects only the possible but not the used language (types versus tokens), whether or not the VAD, GAS, or PDS frameworks are sensible must be tested by considering real corpora. See the “Assessing the failure of the VAD framework” section and Eqs. 2 and 4 for interpretation of the VAD, GAS, and PDS relationships.

In Fig. 1, we present an ousiogram for valence **Va** and dominance **Dm** for the NRC VAD lexicon (*19*). We use valence and dominance as an example to demonstrate the nonorthogonality of the VAD framework with best-worst scaling. In figs. S1 to S9, we provide the corresponding **Va**-**Ar** and **Ar**-**Dm** large-scale ousiograms. For our main analysis, we present smaller versions of these ousiograms in Fig. 2 (A to C), which we discuss below in the “Assessing the failure of the VAD framework” section.

We first briefly describe the ousiogram in Fig. 1 (see the figure’s caption for more detail), and then contend with the nonorthogonality of the VAD framework. As a guide, we label the cardinal directions for valence **Va** and dominance Dm by the standard (if problematic) bipolar adjectival pairs anchoring the semantic differentials {negative ⇔ positive} and {submissive ⇔ dominant}. The intercardinal directions are then combinations of these adjectival pairs (e.g., submissive-positive). We label all other ousiograms in the same fashion with appropriate bipolar adjectival pairs.

Given that we have shifted the VAD scores to lie in [$-\frac{1}{2}$,$+\frac{1}{2}$], the two-dimensional histogram of Fig. 1 shows that the NRC VAD lexicon accesses much of the available **Va**-**Dm** plane. The marginal distributions at the top and right show that both valence and dominance are well dispersed, with dominance exhibiting some minor asymmetry. The dark triangles indicate the medians for each marginal.

We show words using two kinds of annotations: At the extremes of the histogram’s boundary and internally along the cardinal and intercardinal axes [see (*15*) for scatter plots with perimeter annotations]. For words on the boundary, we automatically construct and segment a convex hull for the histogram, determine normals to each segment, and annotate the closest word. Internally, we find words closest to points along the eight outgoing lines. We leave the third dimension aside (here, arousal **Ar**). Both the bin width for the underlying histogram and the spacing of annotations are tunable, and we avoid annotating a word more than once.

Ousiograms will have two main benefits for us. First, they give us a way to check that words line up with prescribed axes. Second, and crucially for our later work here, when we move to a potential new framework, ousiograms will help us to interpret the underlying axes.

In the first sense of acting as a check, the ousiogram of Fig. 1 shows that word ratings performed by the survey participants in (*19*) are reasonably sensible. Traveling around the histogram’s boundary, we see how the essential meaning of words incrementally changes. Starting in the “dominant-positive” direction (upper right), we see “triumphant,” “success,” and “greatness.” As we move clockwise going down the right side of the boundary, the annotated words become softer while remaining pleasant: “generous” to “memories” to “pajama.” Moving left along the bottom boundary, positive gives way to negative, and we reach the extreme of negative-submissive: “feather” to “weakened” to “pointless.” Moving up the left side, we see a string of negative words that grow in strength, partly because of the scope of dominance: “depressed,” “nightmare,” “murderous,” and “dictator.” Returning across the top of the ousiogram, we move through martial, leadership, and power terms that gradually lessen in violence: “weaponry,” “dominate,” “president,” “powerful,” and back to “success.”

Internally, each of the eight directions leading out from the center also reflect changes in the strength of essential meaning. For the full negative-submissive to dominant-positive axis, for example, we track from “penniless,” “vomiting,” “disoriented,” and “crutch,” up through to “conscientious,” “qualifying,” “amazingly,” and “success.”

The words “encrypt” and “albatross” are neutral in the Va-Dm plane and are worth reflecting on. These are certainly meaningful words, and as for all words, these examples could take on a strong meaning in the right context. An albatross for sailors is a dire omen whereas an albatross in golf is a rare, extraordinary success. However, raters are asked to compare the essential meaning of words based on the perceived meaning in isolation, which is to say, in the context of the rater’s knowledge of the word.

### The VAD framework is not orthogonal

We turn now to the issue of orthogonality, a long-standing point of contention for the EPA and VAD frameworks (*6*, *9*, *11*, *12*, *15*, *19*). For the NRC VAD lexicon, we find that the VAD dimensions as interpreted by raters are not close to being orthogonal. We observe that standard correlation coefficients for the three pairs of VAD variables are

$$r_{\text{Va},\text{Ar}}≃-0.27,\;r_{\text{Ar},\text{Dm}}≃0.30,\;\text{and}\;r_{\text{Va},\text{Dm}}≃0.49$$ (1)

where the corresponding *P* values are computed to be essentially 0. If the VAD framework were orthogonal, these three correlation coefficients should be statistically indistinguishable from 0.

We note that the linkages between the VAD dimensions are not simple, with valence and arousal being anticorrelated with the other two pairs being positively correlated. For a visual guide, and one that we will use throughout the paper, the ellipse in Fig. 1 represents the coordinate system uncovered by SVD (*62*) in the **Va**-**Dm** plane (we ignore **Ar** for this example calculation). The ellipse is clearly off axis. For the equivalent ellipses for the **Va**-**Ar** and **Ar**-**Dm** planes, see the ousiograms in Fig. 2 (A and C) as well as in the Supplementary Materials.

Now, given that we do not see orthogonality for the VAD framework for the largest lexicon ever studied coupled with a markedly improved rating system, we are compelled to investigate why VAD (equivalently EPA) fails as an orthogonal framework and what alternate framework might be revealed in doing so.

The root cause of confusion lies in the difficulty of ascribing stable and meaningful end-point descriptors for VAD (and EPA) variables. As was true for Osgood *et al.*’s work (*6*) that led to the EPA framework, from the start in developing the VAD framework (*11*, *12*), Mehrabian and Russell were concerned with both orthogonality and finding suitable end-point descriptors. As explored in (*44*), researchers have continued to use a varying array of end-point descriptors for EPA and VAD, including the same researchers over time (*9*, *13*, *19*).

Problematically, and as we noted in the Introduction, some end-point descriptors have the effect of correlating different dimensions. For example, in the ANEW study in (*13*), the negative valence end-point was presented to participants as a state of feeling “completely unhappy, annoyed, unsatisfied, melancholic, despaired, or bored.” The last descriptor “bored” evidently would be elicited at the low end point of the arousal dimension, which itself was framed as “completely relaxed, calm, sluggish, dull, sleepy, or unaroused.”

For the NRC VAD lexicon we study here (*19*), the end points were described by six or seven words or phrases, unavoidably broadening them away from being sharply defined (table S1). For example, the words “happiness” and “hopefulness” are used for high valence, “unhappiness” and “despair” for low valence, “activeness” and “frenzy” for high arousal, and “relaxation” and “sluggishness” for low arousal (see table S1 for all descriptors). There is a gap in meaning between all of these pairs of words, and how participants might perform at rating or ranking words is not a priori clear.

A further complication is that the names of the dimensions themselves do not track well within the VAD framework. While not strictly necessary that they do so, if the name of dimension is a word with a common meaning, then raters may be guided away from an intended direction in meaning space. For example, the word “arousal” is itself high on arousal (**Ar** = 0.44) but also registers on the valence and dominance dimensions **Va** = 0.29, **Dm** = 0.23, and while the word “dominance” scores strongly in dominance and neutrally for valence, it does pick up in the arousal dimension with (**Va**, **Ar**, **Dm**) = (0.04, 0.28, 0.34). By contrast, “valence” is sufficiently rare—it is not part of the NRC VAD lexicon—that it does not color how it is defined for the measurement of emotion. We are of course not suggesting that there is a simple solution to such ousiometric nomenclature issues—we are after all using words to define words as well as kinds of meanings of words.

While we have critiqued how end-point descriptors have been used, we are not saying such an approach is invalid. Rather, we point out that (i) the EPA dimensions were originally outputs of relatively small studies involving numerous semantic differentials, and (ii) the attempt to then make these dimensions controlled inputs to new studies is an entirely different exercise. In sum, the NRC VAD lexicon, the output of Mohammad’s study (*19*), does not align with the VAD framework, even though the VAD framework was the intended input.

To move forward, we observe that for any essence-of-meaning study, if participants are guided by some well-constructed set of end-point descriptors, then we can always compare and reconsider how well these descriptors perform. Moreover, we must allow that a distinct framework may emerge over time as far larger and more sophisticated studies are carried out. We are effectively maintaining the approach of the founding experiments, allowing the outcomes to remain informative and be potentially corrective.

### Assessing the failure of the VAD framework

In the present and following two sections, we show how the NRC VAD lexicon affords two possible alternate and mutually consistent frameworks: GAS and PDS. The steps of our analysis are represented by the rows of Fig. 2, which we explain as follows.

Throughout these sections, we complement our analysis with four pieces in the Supplementary Materials: Large-scale ousiograms in figs. S1 to S9; explorations of synousionyms and antousionyms, words that are similar or opposite in terms of essential meaning; an “MRI” of meaning space rendered as a flipbook in figs. S10 to S28; and lists of the top 20 words ranked by component size along the 13 axes of a 3 × 3 cube that is aligned with the SVD dimensions in figs. S29 to S41.

We first note that for the NRC VAD lexicon, the overall contributions to variance explained by the three VAD dimensions of meaning are approximately 44.4, 28.0, and 27.6%. Valence is clearly the leading dimension with arousal and dominance balanced.

To determine the uncorrelated orthogonal dimensions for the NRC VAD lexicon, we perform SVD on the three by 20,006 matrix A of average VAD scores ($A=U\Sigma V^{T}$). We find singular values $\sigma_{1}≃34.1$, $\sigma_{2}≃27.2$, and $\sigma_{3}≃13.8$, which correspond to explained variances of 55.6, 35.3, and 9.1. The first two dimensions now explain 90.9% as opposed to 72.4% explained by Va and Ar.

The point cloud of VAD scores is thus a nonaxis-aligned ellipsoid, strongly flattened in one dimension. In Fig. 2, the first row of ousiograms shows projected histograms of the ellipsoid in VAD space for each pair of dimensions (Fig. 2B corresponds to Fig. 1). The SVD ellipses in all three projections demonstrate the correlations in Eq. 1.

As for all ousiograms, the word annotations help us understand how raters have responded to the end-point descriptors. Here, these annotations may be diagnostic (VAD) or illuminating (GAS and PDS, below). For VAD, we have already considered Va-Dm ousiogram’s annotation (Fig. 2B), finding them to be sensible, and we see that annotations for the other dimension pairs are similarly interpretable within the VAD framework (Fig. 2, A and C).

### The GAS framework

Moving to the middle row of panels (Fig. 2, D to F), we show ousiograms for word scores represented by the orthogonal basis determined by SVD acting on the VAD word scores. By construction, all three SVD ellipses are now aligned with the underlying axes.

Upon considering the annotated words, along with the ranked word lists in figs. S30, S32, and S33, we interpret these three new essence-of-meaning dimensions to be goodness **Gd**, aggression **Ag**, and structure **St**. (For annotations internal to each histogram, see the larger ousiograms in Supplementary Materials.)

To arrive at the goodness dimension, we look to words on the left and right side of the ousiogram in Fig. 2D. On the left, we see “shitty,” “penniless,” “mistreated,” and “abused”; on the right, “reliable,” “confidence,” “freedom,” and “triumphant.”

Words at the bottom and top of the same ousiogram in Fig. 2D are connected in essential meaning by their signifying of low and high aggression: “slow,” “couch,” “siesta,” and “calm,” versus “assassinate,” “battle,” “competitor,” and “conquer.”

Last, we distill the vertical dimension in the ousiograms of Fig. 2 (E and F) as structure. We choose the alignment of the third dimension to be {structured ⇔ unstructured}, moving upward. At the bottom of these ousiograms, we have words connoting organization, rigidity, and systematic form: “stone,” “protocol,” “corporation,” “dictator,” and “diplomat.” At the top, we see terms that convey lack of structure: “jest,” “confetti,” “dancing,” “popcorn,” and “great surprise.” To support the choice of orientation for the structure axis, we make a thermodynamic analogy where rigid organization is akin to a zero temperature frozen state, and a growing lack of structure corresponds to increasing temperature. We also see that {serious ⇔ playful} and {predictable ⇔ unpredictable} differentials are subsets of the more general {structured ⇔ unstructured} differential. Broadly speaking, we view the third dimension of essential meaning {structured ⇔ unstructured} as encoding evolvability.

For purposes of clarity of argument, we have sought to choose valid but distinct names for the three dimensions in GAS to distinguish them from VAD (or EPA). We acknowledge that valence, evaluation, and goodness are conceptually similar as are activity, arousal, and aggression, and as we discuss below, in the realm of emotion, valence is often taken to be analogous to a {happiness ⇔ sadness} dimension (*13*, *63*).

The linear transformation between VAD and GAS obtained from SVD is

$$\left[\begin{array}{c} \text{Gd}\\ \text{Ag}\\ \text{St} \end{array}\right]≃\left[\begin{array}{ccc} +0.86 & -0.15 & +0.48\\ -0.16 & +0.83 & +0.54\\ +0.48 & +0.55 & -0.69 \end{array}\right]\left[\begin{array}{c} \text{Va}\\ \text{Ar}\\ \text{Dm} \end{array}\right]$$ (2)

In moving to the GAS framework, we have goodness most connected with valence (+0.86) and dominance (+0.48), with a minor negative linkage to arousal (−0.15). Aggression is most connected with arousal (+0.83) and also, like goodness, with dominance (+0.54), but is somewhat at odds with valence (−0.16). What we have identified as an increasing lack of structure corresponds roughly equally to increases in valence and arousal (+0.48 and +0.55) while increasing dominance points in the direction of more structure (−0.69).

We can now see that what separates the GAS framework from the VAD framework (or EPA framework) is that the dominance (or potency) dimension lies within the goodness-aggression plane. That is, the three conceptual dimensions of VAD are in fact collapsed into the two dimensions of goodness and aggression, with a new third and less important dimension of structure being revealed.

When dominance is near zero, Eq. 2 shows that goodness and aggression approximate valence and arousal. However, the correlations between valence and dominance as well as arousal and dominance mean that dominance increasing in magnitude will move goodness-aggression away from valence-arousal.

Returning to the ousiogram in Fig. 2D, we see that the four intercardinal axes carry distinguishable essential meanings, interpolating between the {good ⇔ bad} and {high-aggression ⇔ low-aggression} axes.

The diagonal axis running from “weak” and “empty” to “success” and “triumphant” is a {weak ⇔ powerful} axis, while the orthogonal diagonal axis traveling from “calmness” and “peace” to “murderer” and “homicide” is, we argue, a {safe ⇔ dangerous} axis.

### The PDS framework

For reasons we explain below, we are drawn to consider {weak ⇔ powerful} and {safe ⇔ dangerous} as an alternate essence-of-meaning axes, which we achieve in the third row of ousiograms in Fig. 2 by a simple clockwise rotation of the **Gd-Ag** plane by −π/4. We call this rotation of the GAS framework the PDS framework, and we will then also consider a combined framework: GPADS.

At this stage, we do not view either GAS or PDS to be correct but rather complementary, interrelated frameworks. Even though we of course only need two basis vectors to describe two dimensions, viewing word scores in the combined GPADS framework is valuable.

Expressed as a simple linear transformation, we have

$$\left[\begin{array}{c} \text{Pw}\\ \text{Dg} \end{array}\right]=\frac{1}{\sqrt{2}}\left[\begin{array}{cc} 1 & 1\\ -1 & 1 \end{array}\right]\left[\begin{array}{c} \text{Gd}\\ \text{Ag} \end{array}\right]$$ (3)

**Fig. 3.**
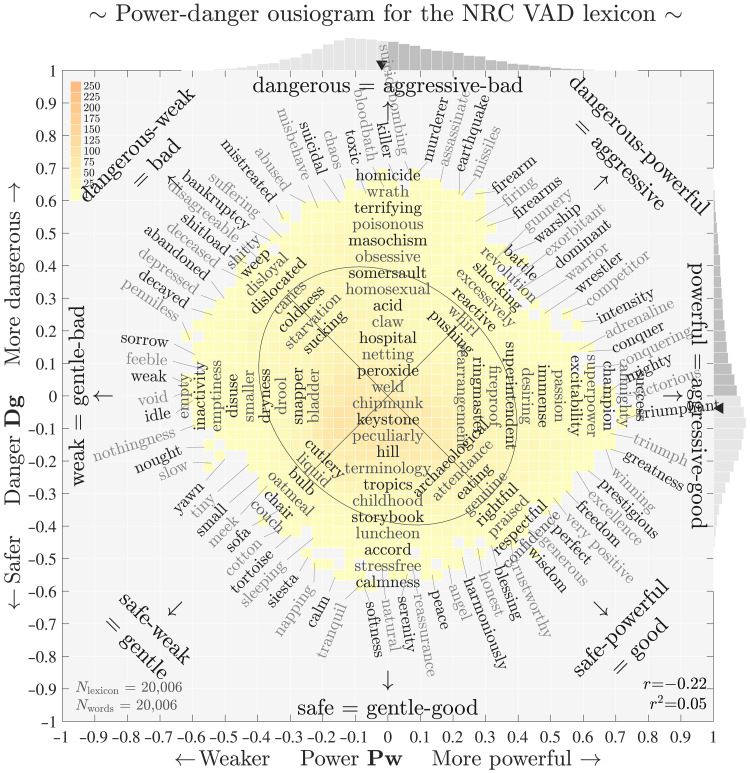
Power-danger ousiogram for the NRC VAD lexicon for the PDS framework, an expanded version of Fig. 2G with internal annotations. The diagonal end points match the axis end points for the GAS framework: safe-powerful ~ good, dangerous-weak ~ bad, powerful-dangerous ~ aggressive, and safe-weak ~ gentle. PDS and GAS interpolate between each other in the primary plane, which can be viewed as a kind of circumplex model, GPADS (*9*), which can be viewed as a cube model (see Fig. 4). Both power and danger reach further into positive values than negative with −0.612 ≤ **Pw** ≤ 0.758 and −0.591 ≤ **Dg** ≤ 0.681. The modes and the medians indicate a slight safe-weak tendency for the meanings of words in the NRC VAD lexicon (medians: −0.019 and −0.038, dark triangles), which is cautioned as an observation preliminary to later measurements where, in accounting for frequency of usage, we find a bias toward safety in real corpora (see Figs. 5 and 6). In the Supplementary Materials, we provide larger ousiograms with internal labels for all corpora and all three frameworks (for the NRC VAD lexicon examples, we use the same color map across all figures).

We supply a more detailed power-danger ousiogram in Fig. 3. When later considering large-scale corpora, we will see that the PDS framework rather than GAS conforms to real word usage (tokens instead of types). However, we first must explore its characteristics for the unamplified NRC VAD lexicon.

In the PDS framework, the variance explained is now evenly divided between power and danger (45.5% each) while structure’s contribution remains the same. Further, the words with the largest magnitude are now aligned with the positive axes of power and danger. For the largest overall magnitude word “success,” the PDS coordinates are (**Pw**, **Dg**, **St**) = (0.76, −0.05, 0.10). For “murderer,” (**Pw**, **Dg**, **St**) = (0.08, 0.68, −0.09). Of the top 20 words by vector magnitude, 12 are strongly aligned with the power direction and 8 are strongly aligned with the danger direction (see figs. S29 and S31). However, we suffer one drawback as we have reintroduced a nonzero correlation, *r* = −0.22, as indicated by the rotated ellipse in Figs. 2G and 3.

The rotated and internal annotations in the power-danger ousiogram in Fig. 3 are now in line with our interpretation of the two axes being {weak ⇔ powerful} and {safe ⇔ dangerous}. The horizontal axis, for example, runs from “void,” “nothingness,” and “empty” to “powerful,” “success,” and “almighty.” We find high danger in “earthquake,” “suicidebombing,” and “toxic,” and safety in “serenity,” “softness,” and “tranquil.”

As for the valence-dominance ousiogram in Fig. 1, traveling around the boundary of the power-danger ousiogram loops us through an ousiometrically sensible sequence of terms. Moving upward and around from “triumphant,” words take on increasingly violent connotations, while moving down, success begins to ebb while peaceful aspects build.

Crucially, and as we have described, the GAS and PDS frameworks form GPADS, a kind of circumplex model. We list the four axes in the primary plane in order from danger at the top of the PD plane, moving clockwise by π/4 through aggression, power, and goodness.

Each of the four directions in GA and PD are mutually intelligible by their adjacent directions in the alternate framework. For example, powerful is aggressive-good, dangerous is aggressive-bad, good is safe-powerful (“wisdom” and “generous”), bad is dangerous-weak (“deceased” and “bankruptcy”), and gentle is weak-safe.

Combining SVD and the −π/4 rotation, we have the linear transformation connecting the VAD and PDS frameworks

$$\left[\begin{array}{c} \text{Pw}\\ \text{Dg}\\ \text{St} \end{array}\right]≃\left[\begin{array}{ccc} +0.50 & +0.48 & +0.72\\ -0.72 & +0.69 & +0.04\\ +0.48 & +0.55 & -0.69 \end{array}\right]\left[\begin{array}{c} \text{Va}\\ \text{Ar}\\ \text{Dm} \end{array}\right]$$ (4)

We see that power is roughly a direct sum of valence, arousal, and dominance (+0.50, +0.48, and +0.72). Danger is a near equally weighted linear combination of negative valence and positive arousal (−0.72 and +0.69), and has little connection to dominance (+0.04). Structure’s connection to VAD remains the same as in Eq. 2 because we have rotated a plane orthogonal to its axis.

We have thus determined that the VAD framework was effectively interpreted as strongly correlated by participants in the NRC VAD lexicon study of Mohammad (*19*). We also now have two complementary frameworks in GAS and PDS that are potential candidates for a fundamental minimal representation of essential meaning.

We emphasize that at this stage, we do not know if the VAD, GAS, or PDS frameworks—or indeed none of them—may be suitable when confronted with real word usage when we consider tokens instead of types. What we do have is a circumplex-like model in GPADS which contains two clear orthogonal frameworks.

### A cube model of meaning for the GPADS framework

**Fig. 4.**
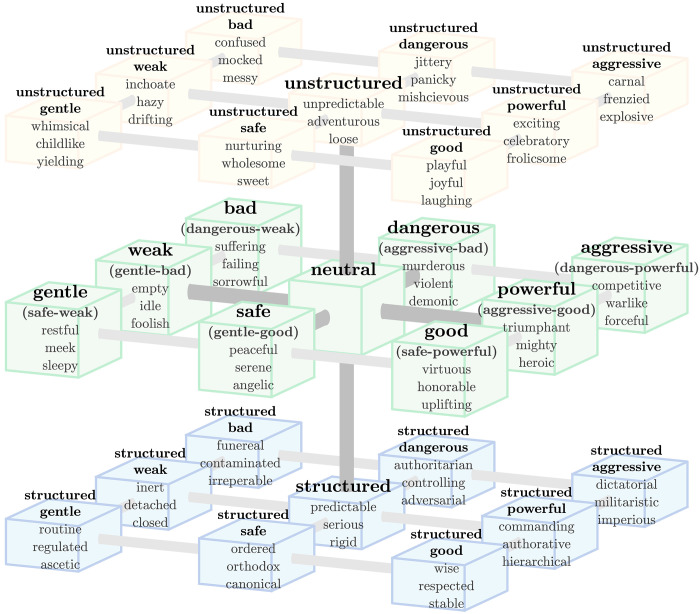
An exploded cube representing the GPADS framework. In figs. S29 to S41, we provide complementary lists of 20 words for each end of the 13 cube axes. Words are ranked by component size conditioned on sufficient alignment. Each of these paired lists indicates a differential with a small version of the exploded cube depicted. The example words on each block are a mixture of words on these lists and adjectives that capture a category. For the cube, blue represents structure (cold, low temperature, and rigid) and yellow represents lack of structure (warm, high temperature, and loose), while block thickness qualitatively indicates variance explained.

In Fig. 4, we combine all of our framework identifications in a unified GPADS cube model of essential meaning. The middle primary plane shows the four interconnected axes of GAS and PDS: GPAD. In anticipation of our later findings, we have aligned the cube within the PDS framework, though it could be readily rotated to align with GAS.

The cube model is complementary to the map-like ousiograms in Fig. 3 and figs. S1 to S9 and is also informed by and connected with lists of ranked words in figs. S29 to S44. For the word lists, we show the top 20 words by component size for each of the 13 cube axes, 9 of which connect the structured and unstructured levels. Three axes connect face cubes, six connect edge cubes, and four connect corner cubes. To avoid overlap, we restrict words to cones around axes of half-angle $\frac{1}{2}\frac{180}{\pi }{\text{cos}}^{-1}\left(2/\sqrt{6}\right)≃17.6$. For example, Fig. 5 shows the top 20 words for unstructured-gentle versus structured-aggressive, which is the axis connecting the corner cubes in the front top left and back lower right.

**Fig. 5.**
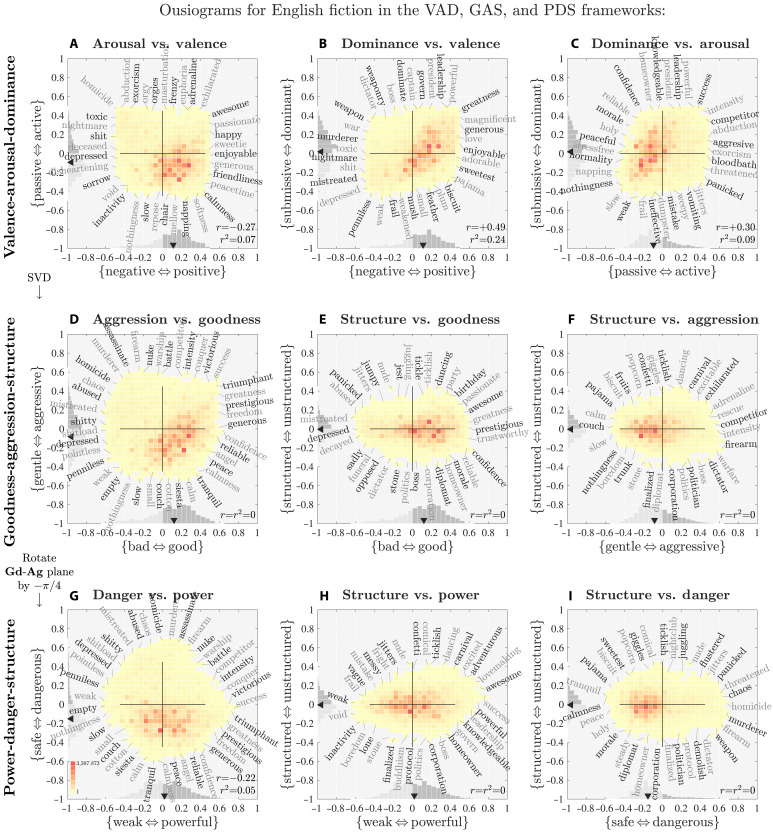
Ousiograms for English fiction (1900 to 2019) arranged in the same analytic sequence format as Fig. 2. We now allow each word’s contribution to be its overall frequency of usage within a given corpus. We form a single frequency-rank distribution (*51*) for the entire corpus by equally weighting each year’s frequency-rank distribution (*52*, *53*). The sequence indicates that (i) overall, the Google Books English fiction corpus is best aligned with the PDS framework, and (ii) expressed language exhibits a “safety bias,” a generalization of the Pollyanna principle (*45*, *48*, *64*). Row 1 (**A** to **C**): In the VAD framework, the histograms are clearly misaligned with the main axes. Row 2 (**D** to **F**): The histograms are again poorly aligned with the main axes of **Gd**, **Ag**, and **St**. The marginal distributions for Goodness and Aggression in (D) show an apparent “goodness bias” and a “low-aggression bias.” The goodness bias is an instantiation of the Pollyanna principle for language (*45*, *48*). Row 3 (**G** to **I**): Rotation to the power-danger framework shows that words used in English fiction conform to a safety bias with the preponderance of words falling on the safe side of the power-danger plane (G). Both the goodness and aggression biases in (D) are revealed to be one-dimensional projections of an underlying safety bias. Words are distributed broadly in the power-structure plane (H) and are on the safe side of the danger-structure plane (I).

For each cube (except the neutral one), we show three adjectives that capture the words associated with that cube and their location within the GPADS framework. The adjectives are in some cases words directly taken from the relevant list but more usually are general descriptors.

### The linguistic “safety bias” of disparate large-scale corpora

Having established the GAS and PDS frameworks as alternatives to VAD, we turn to real, large-scale corpora. By intent, we have so far only considered the essential meaning of words and terms in the NRC VAD lexicon—the level of types.

We now aim to incorporate frequency of usage of words—tokens—for a collection of well-defined corpora. We can only do so sensibly within each structured corpus—we cannot meaningfully combine, for example, *The New York Times* and Twitter.

For an initial example corpus, we investigate the ousiometric content of 1-grams used in English fiction from 1900 to 2019 per the Google Books project (*52*). We note that we have earlier argued and demonstrated that the Google Books project generates problematic corpora in that (i) each book is in principle counted once (popularity is not measured) and (ii) for all English books combined, the corpus is clouded by a growing preponderance of scientific literature (*53*). To use the framing of types and tokens for the former point, the books are themselves types, containing *n*-grams as tokens, but we do not have the books as tokens by knowing, for example, numbers of copies sold. Nevertheless, for our purposes here, the relatively science-free 2019 English fiction corpus provides a raw large-scale body of text to examine.

We generate ousiograms in the same fashion as before, but we now weight words by their frequency of usage. The NRC VAD lexicon acts as a lexical lens on the frequency-rank distribution—we only take word counts for those words we have VAD/GAS/PDS scores for. In Fig. 5, we reprise the analytic sequence of Fig. 2 for words used in English fiction.

While the histograms were relatively uniform for the NRC VAD lexicon, we now see uneven distributions. For the VAD row, we see the distributions are not aligned with the underlying axes of the VAD framework (Fig. 5, A to C). The main ousiogram for goodness-aggression (Fig. 5D) still does not align well, showing an off-axis bias toward goodness and low aggression, the former being a linguistic signature of the Pollyanna principle (*45*, *48*, *64*). We discuss both biases further below. The goodness-structure and aggression-structure ousiograms (Fig. 5, E and F) show biases toward goodness and low aggression that appear more aligned.

It is in the PDS framework (Fig. 5, G to I) that we see robust agreement between ousiograms and the underlying axes. In the main power-danger ousiogram (Fig. 5G), the histogram shows a definitive bias toward safe, low-danger words. As shown by the marginal on the left axis, the danger distribution is skewed strongly toward safer words, and the median danger score is well below 0. By contrast, power presents a symmetric marginal distribution with a median slightly above 0. The power-structure ousiogram shows a general spread (Fig. 5H) while the danger-structure ousiogram again shows a clear safety bias (Fig. 5I).

**Fig. 6.**
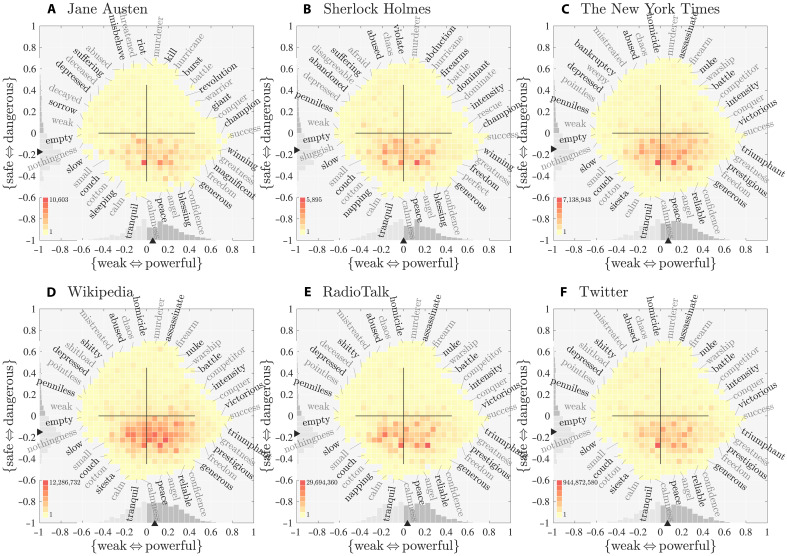
Ousiograms for power-danger space for six corpora of varying type and scale. The corpora are (**A**) Jane Austen’s novels; (**B**) Arthur Conan Doyle’s Sherlock Holmes novels and short stories; (**C**) *The New York Times* (1987 to 2007) (*54*); (**D**) Wikipedia (March 2019) (*55*); (**E**) Talk radio transcripts (October 2018 to March 2019) (*56*); and (**F**) Twitter (approximately 10% of all English tweets in 2020, with each day weighted equally) (*54*, *82*). Words of the six corpora all strongly canvass power-danger space with a marked bias toward safe. Jane Austen’s novels, *The New York Times*, and Wikipedia are all author-side corpora in that their frequency-rank distributions do not incorporate popularity of books, sections, or entries. By contrast, Twitter incorporates a reader-side measure of popularity through amplification by retweets. Each ousiogram’s color map is linearly normalized to the highest count bin, and the maximum bin count is indicated at the top of each color bar. The highest count bin in (A), (C), and (F) is due to the word “be” (**Pw** = −0.001, **Dg** = −0.300). See the “Description of datasets” section. For the six corpora here, we provide the full VAD-GAS-PDS analytic sequence of Figs. 2 and 5 in figs. S42 to S47.

In Fig. 6, we expand our analysis to show power-danger ousiograms for six more corpora: the novels of Jane Austen, a subset of Arthur Conan Doyle’s Sherlock Holmes stories, *The New York Times*, Wikipedia, transcriptions of talk radio in the United States, and Twitter. These corpora vary widely in size and kind: written versus spoken, news, literature, formal and informal, bearing social amplification or not (e.g., the inclusion of retweets from Twitter encodes one form of echoing, but the other corpora carry no such equivalent signature of popularity). For each corpus, we provide the full analytic sequence in the manner of Figs. 2 and 5 in figs. S42 to S47.

The power-danger ousiograms for these six distinct corpora in Fig. 6 all present the same safety bias for words as we saw for English fiction in Fig. 5G. While varying in detail as they must, the six histograms in Fig. 6 all show a weight toward words below the horizontal {weak ⇔ powerful} axis, and the danger marginals on the left axes of all ousiograms are skewed toward safety. There is no such bias for the power dimension, though median power is at or above zero in all cases.

We emphasize again that our initial determination of the PDS framework was performed only at the level of types, using the NRC VAD lexicon. In these subsequent tests with real corpora, we have found that our hypothesized ousiometric PDS framework has been borne out to be fundamental.

### Congruences: Russell’s circumplex model of emotion

We consider Russell’s highly cited circumplex model of affect (*9*), in light of the GPADS framework. We find general accordance with one region of disagreement being in the aggressive direction (the dangerous-powerful quadrant).

Affective states are representations of emotional states, and may be external (e.g., facial expressions) or internal (conscious awareness). In linking to essential meaning, in 1952, Schlosberg (*65*) was one of the first to suggest that emotion—as conveyed by facial expressions—could be well represented by two dimensions, suggesting {pleasantness ⇔ unpleasantness} and {attention ⇔ rejection}. Two years later, Schlosberg then posited a third dimension of level of activation while also asserting that “the field [of emotion] is chaotic” (*66*). Certain emotions would seem to readily connect with locations in the power-danger framework. Fear is a particular response to danger, contentment is a possible state in a safe environment, and so on. We examine such connections carefully below.

We consider Russell’s original, unrevised model because of its historical and continued importance to the field (*27*, *28*, *67*, *68*) as well as the challenge delivered by such a distinct kind of study. The approximate agreement between the studies of Russell and Mohammad is remarkable given the differences between them: era (late 1970s versus late 2010s), participants (undergraduate students at the University of British Columbia versus online crowdsourcing), assessment type [various in (*9*) versus best-worst scaling], scale (28 versus ~20,000 terms), and framing (the specific of affect versus the general of essential meaning).

**Fig. 7.**
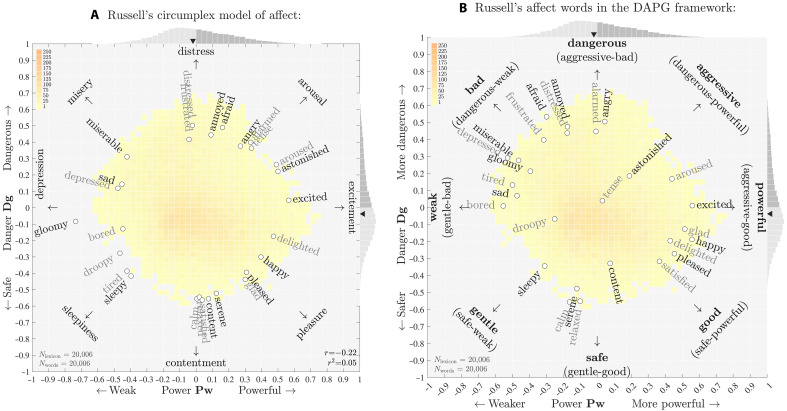
Comparison of Russell’s two-dimensional circumplex model of affect with the GPAD framework. The two frameworks show impressive agreement given how differently words were scored in (*9*) and (*19*). (**A**) Reconstruction of the circumplex model scores for 27 affect words for the first survey presented in figure 2 in (*9*). We obtained data for 27 of 28 terms by visual inspection of figure 2 in (*9*), omitting the 2-gram “at ease.” To enable comparison, we rotate Russell’s scores by −π/4, and also underlie both plots with the power-danger histogram per Fig. 3. The comparison is nevertheless with the GPAD framework, and the PD alignment is for consistency. Leaving angles unchanged, we uniformly rescale the magnitude of scores for the 27 affect words in the circumplex model to give an approximate fit to the power-danger scale—only angles and relative magnitudes may be sensibly compared. (**B**) Locations in the power-danger plane for the 27 affect words of (*9*), all of which are also found in the NRC VAD lexicon. We indicate the full GPAD framework with directions.

Building on earlier work (*65*, *69*), Russell asserted that eight fundamental affect concepts could be arranged as compass points on a circle [see figure 1 in (*9*)]. As we indicate in Fig. 7A, starting from pointing upward and stepping around clockwise, these concepts are distress (~ danger), arousal (~ aggression), excitement (~ power), pleasure (~ goodness), contentment (~ safety), sleepiness (~ gentleness), depression (~ weakness), and misery (~ badness). In line with the VAD framework, Russell took the underlying horizontal and vertical dimensions to be {pleasure ⇔ displeasure} and {arousal ⇔ sleepiness}, aligning in the GPAD frameworks {goodness ⇔ badness} and {aggression ⇔ gentleness}. The alignment with the GAS framework notwithstanding, we have facilitated comparison with the GPAD framework, by rotating Russell’s framework by −π/4.

Russell then carried out a series of varying types of surveys on perceptions of 28 affect terms (e.g., “afraid,” “glad,” “serene,” and “bored”). In Fig. 7A, we show the locations of 27 words according to the results presented in figure 2 of (*9*) (we exclude the 2-gram “at ease”). In Fig. 7B, we show the same words located by their power-danger scores.

In general, we see that words in the circumplex and GPAD frameworks are reasonably well aligned. A number of words show strong congruence across the two studies, including “sleepy,” “excited,” “aroused,” and “miserable.” Angles of affect words are generally similar with a maximum discrepancy of around π/4. For example, “tired” is in the direction of gentle in the circumplex model and weak in the GPAD framework (“sleepy” aligns with gentle in both, and the added hue of danger for “tired” in the GPAD framework is sensible). Apart from “tense” and, to a lesser extent, “astonished” and “droopy,” affect words register strong power-danger magnitudes and are consequently located around an approximate circle.

The word that most stands out as differing between the two studies is “tense.” On top of the major distinctions between the studies listed above, without the context of working with a small set of emotion-themed words, participants in the NRC VAD study would be more likely to interpret words and phrases by their most general, dominant meaning. While many of the affect words have clear meanings that are emotional (e.g., “miserable”), the word “tense” might not be as strongly construed as “stressed.” Over four decades, we might also expect meanings of some words to shift somewhat. In any case, the four surveys in Russell only show rough agreement with each other [see figures 2 to 5 in (*9*)].

### Congruences: Fictional characters

In separate work—archetypometrics—we have carried out extensive analysis of a dataset of 2000 characters from 341 popular stories from literature, movies, and television that have more than 70 million ratings across 464 semantic differential traits (*50*, *70*). As per our methodology here, we performed SVD on the 464 × 2000 matrix and then generated a suite of ousiograms and ranked lists for both essential trait and character space. Here, we focus on a few core elements of essential trait space.

**Fig. 8.**
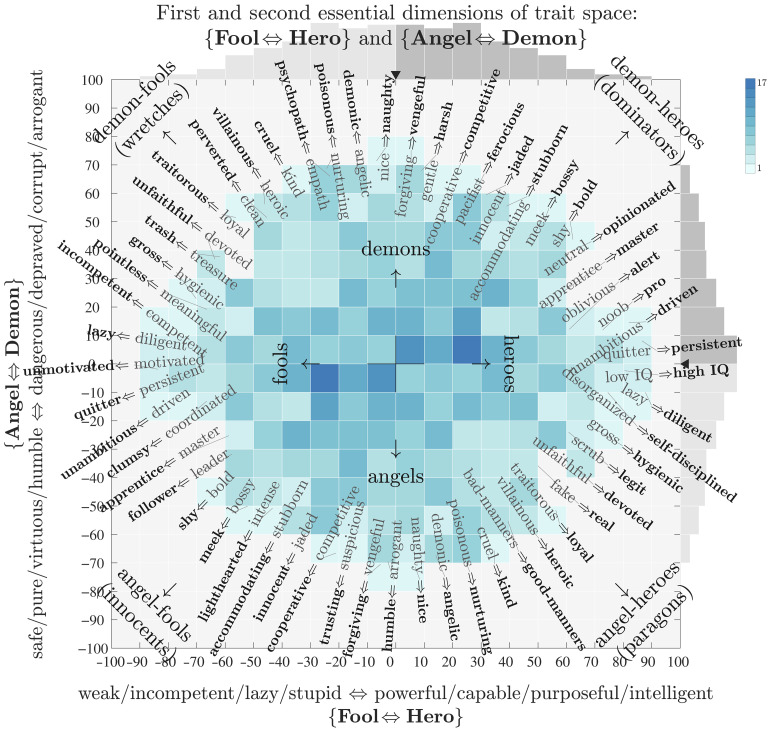
Ousiogram for the first two singular dimensions of essential trait space based on 2000 fictional characters evaluated across 464 traits each. We observe a clear congruence between the GPADS framework and the primary archetypes of fictional characters. Compare with Table 1, which lists the top ranked traits for the first three dimensions. The first three dimensions of essential trait space align with PDS as {Fool ⇔ Hero} for {weak ⇔ powerful}, {Angel ⇔ Demon} for {safe ⇔ dangerous}, and {Traditionalist ⇔ Adventurer} for {structured ⇔ unstructured}. Because the orientation of each trait’s semantic differentials was arbitrary, we duplicated all semantic differentials with their bipolar adjectival pairs swapped. Hence, the traits and their reverse appear on opposite sides of the ousiogram. See (*50*) for a full exploration of archetype space with analysis that shows the first six singular dimensions rise to the level of identifiable archetypes.

In Fig. 8, we show an ousiogram for the first two singular dimensions of essential trait space, and in Table 1, we list the top 15 traits for the first three dimensions.

**Table 1.** The leading trait composition for the three primary dimensions of archetype space (*50*). Traits are ranked by component size in each dimension. Compare with the ousiogram in Fig. 8 for the first two dimensions.

Essential trait dimension 1 {Fool ⇔ Hero} ~ {weak ⇔ powerful}	Essential trait dimension 2 {Angel ⇔ Demon} ~ {safe ⇔ dangerous}	Essential trait dimension 3 {Traditionalist ⇔ Adventurer} ~ {structured ⇔ unstructured}
1. {lazy ⇔ diligent}	1. {nice ⇔ naughty}	1. {scheduled ⇔ spontaneous}
2. {quitter ⇔ persistent}	2. {forgiving ⇔ vengeful}	2. {stick-in-the-mud ⇔ adventurous}
3. {unmotivated ⇔ motivated}	3. {humble ⇔ arrogant}	3. {uncreative ⇔ open to new experiences}
4. {unambitious ⇔ driven}	4. {nurturing ⇔ poisonous}	4. {serious ⇔ bold}
5. {incompetent ⇔ competent}	5. {gentle ⇔ harsh}	5. {monotone ⇔ expressive}
6. {low IQ ⇔ high IQ}	6. {angelic ⇔ demonic}	6. {lifeless ⇔ spirited}
7. {absentminded ⇔ focused}	7. {warm ⇔ quarrelsome}	7. {corporate ⇔ freelance}
8. {helpless ⇔ resourceful}	8. {cooperative ⇔ competitive}	8. {geriatric ⇔ vibrant}
9. {unobservant ⇔ perceptive}	9. {empath ⇔ psychopath}	9. {serious ⇔ playful}
10. {slacker ⇔ workaholic}	10. {kind ⇔ cruel}	10. {stoic ⇔ expressive}
11. {disorganized ⇔ self-disciplined}	11. {wholesome ⇔ salacious}	11. {shy ⇔ playful}
12. {noob ⇔ pro}	12. {altruistic ⇔ selfish}	12. {humorless ⇔ funny}
13. {slugabed ⇔ go-getter}	13. {sweet ⇔ bitter}	13. {deliberate ⇔ spontaneous}
14. {underachiever ⇔ overachiever}	14. {respectful ⇔ rude}	14. {orderly ⇔ chaotic}
15. {gross ⇔ hygienic	15. {pure ⇔ debased}	15. {withdrawn ⇔ outgoing}

The PDS framework is aligned with the first three dimensions of archetype space. We emphasize that we again kept the factor analysis simple, and that we did not perform any rotations or further manipulations.

We have named the archetype pairs of the first three dimensions {Fool ⇔ Hero}, {Angel ⇔ Demon}, and {Traditionalist ⇔ Adventurer}. For even more agreement, we find that in the GPADS framework, the words “foolish,” “heroic,” “angelic,” and “demonic” are all located in strong alignment with their corresponding archetypes in essential trait space. We have included these words as descriptors in the meaning cube in Fig. 4.

These archetype names are of course general names that may register variably. We further define them by distilling the dominant traits into four pairs of semantic differentials each. For {Fool ⇔ Hero}, we have {weak ⇔ powerful}, {incompetent ⇔ capable}, {lazy ⇔ purposeful}, and {stupid ⇔ intelligent}; for {Angel ⇔ Demon}, we have {safe ⇔ dangerous}, {pure ⇔ depraved}, {virtuous ⇔ corrupt}, and {humble ⇔ arrogant}; and for {Traditionalist ⇔ Adventurer}, we have {serious ⇔ playful}, {predictable ⇔ unpredictable}, {humorless ⇔ funny}, and {uncreative ⇔ creative}.

One differential that might first appear at odds with our framework is {villainous ⇔ heroic}, which is aligned with {bad ⇔ good}. However, the differential correctly interpolates between the directions of {demon} (villain) and {hero}, which are not opposites but rather at right angles to each other. That is, {villainous ⇔ heroic} ~ {weak ⇔ powerful} − {safe ⇔ dangerous} = {bad ⇔ good} .

Last, for a circumplex model of archetypes that is aligned with the GPAD framework, and as indicated in Fig. 8, we suggest the following (moving around the circle starting at the top): Demons (dangerous), Dominators (aggressive), Heroes (powerful), Paragons (good), Angels (safe), Innocents (or Lambs) (gentle), Fools (weak), and Wretches (bad).

### Ousiometer

We construct an elementary “ousiometer,” a lexical instrument for measuring the average essential meaning of large-scale texts. We take a similar approach to that of our hedonometer (*14*, *45*, *63*, *71*–*73*). We view the ousiometer and hedonometer as example “telegnomic” lexical instruments capable of remotely sensing meaning, knowledge, and stories.

We use *M* to represent one of the essential meaning dimensions within a specified ousiometric framework. For a simple ousiometer, we compute the average meaning score *M*_avg_(Ω) for a text Ω in the following way. We consider only the 1-grams of the NRC VAD lexicon, leaving aside *n*-grams for *n* ≥ 2 for possible future improvements. For any given text Ω, we apply a “lexical lens” L, a simple operator that filters the text’s 1-grams, returning the subset 1-gram lexicon that intersects with the NRC VAD 1-gram lexicon. We denote the lensed text as L(Ω). We write the resultant lensed lexicon as *R*_L(Ω)_, further specifying this set to be a list of 1-grams ordered by descending frequency of usage *f*_τ_ within L(Ω). For each 1-gram τ in the lensed lexicon *R*_L(Ω)_, we then straightforwardly determine τ’s normalized frequency as $p_{\tau }=f_{\tau }/\sum_{\tau^{\prime }}f_{\tau^{\prime }}.$

In general, given a lexical lens L, the average ousiometric score of a text Ω is

$$M_{\text{avg}}\left(\Omega ;L\right)=\sum_{\tau \in R_{L\left(\Omega \right)}}p_{\tau }M_{\tau }$$ (5)

where *M*_τ_ is the average ousiometric score for the 1-gram τ derived from the NRC VAD lexicon scores (*19*).

As an example, in Fig. 2, we show ousiometric trajectory and time series for Victor Hugo’s *Les Misérables*. In doing so, we are continuing the development of our earlier computational work on the measurement of emotional arcs in stories, famously ventured by K. Vonnegut (*45*, *72*, *74*, *75*).

The main plot is an ousiometric trajectory of *Les Misérables* in the GPAD framework, oriented in the PD plane. To the right, we display ousiometric time series for the GAS framework that interlocks with the same for the PDS framework running along the bottom. The colors indicate 10 narrative reading time blocks, as measured by 1-grams, and help show the ousiometric trajectories path in time.

**Fig. 9.**
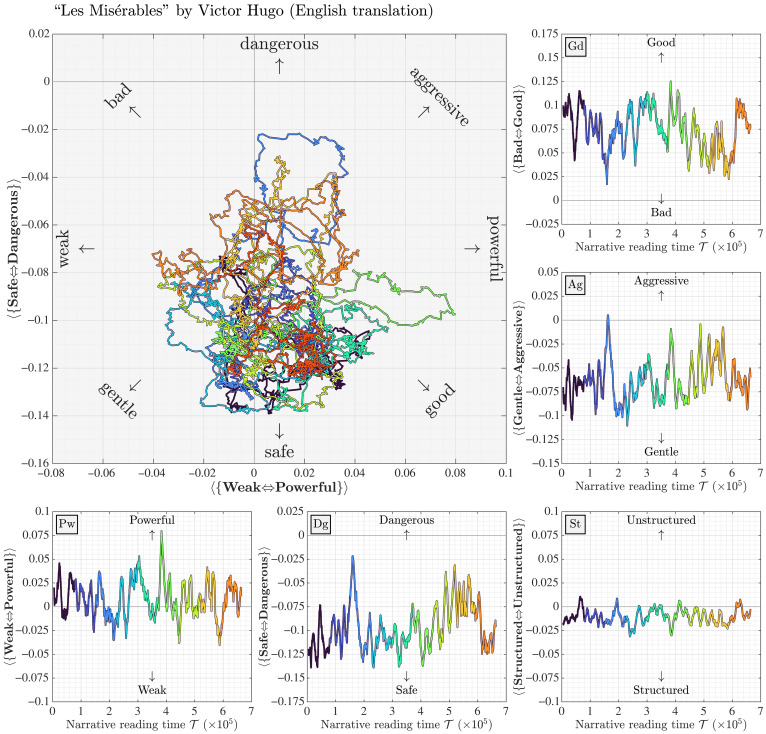
The ousiometer as a telegnomic lexical instrument for literature: Example ousiometric time series for Victor Hugo’s *Les Misérables*. Main plot: Ousiometric trajectory of the novel in the PD plane with the GPAD framework indicated. The five individual time series comprise the GPADS framework. We break the timeline into 10 equal epochs and align the colors on all time series, including that of the trajectory in the PD plane. Narrative reading time T is in terms of 1-grams, which includes numbers, punctuation, and other nonword elements. All vertical ranges for the time series are the same (0.20) and are shifted as needed. As expected per variance explained, the first four time series of the GPADS framework show similar variation while Structure is muted. Smoothing is at 10,000 1-grams with steps of 100 1-grams. See the accompanying flipbook in figs. S48 to S72.

We observe that the {bad ⇔ good} time series is of similar form to what we found using our hedonometer (*72*). In fig. S73, we show a screenshot for the happiness time series for *Les Misérables* taken from our interactive story viewer at https://hedonometer.org. The agreement is satisfactory given that the hedonometer and ousiometer instruments are built on two distinct word lists using different evaluations (Likert versus best-worst scaling). An interactive visualization based on Fig. 9 would be a natural next step.

In figs. S48 to S72, we supply a flipbook that traces out the GPADS time series over 25 epochs. A full analysis of our ousiometric time series for *Les Misérables* is well beyond the scope of our present work, and we reserve it for future exploration. Our purpose here is simply to show that we can readily build an ousiometer for large-scale texts.

In connected work on the ousiometrics of literature (*75*), we have used empirical mode decomposition on ousiometric time series to find that longer books are structured more like concatenations of shorter texts, revealing characteristic fluctuations in power and danger. Last, in the Supplementary Materials, we provide another example use of an ousiometer for streaming text: a retrospective analysis of Twitter for January 2020 to January 2021 (*76*–*81*).

### Concluding remarks

#### The mismeasurement of meaning

The quantitative measurement of essential meaning—ousiometrics—has been properly engaged as a scientific challenge for close to a century. On the basis of semantic differentials, the three-dimensional orthogonal framework of EPA due to Osgood *et al.* (*6*) has effectively remained the leading conceptual framework, if not always by direct reference. Research into the specific context of affect saw EPA adapted as VAD (*11*, *12*). The VAD formalism has become widespread and not limited to studies of emotion, even being used for general essential meaning studies instead of EPA (*13*, *19*).

We have shown that ~20,000 terms evaluated by best-worst scaling in the VAD framework fails to reproduce the orthogonal VAD framework itself. We have contended that this cannot be explained away by participants misunderstanding bipolar adjectival pairs used to define VAD dimensions. Rather, we have argued that a long-standing problem for ousiometrics has been the difficulty of ascribing bipolar adjectival pairs to accurately characterize dimensions derived from participants’ assessments of a larger set of semantic differentials (*44*). As is, researchers tend to provide sets of bipolar adjectival pairs for fundamental dimensions, making them overly blunt instruments that have more likelihood of being correlated (see table S1). Even after exploring antonyms and antousionyms (see the Supplementary Materials), we continue to see this dimension characterization problem as unavoidable.

We recommend that ousiometric studies start from a larger set—on the order of 20 to 50—of simple bipolar adjectival pairs and always perform dimensional reduction. Standardizing such a set of clear bipolar adjectival pairs would be of great value to the field, and our advice is independent of which instrument is used to rate semantic differentials (Likert scale, best-worst scaling, etc.). (Likert scales may have less interrater reliability than best-worst scaling but do allow datasets to be extended with independent studies.) Such studies will be more expensive but will be far more robust. Using ousiograms, which provide richly informative visualizations, the extracted dimensions can then be examined and identified. For lexicons sufficiently rich in types and corpora-matching in terms of tokens, we expect that the axes of {weak ⇔ powerful} and {safe ⇔ dangerous} will emerge.

Automatically annotated histograms like our ousiograms could be used in any sphere to compare two variables measured for a collection of categorical entities (e.g., crime rates and median house prices for cities in the United States).

#### The GAS, PDS, and GPADS frameworks

Here, we have found that essential meaning does not conform to VAD but is instead well captured by the two mutually interpretable coordinate systems spanned by the broad semantic differentials {bad ⇔ good} and {gentle ⇔ aggressive} (GAS), and {weak ⇔ powerful} and {safe ⇔ dangerous} (PDS). Both share the dimension of essential meaning—{structured ⇔ unstructured}—which we may interpret as the dimension of evolution. We have argued in particular that the primary two-dimensional plane, which accounts for more than 90% of variance explained for types, can be viewed as a kind of circumplex model giving us GAPDS. We note that one of the 50 semantic differentials used by Osgood *et al.* (*6*) in their foundational work was {safe ⇔ dangerous}. We now understand that the dimension Activation in the EPA framework was an error. All dimensions have an intensity level signified by vector magnitude.

#### The safety bias of communication

Our finding of a safety bias in diverse written and spoken language generalizes our earlier work, which revealed a positivity bias (*45*, *64*)—a linguistic instantiation of the Pollyanna principle (*48*). In the GAS framework we have defined here, the positivity bias is a goodness bias. We have also found a complementary linguistic low-aggression bias in the GAS framework (see Fig. 5D).

We now understand that the linguistic goodness bias and the linguistic low-aggression bias are shadows of an underlying linguistic safety bias—projections of points in the two-dimensional Pw**-**Dg plane onto the orthogonal one-dimensional diagonal axes of goodness and aggression. The one-dimensional map is not the two-dimensional territory.

Because of the safety bias, congruences with other spaces like fictional archetypes, and the behavior of our prototype ousiometer, we have demonstrated that the PDS framework is the most minimal, well-aligned description of essential meaning. This does not however lead us to discarding the GAS framework as the GPADS circumplex framework affords richer, more immediately informative analyses than either alone.

## Supplementary Materials

### The PDF file includes:

Sections S1 to S10 Tables S1 and S2 Figs. S1 to S74 References
