# Supplementary material for: Ousiometrics: The essence of meaning aligns with a power-danger-structure framework instead of valence-arousal-dominance
Source: Sci Adv. 2026 May 6;12(19):eadr4039. doi: 10.1126/sciadv.adr4039 (PMC13148342; doi:10.1126/sciadv.adr4039)
Supplement: Supplementary file 1 — Sections S1 to S10 Tables S1 and S2 Figs. S1 to S74 References [file sciadv.adr4039_sm.pdf]

Science Advances
AAAS

Supplementary Materials for

Ousiometrics: The essence of meaning aligns with a power-danger-structure framework instead of valence-arousal-dominance

Peter Sheridan Dodds *et al.*

Corresponding author: Peter Sheridan Dodds, peter.dodds@uvm.edu

Sci. Adv. **12**, eadr4039 (2026)
DOI: 10.1126/sciadv.adr4039

The PDF file includes:

Sections S1 to S10
Tables S1 and S2
Figs. S1 to S74
References

Other Supplementary Material for this manuscript includes the following:

Data S1

S1 Large ousiograms for VAD, GAS, and PDS

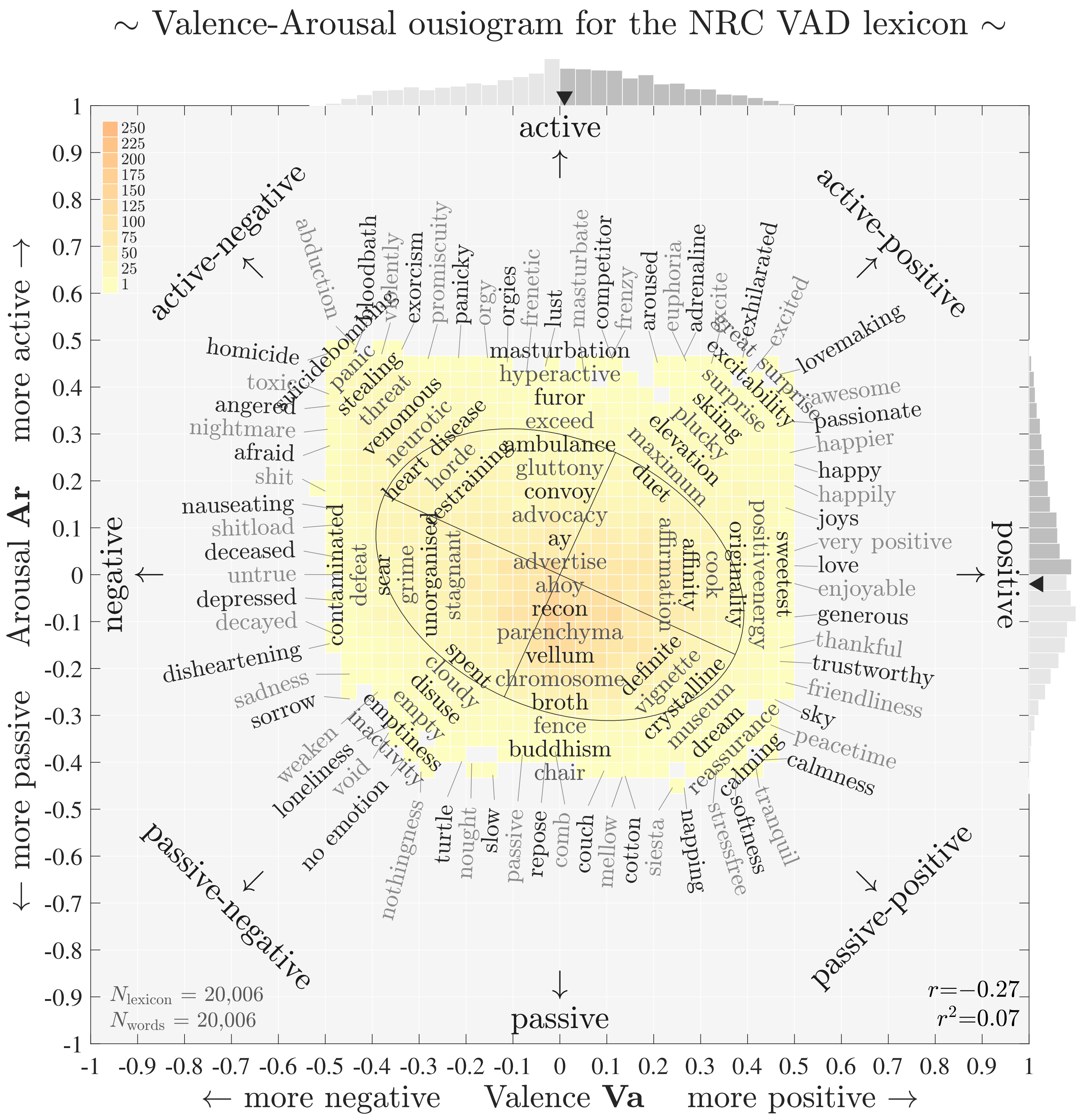

Figure S1: Ousiogram for arousal vs. valence in the VAD framework.

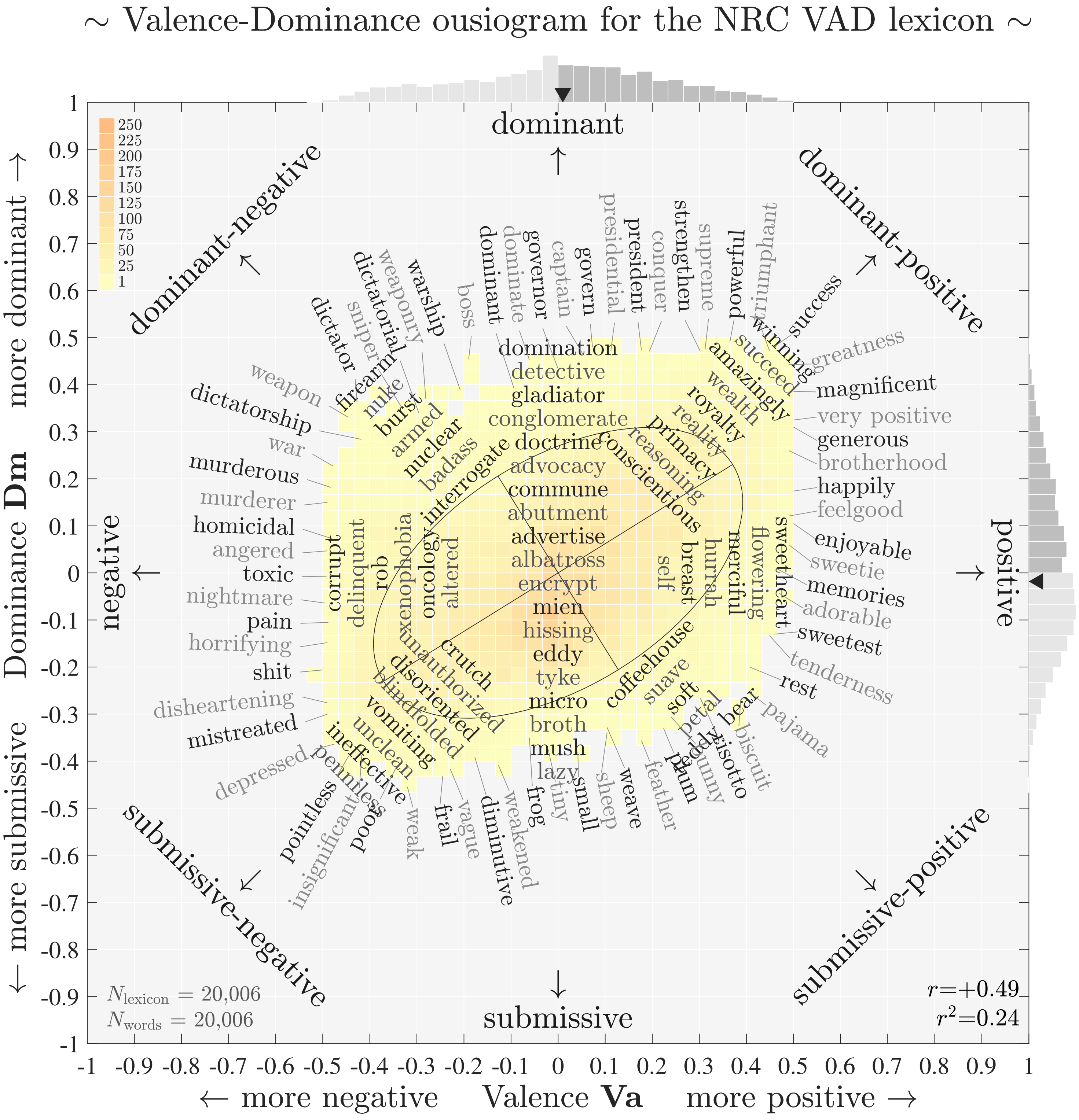

Figure S2: Ousiogram for dominance vs. valence in the VAD framework. Matches Fig. 1 in the main paper.

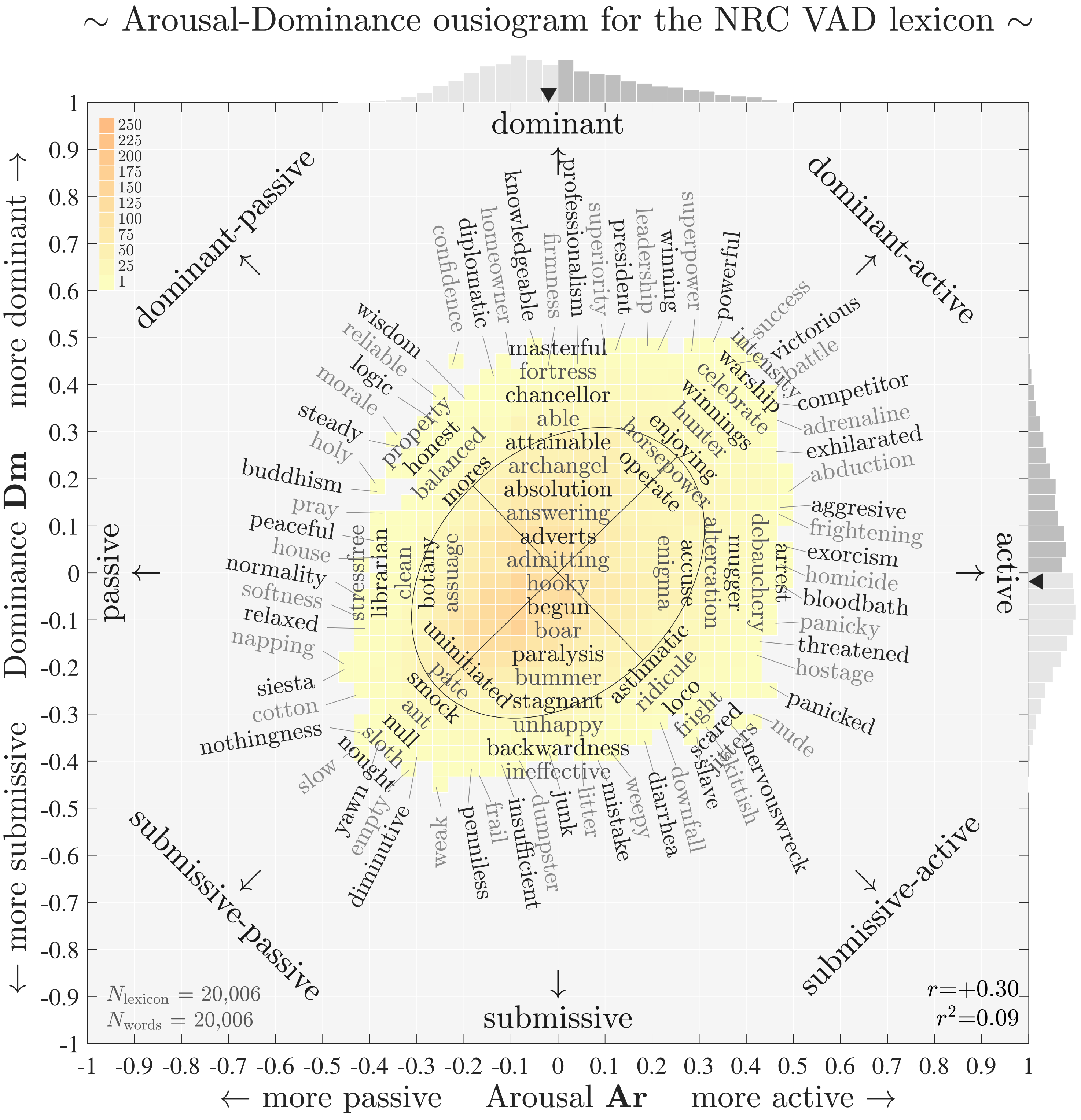

Figure S3: Ousiogram for arousal vs. dominance in the VAD framework.

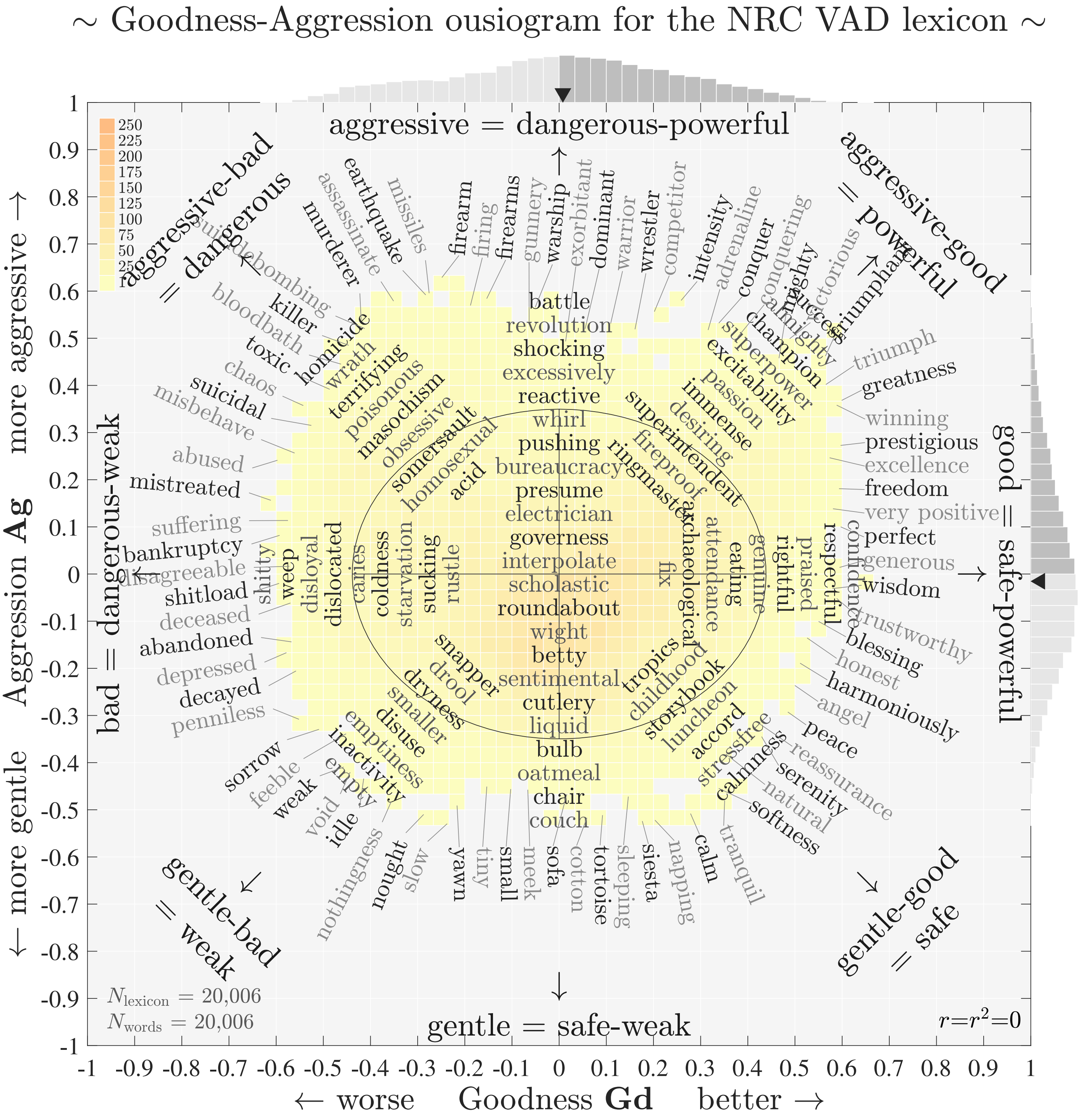

Figure S4: Ousiogram for aggression vs. goodness in the GAS framework.

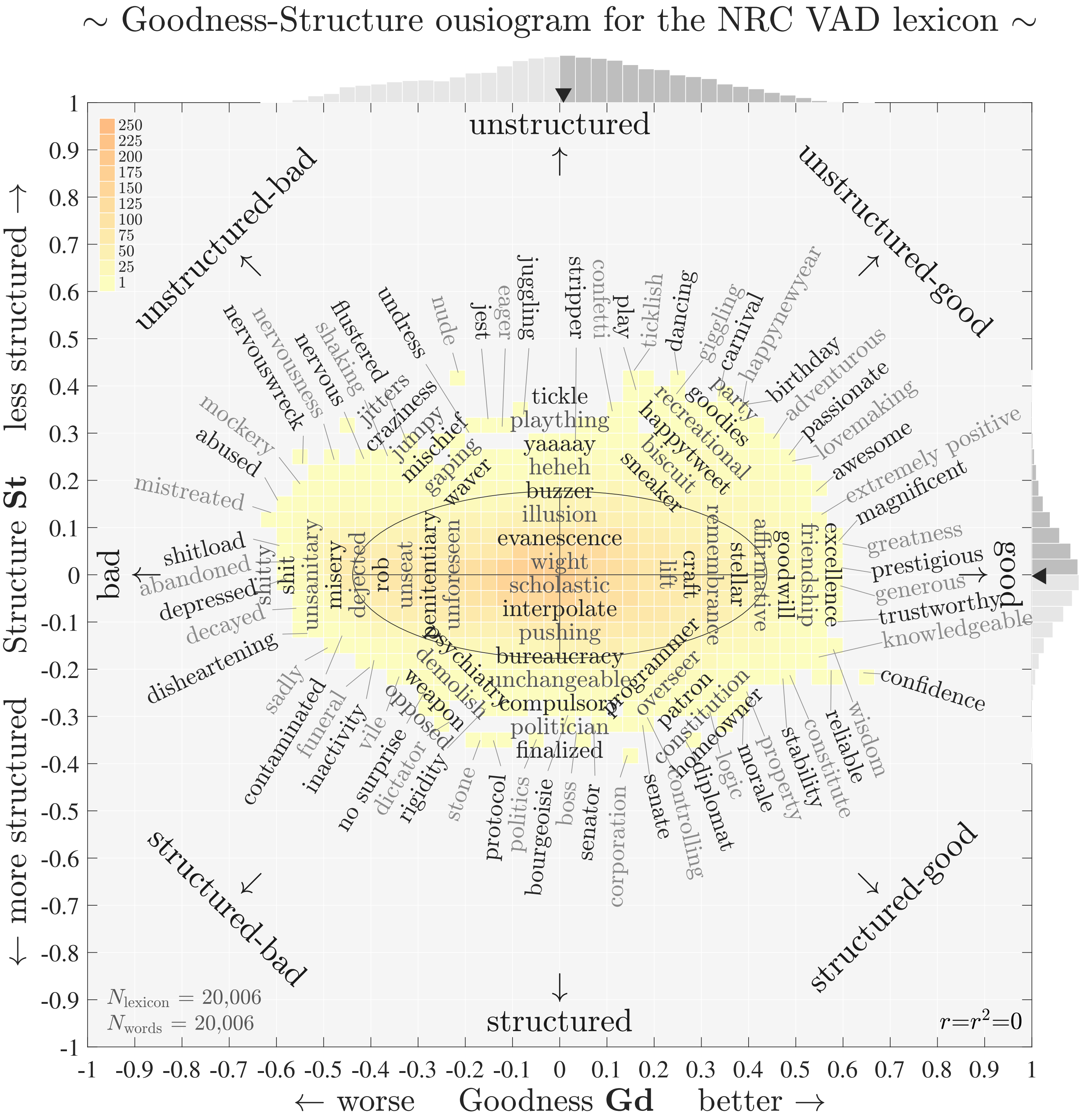

Figure S5: Ousiogram for structure vs. goodness in the GAS framework.

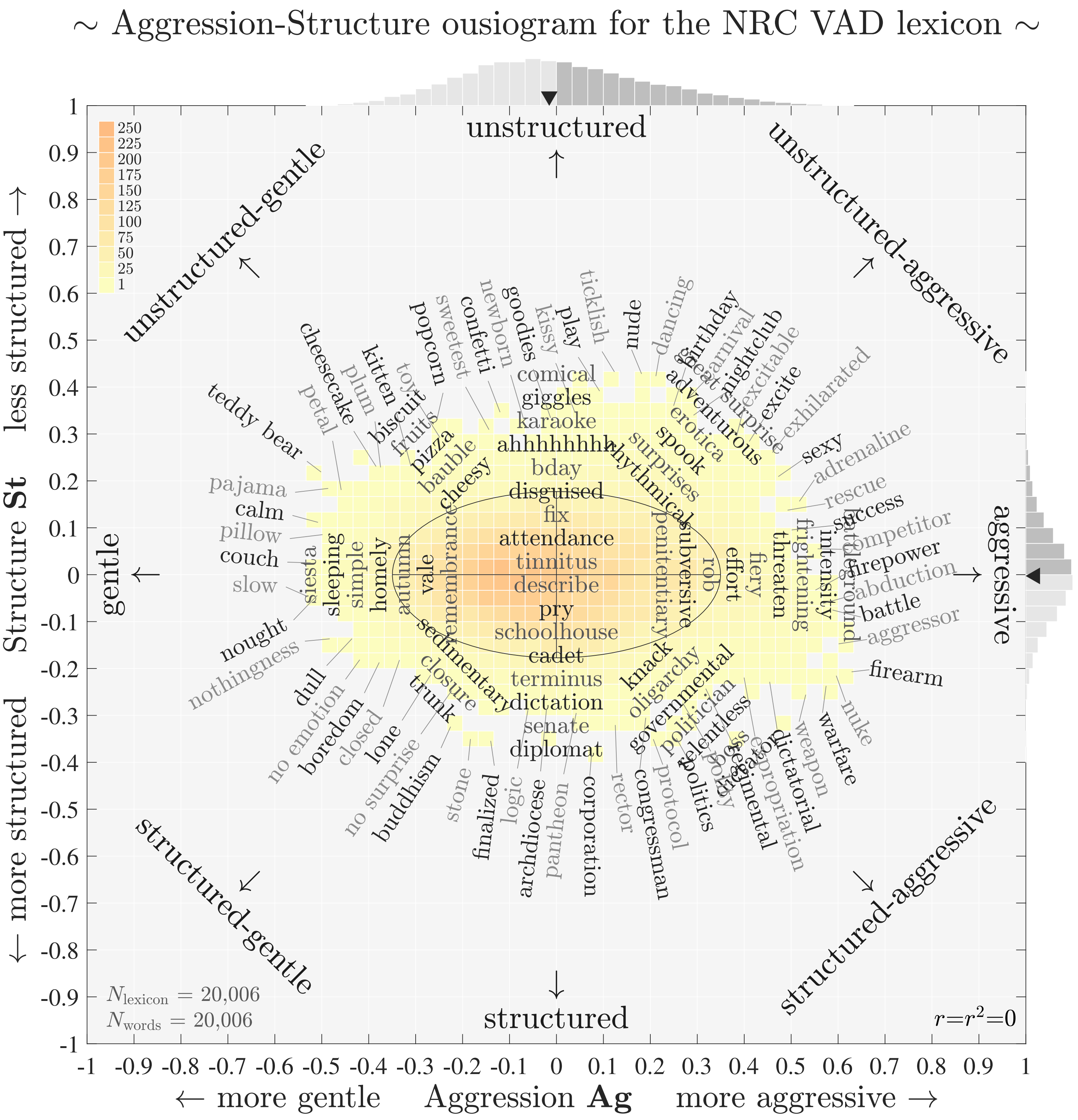

Figure S6: Ousiogram for structure vs. aggression in the GAS framework.

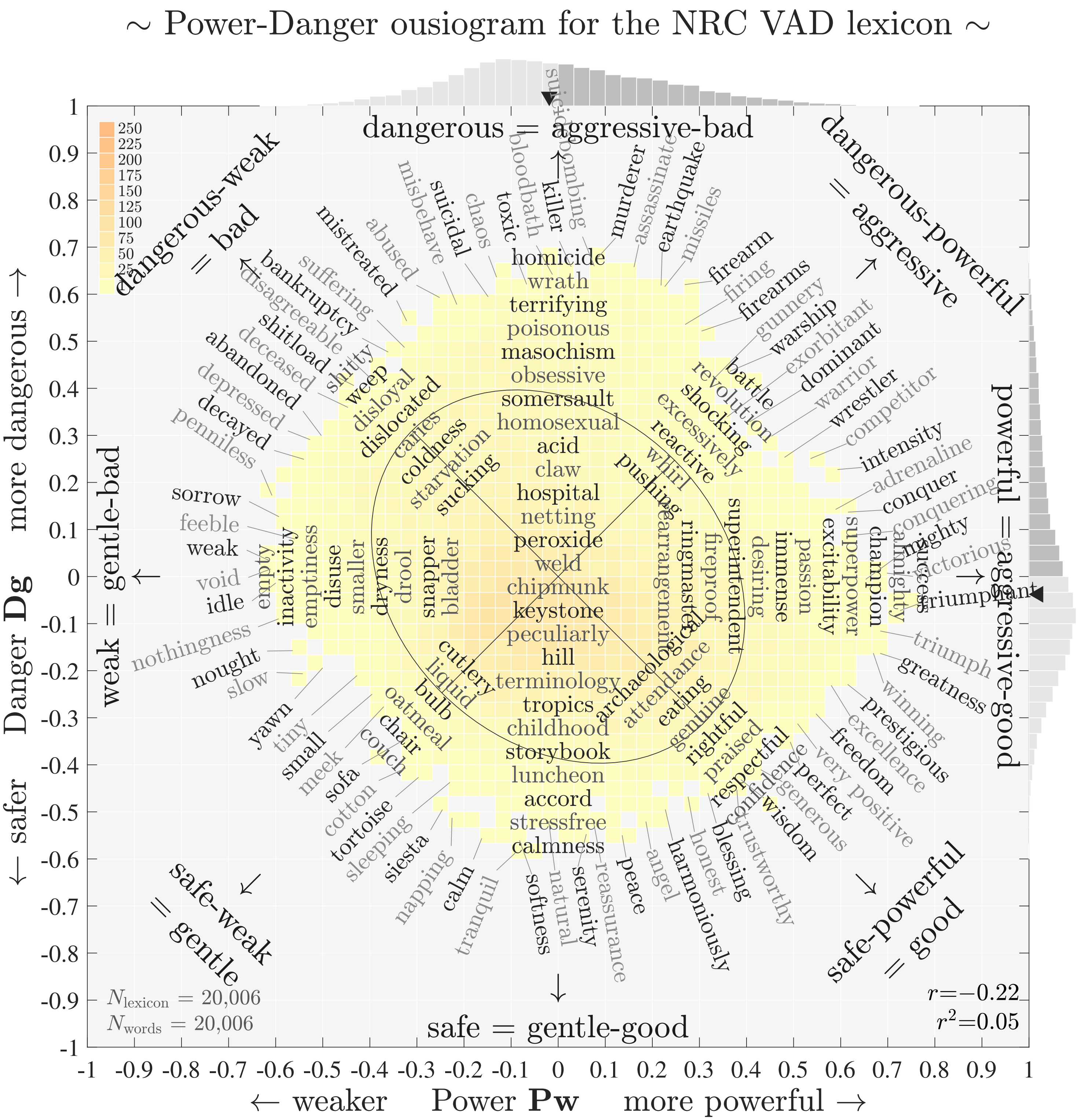

Figure S7: Ousiogram for danger vs. power in the PDS framework. Matches Fig. 3 in the main paper.

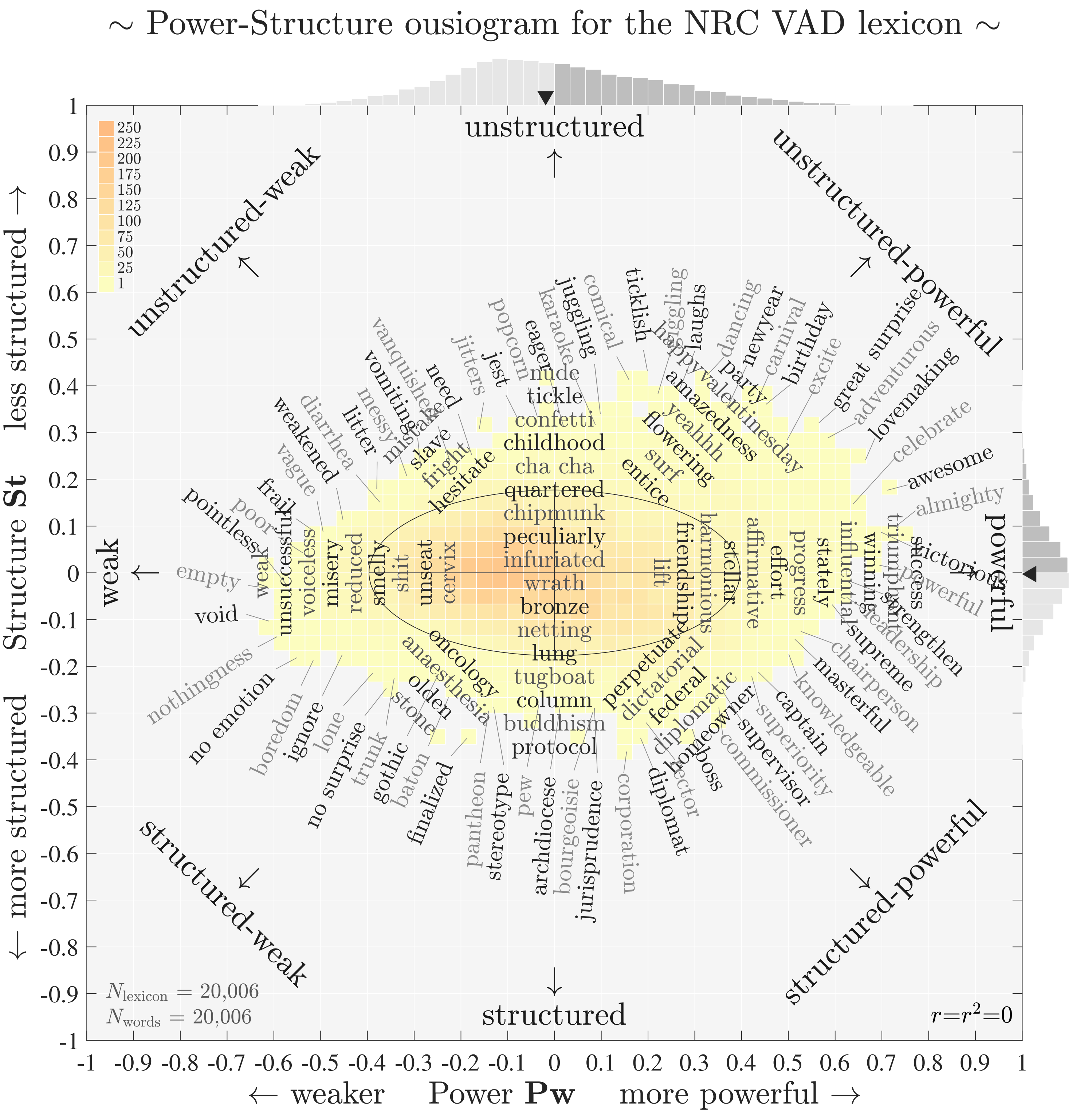

Figure S8: Ousiogram for structure vs. power in the PDS framework.

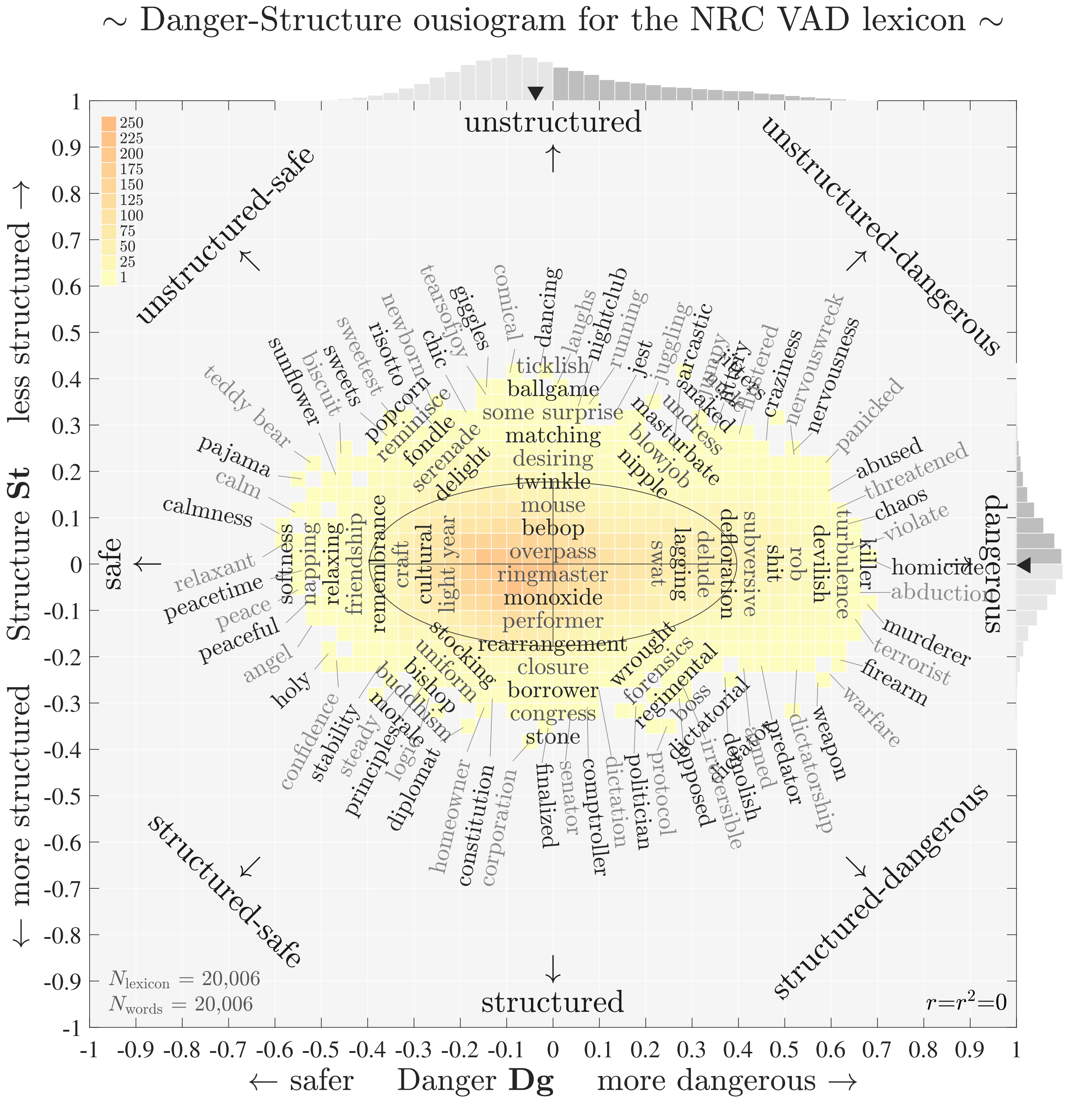

Figure S9: Ousiogram for structure vs. danger in the PDS framework.

S2 Synousionyms and antousionyms, and the problems with prescribing ousiometric axes through end-point descriptors

Which words and terms match in terms of essence of meaning? We define synousionym and antousionym as the ousiometric equivalents of synonym and antonym.

To determine a word's synousionyms, we find the words closest in PDS-space (the specific framework does not matter). For antousionyms, we find words closest to the negated point in PDS-space $(-\mathbf{Pw}, -\mathbf{Dg}, -\mathbf{St})$ (we could equivalently use GAS).

Distilling words to their essential meaning may affect synonym and antonym pairs in opposite ways. Words that are not synonyms may be synousionyms, while words that are antonyms may not be antousionyms.

For example, the word 'failure', $(\mathbf{Pw}, \mathbf{Dg}, \mathbf{St})$ = (-0.39, 0.28, 0.13), is not the antousionym of 'success', $(\mathbf{Pw}, \mathbf{Dg}, \mathbf{St})$ = (0.76, -0.05, 0.09). Within the NRC VAD lexicon, the closest antousionym for 'success' is 'empty', $(\mathbf{Pw}, \mathbf{Dg}, \mathbf{St})$ = (-0.61, -0.01, -0.03). In Tab. S1, we show the closest four synousionyms as well as five antousionyms for the words 'wisdom', 'success', 'volcanic', and 'homicide'. These words are examples of four extreme points of the power-danger ousiogram: safe-powerful, powerful, dangerous-powerful, and dangerous.

In Sec. 3.2, we noted that choosing names of ousiometric dimensions may be problematic, going beyond the issues of end-point descriptors. For one example, the word 'goodness' has the following VAD, GAS, and PDS scores: (0.47, -0.18, 0.21), (0.54, -0.11, -0.02), and (0.30, -0.45, -0.02). We see that 'goodness' has a non-neutral low aggression component and is not purely aligned with the Goodness axis. The five closest synousionyms of 'goodness' are 'thankful', 'friendship', 'motherly', 'hope', and 'graciously' while the five top antousionyms of 'goodness' are 'frustrating', 'cadaver', 'displease', 'shameful', and 'disrespectful'. The antonym 'badness' is not a close antousionym of 'goodness' with VAD, GAS, and PDS scores of (-0.406, 0.323, -0.037), (-0.417, 0.311, 0.008), and (-0.075, 0.515, 0.008). Within the PDS framework, while 'badness' is aligned with the danger axis, 'goodness' is in the safe-powerful quadrant. Some close synousionyms for 'badness' are 'rabid', 'shatter', and 'tremor' and for antousionyms, we find 'comfortable', 'homestead', and 'peacetime'.

A further complication for determining end-point descriptors is that due to the asymmetric, point coverage of essential meaning space, the closest antousionym may not be reflexive. For example, 'chaos' has PDS scores (-0.13, 0.67, 0.09). The closest antousionym for 'chaos' is 'angel' (0.19, -0.52, -0.13) whose closest antousionym is 'shattered' (-0.19, 0.49, 0.11).

These observations again point to the difficulties of prescribing dimensions for participants in surveys. The solution is to see end-point descriptors as guides only and to always examine how participants responded using SVD.

For the PDS framework, 'powerful' and 'dangerous' align well with the end-points of their respective axes with PDS scores of (0.70, -0.02, 0.02) and (0.09, 0.66, 0.10). The word 'weak' similarly aligns well with the negative power axis with PDS scores of (-0.61, 0.03, 0.02). And 'powerful' and 'weak' are both antonyms and close antousionyms of each other.

The descriptor 'safe' does not perform as cleanly however, as it connotes more-than-neutral power with PDS scores of (0.29, -0.41, -0.09). Antousionyms for 'dangerous' are 'relaxed', 'softness', 'calming', 'relaxant', and 'calmness'. (The closest antousionym for 'safe' is 'seasick'.) However semantic differentials also must divide a space into two halves, and {safe ⇔ dangerous} does this well when we consider words above and below the {weak ⇔ powerful} axis. We also feel 'safe' functions well conceptually as an end-point descriptor as it is an easily reached antonym of 'dangerous', if not also an antousionym.

We note that in developing our work, we entertained a number of alternative names for the PDS framework including Success, Stress, and Structure, and Power, Peril, and Play. Ultimately, both of these choices are limited as truly general ousiometric frameworks with success and play in particular eliciting people-centric themes. And in any case, while alliteration may appeal to some, the confusion of variables starting with the same letter would be problematic.

The full space of synousionyms and antousionyms can be explored using VAD, GAS, and PDS scores for all words and terms in the Supplementary Materials.

Safe-Powerful (Good) to Dangerous-Weak (Bad) axis:

Synousionyms	Valence	Arousal	Dominance	Goodness	Aggression	Structure	Power	Danger	Structure
**Anchor: wisdom**	0.430	-0.198	0.371	0.579	-0.031	-0.158	0.388	-0.432	-0.158
education	0.396	-0.225	0.340	0.539	-0.065	-0.167	0.336	-0.427	-0.167
healthy	0.438	-0.181	0.318	0.558	-0.047	-0.108	0.362	-0.428	-0.108
trustworthy	0.469	-0.185	0.324	0.589	-0.052	-0.100	0.379	-0.453	-0.100
reliable	0.412	-0.259	0.375	0.575	-0.076	-0.202	0.353	-0.460	-0.202
Antousionyms	Valence	Arousal	Dominance	Goodness	Aggression	Structure	Power	Danger	Structure
bullshit	-0.458	0.176	-0.317	-0.575	0.046	0.095	-0.373	0.439	0.095
shitty	-0.480	0.179	-0.337	-0.604	0.042	0.100	-0.397	0.456	0.100
nauseate	-0.438	0.160	-0.324	-0.558	0.026	0.101	-0.376	0.413	0.101
weeping	-0.418	0.188	-0.332	-0.549	0.042	0.131	-0.359	0.418	0.131
shame	-0.440	0.170	-0.345	-0.572	0.023	0.120	-0.388	0.421	0.120
diarrhea	-0.408	0.184	-0.357	-0.552	0.023	0.151	-0.374	0.407	0.151

Powerful (Aggressive-Good) to Weak (Gentle-Bad) axis:

Synousionyms	Valence	Arousal	Dominance	Goodness	Aggression	Structure	Power	Danger	Structure
**Anchor: success**	0.459	0.380	0.481	0.571	0.501	0.095	0.758	-0.050	0.095
almighty	0.438	0.374	0.458	0.543	0.487	0.098	0.728	-0.040	0.098
triumphant	0.449	0.337	0.472	0.565	0.462	0.073	0.726	-0.072	0.073
champion	0.390	0.380	0.445	0.494	0.492	0.087	0.698	-0.001	0.087
victorious	0.384	0.386	0.446	0.489	0.499	0.087	0.698	0.007	0.087
Antousionyms	Valence	Arousal	Dominance	Goodness	Aggression	Structure	Power	Danger	Structure
sorrow	-0.448	-0.265	-0.336	-0.509	-0.329	-0.127	-0.593	0.127	-0.127
tasteless	-0.354	-0.304	-0.352	-0.430	-0.385	-0.092	-0.576	0.032	-0.092
idle	-0.321	-0.333	-0.388	-0.414	-0.434	-0.068	-0.600	-0.014	-0.068
empty	-0.312	-0.317	-0.419	-0.424	-0.439	-0.033	-0.610	-0.011	-0.033
void	-0.365	-0.337	-0.370	-0.443	-0.420	-0.103	-0.611	0.016	-0.103

Dangerous-Powerful (Aggressive) to Safe-Weak (Gentle) axis:

Synousionyms	Valence	Arousal	Dominance	Goodness	Aggression	Structure	Power	Danger	Structure
**Anchor: volcanic**	-0.156	0.410	0.281	-0.061	0.515	-0.045	0.322	0.407	-0.045
shelling	-0.163	0.417	0.273	-0.072	0.518	-0.039	0.316	0.417	-0.039
artillery	-0.150	0.412	0.294	-0.050	0.523	-0.050	0.335	0.405	-0.050
wild	-0.188	0.422	0.250	-0.105	0.514	-0.032	0.289	0.438	-0.032
rifles	-0.163	0.364	0.265	-0.068	0.470	-0.062	0.284	0.380	-0.062
Antousionyms	Valence	Arousal	Dominance	Goodness	Aggression	Structure	Power	Danger	Structure
couch	0.094	-0.418	-0.302	-0.002	-0.524	0.025	-0.372	-0.369	0.025
mellow	0.133	-0.431	-0.235	0.066	-0.504	-0.009	-0.310	-0.403	-0.009
pillow	0.163	-0.372	-0.305	0.049	-0.498	0.085	-0.317	-0.387	0.085
tortoise	0.173	-0.422	-0.250	0.092	-0.511	0.025	-0.297	-0.427	0.025
quilt	0.143	-0.377	-0.274	0.048	-0.482	0.052	-0.307	-0.375	0.052
cotton	0.139	-0.429	-0.260	0.059	-0.517	0.012	-0.324	-0.407	0.012

Dangerous (Aggressive-Bad) to Safe (Gentle-Good) axis:

Synousionyms	Valence	Arousal	Dominance	Goodness	Aggression	Structure	Power	Danger	Structure
**Anchor: homicide**	-0.490	0.473	0.018	-0.485	0.478	0.011	-0.005	0.681	0.011
killer	-0.459	0.471	0.043	-0.446	0.485	0.008	0.028	0.658	0.008
psychopath	-0.460	0.443	0.036	-0.446	0.458	-0.003	0.009	0.640	-0.003
bloodshed	-0.452	0.442	0.025	-0.444	0.450	0.008	0.004	0.633	0.008
violate	-0.439	0.470	0.019	-0.440	0.468	0.033	0.020	0.642	0.033
Antousionyms	Valence	Arousal	Dominance	Goodness	Aggression	Structure	Power	Danger	Structure
natural	0.354	-0.382	-0.019	0.354	-0.382	-0.026	-0.020	-0.520	-0.026
tranquil	0.417	-0.406	-0.145	0.351	-0.480	0.078	-0.091	-0.588	0.078
softness	0.375	-0.414	-0.098	0.338	-0.455	0.021	-0.082	-0.561	0.021
serenity	0.400	-0.378	0.057	0.429	-0.345	-0.054	0.060	-0.547	-0.054
comfortable	0.427	-0.337	-0.027	0.406	-0.361	0.039	0.032	-0.542	0.039
calmness	0.434	-0.395	-0.106	0.383	-0.453	0.065	-0.049	-0.591	0.065

Table S1: Example synousionyms and antousionyms for the four axes of the GAS and PDS frameworks. For four anchor words 'wisdom', 'success', 'volcanic', and 'homicide', we list their four closest synousionyms and five antousionyms. For all words, we record scores in the three frameworks of VAD, GAS, and PDS. See the linear transformations of Eq. (2) and Eq. (4) for how VAD connects with GAS and PDS.

S3 Flipbook ‘MRIs’ in power-danger-structure framework

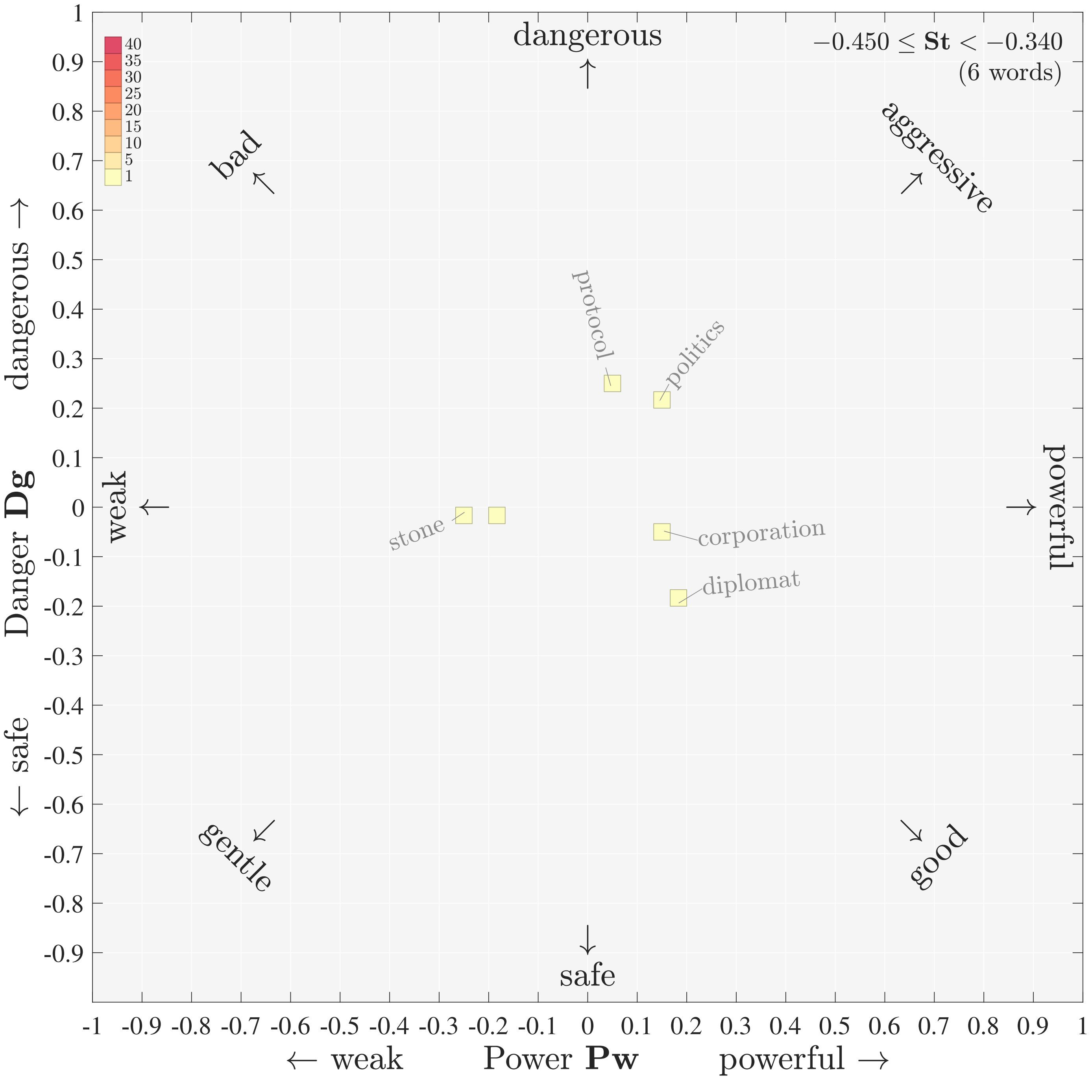

Figure S10: Ousiometric slice for power-danger plane with structure: $-0.450 \leq \textbf{St} < -0.340$.

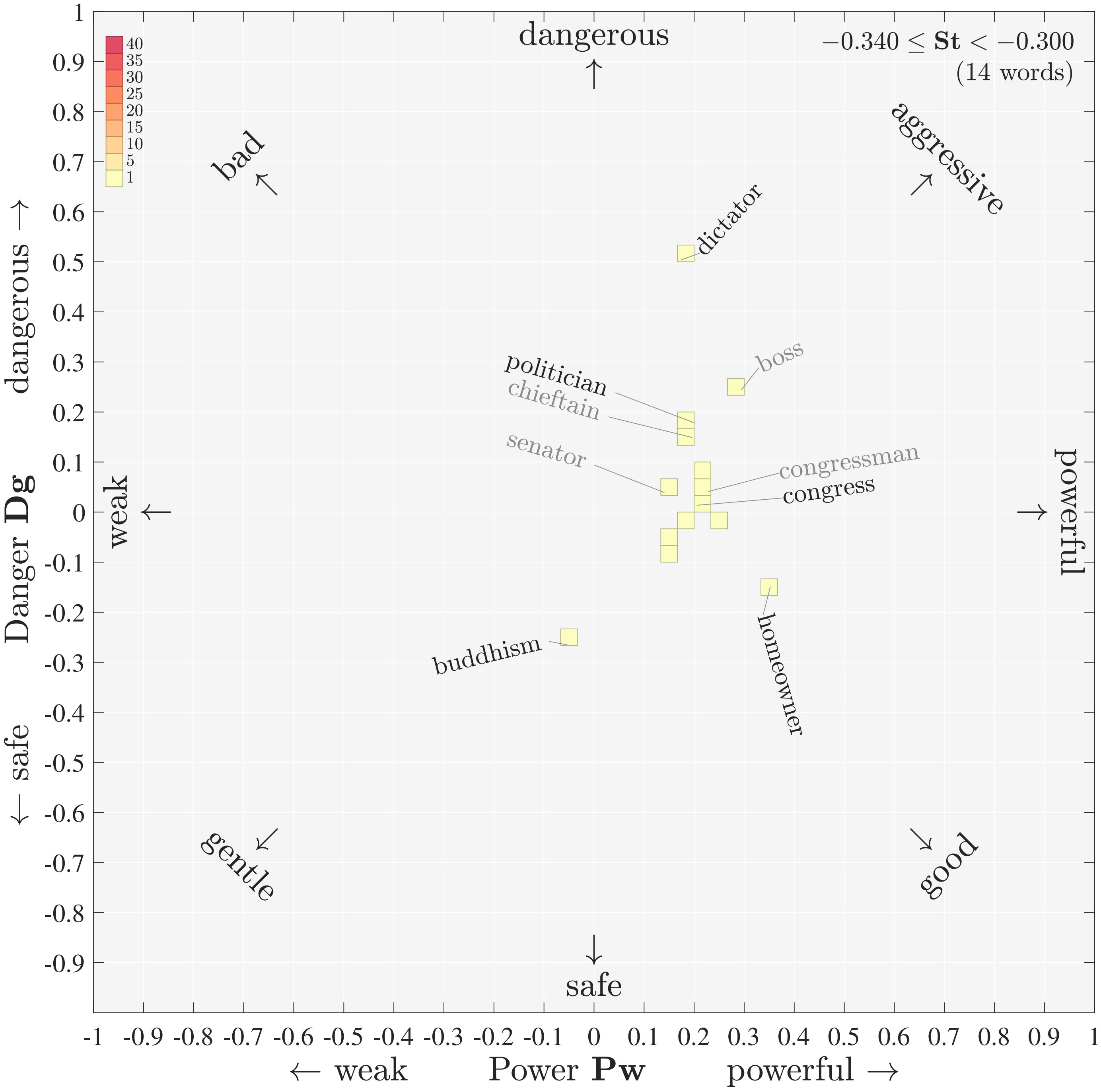

Figure S11: Ousiometric slice for power-danger plane with structure: $-0.340 \leq \mathbf{St} < -0.300$.

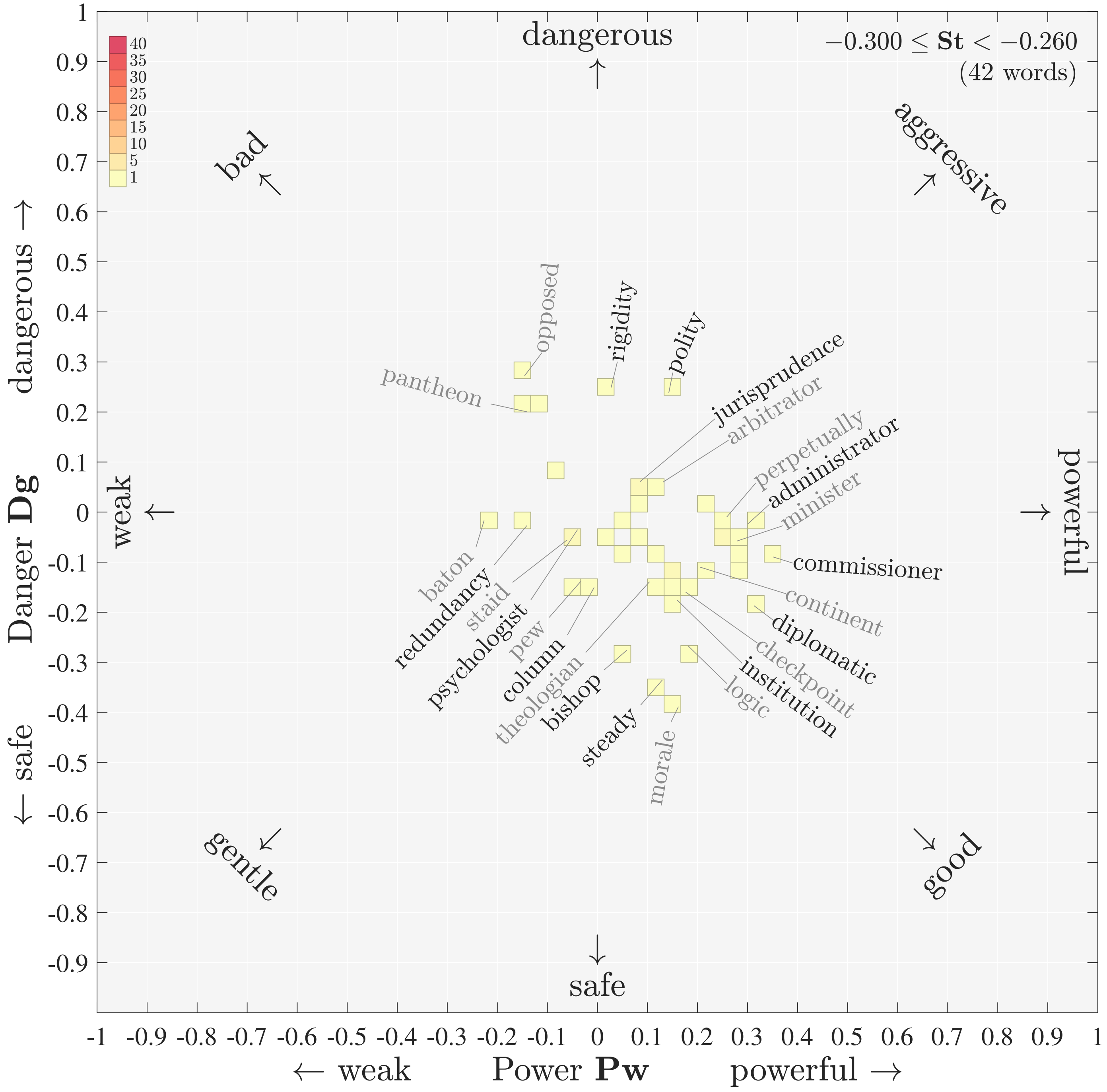

Figure S12: Ousiometric slice for power-danger plane with structure: $-0.300 \leq \mathbf{St} < -0.260$.

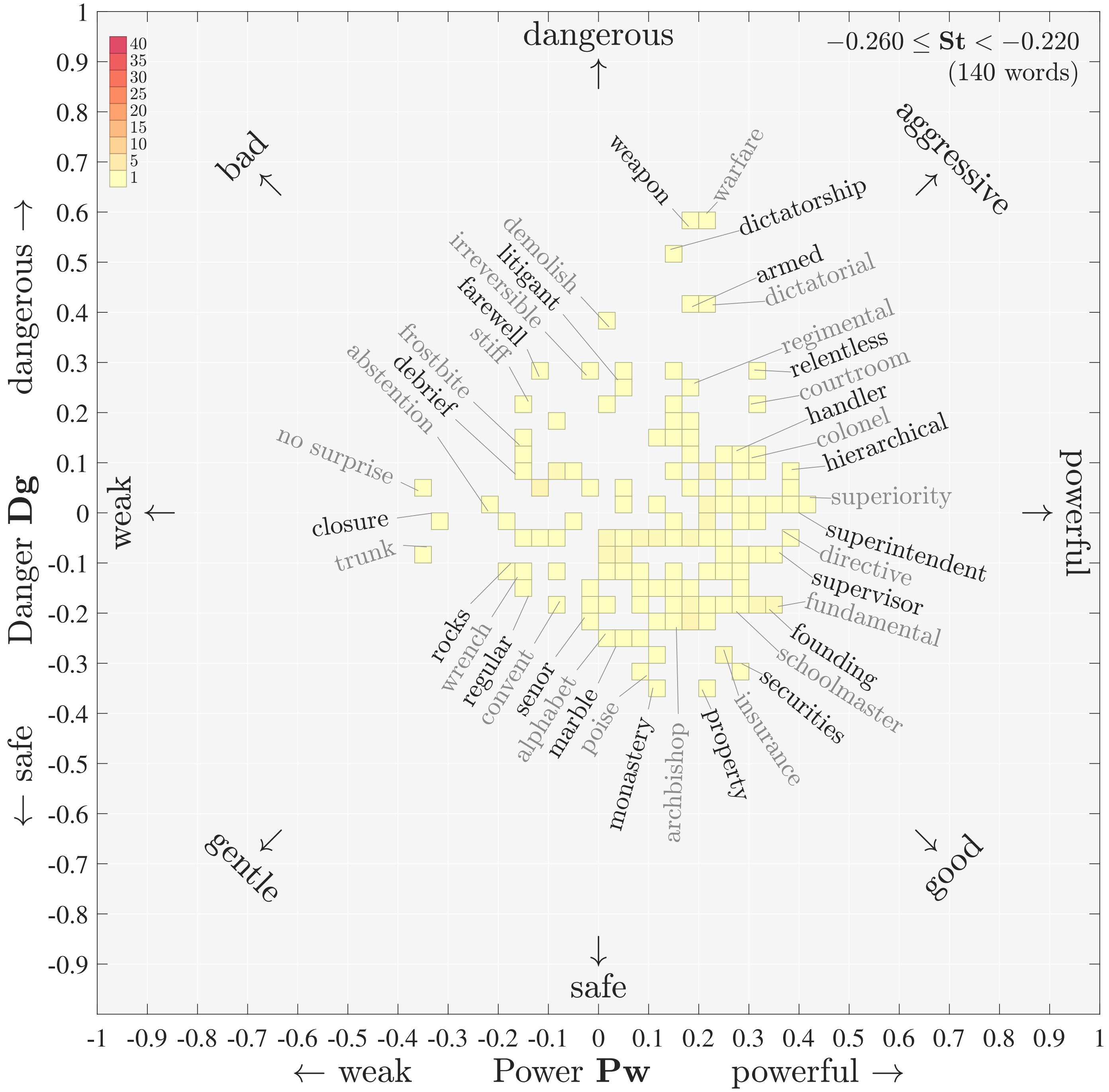

Figure S13: Ousiometric slice for power-danger plane with structure: $-0.260 \leq$ St < -0.220.

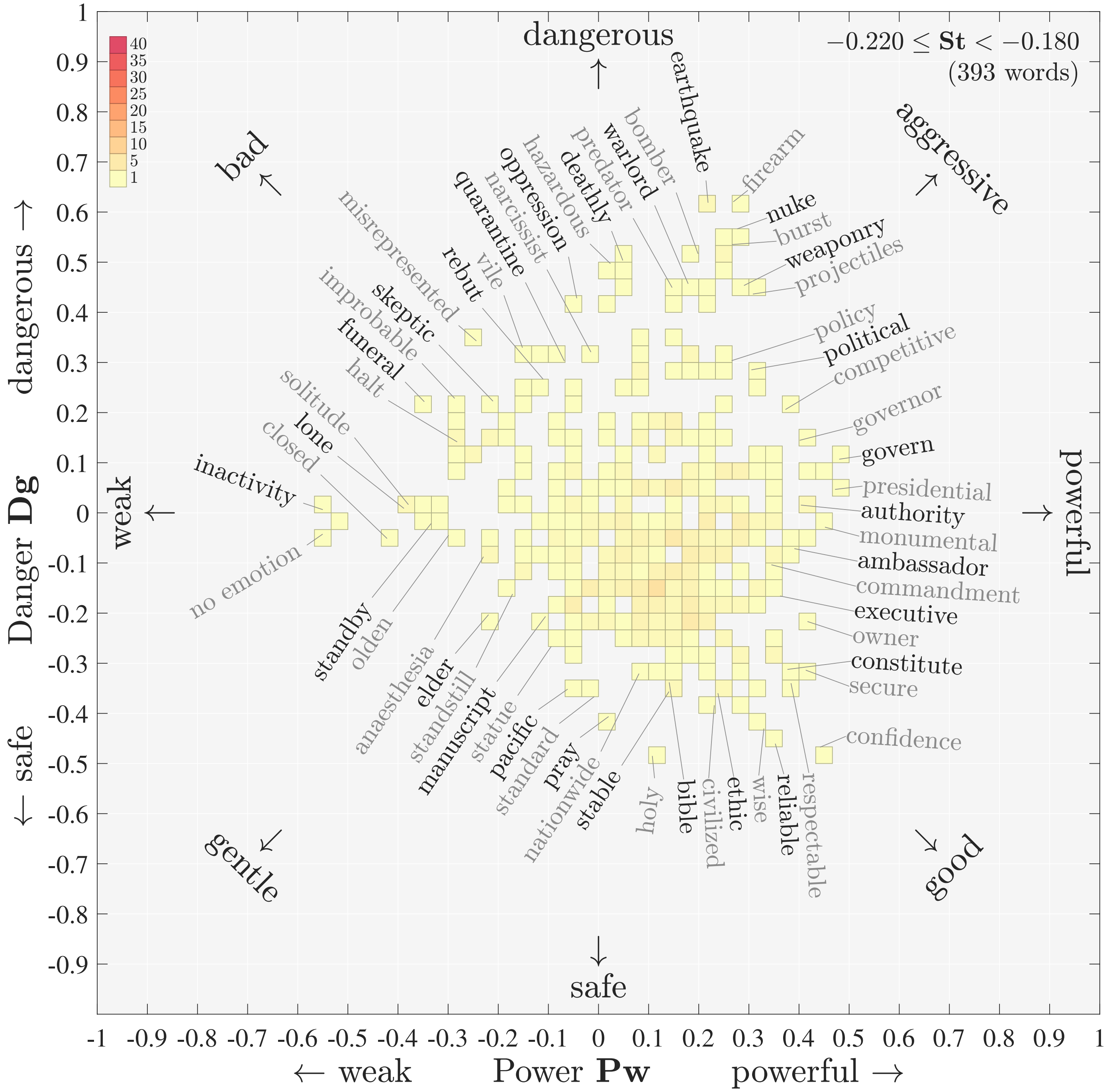

Figure S14: Ousiometric slice for power-danger plane with structure: $-0.220 \leq \mathbf{St} < -0.180$.

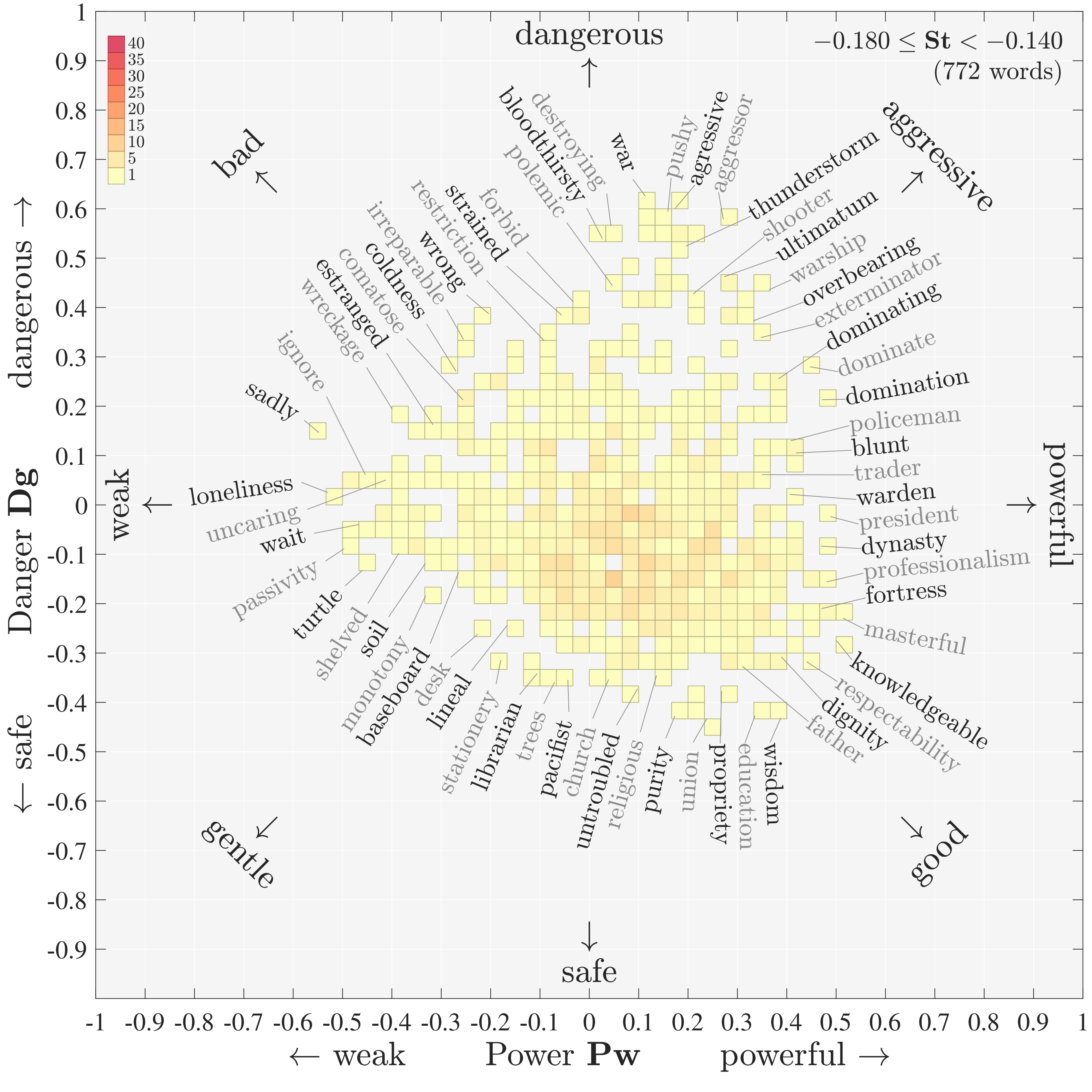

Figure S15: Ousiometric slice for power-danger plane with structure: $-0.180 \leq \mathbf{St} < -0.140$.

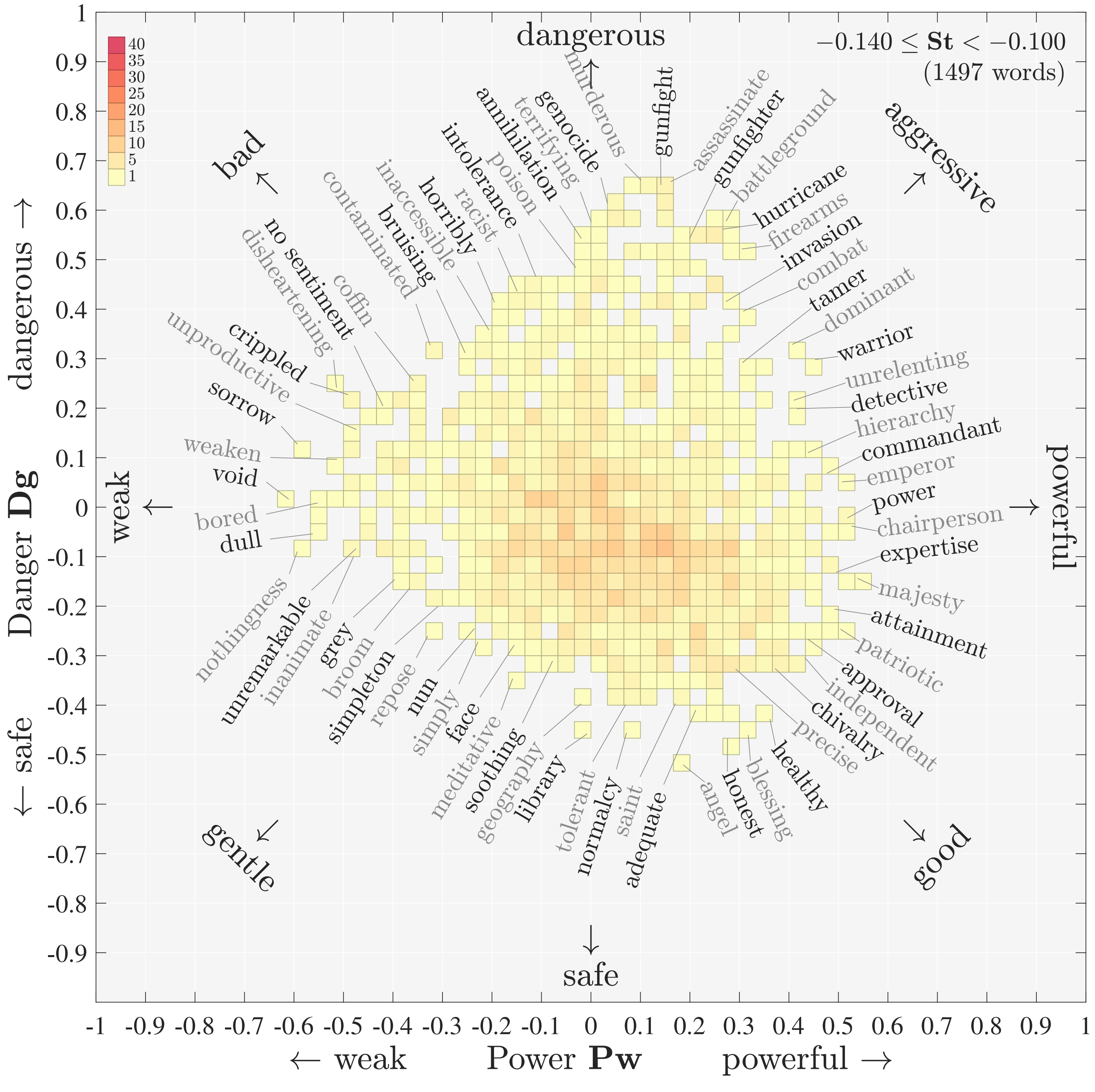

Figure S16: Ousiometric slice for power-danger plane with structure: $-0.140 \leq \textbf{St} < -0.100$.

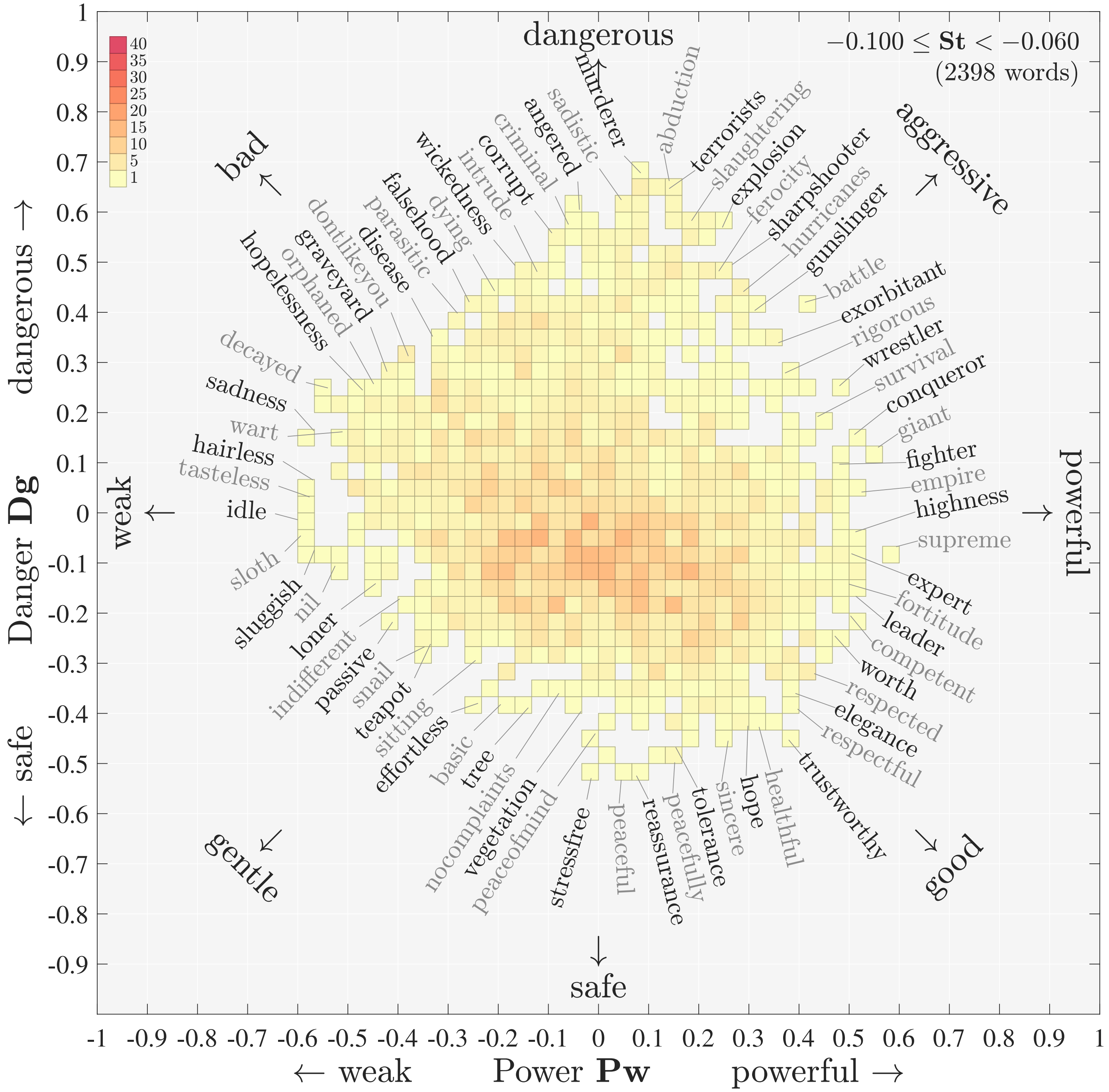

Figure S17: Ousiometric slice for power-danger plane with structure: $-0.100 \leq \mathbf{St} < -0.060$.

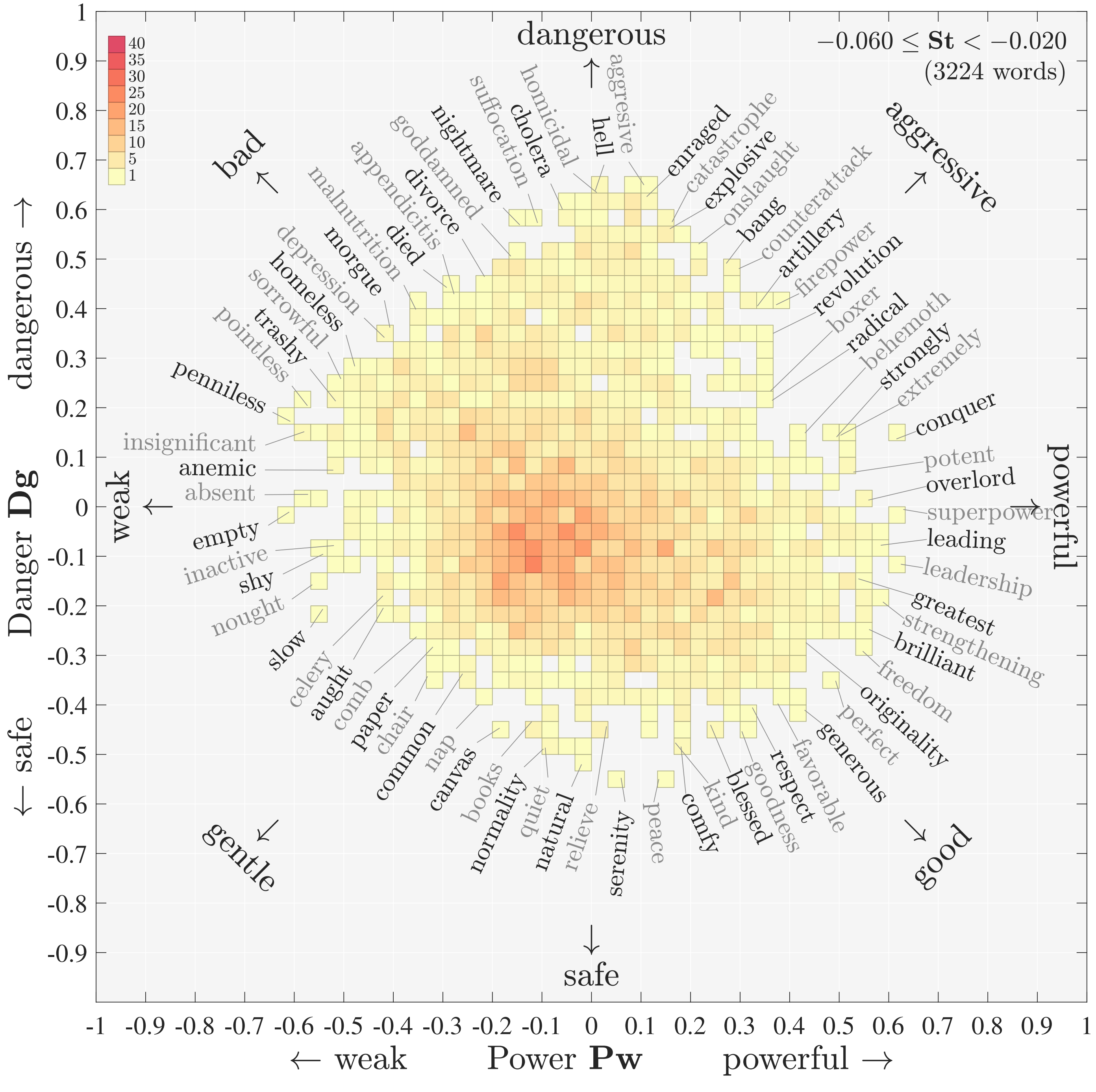

Figure S18: **Ousiometric slice for power-danger plane with structure:** $-0.060 \leq \mathbf{St} < -0.020$.

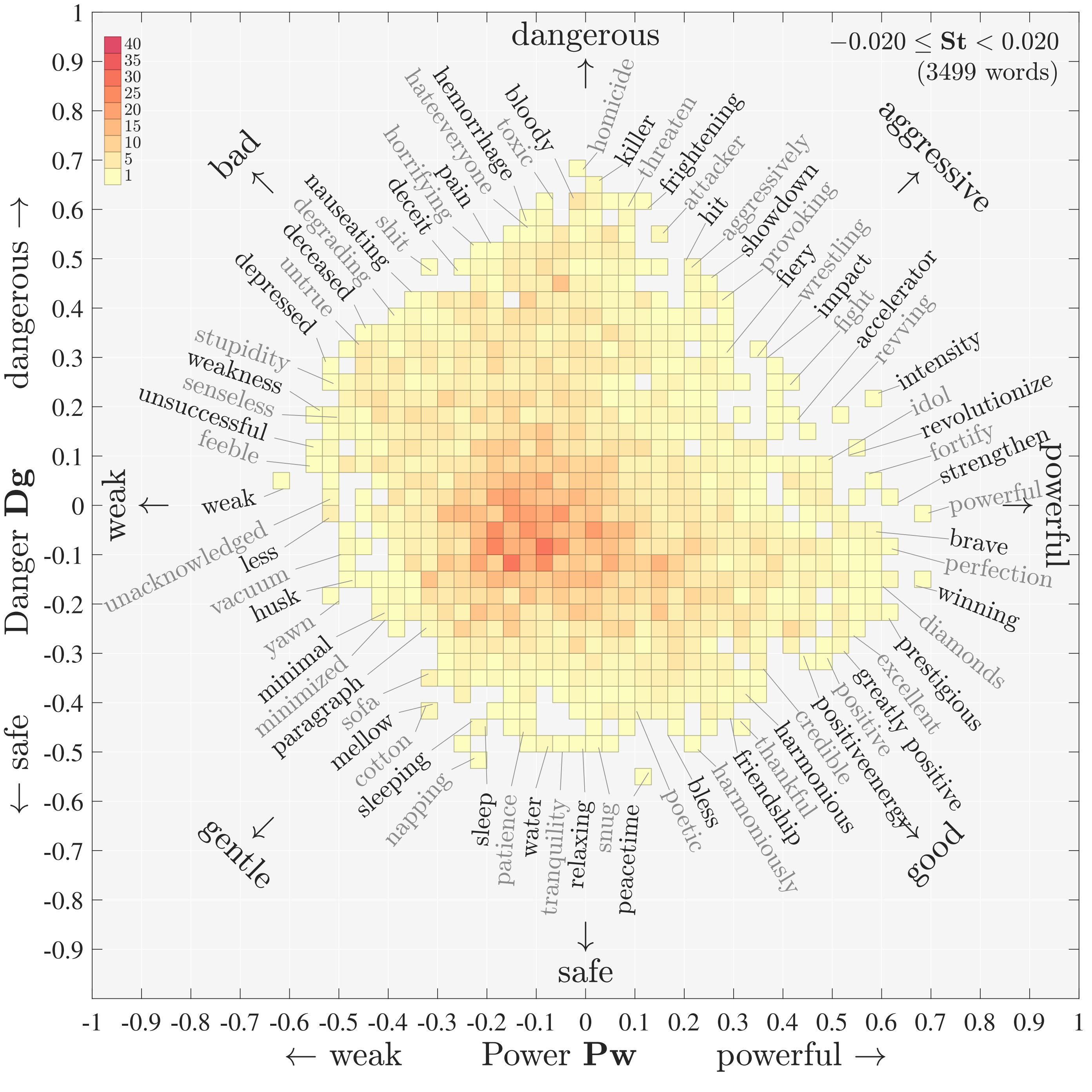

Figure S19: Ousiometric slice for power-danger plane with structure: $-0.020 \leq \textbf{St} < 0.020$.

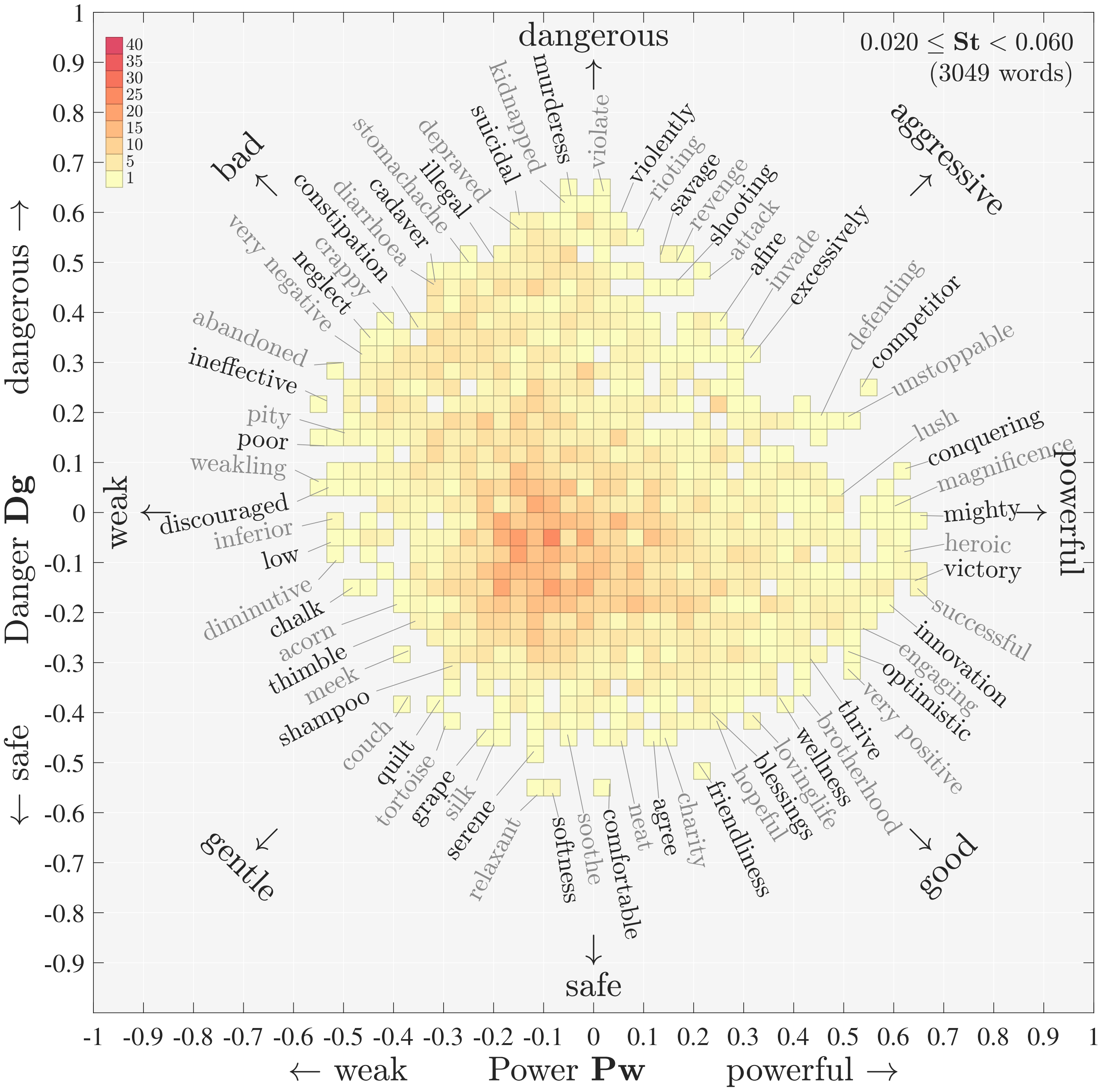

Figure S20: Ousiometric slice for power-danger plane with structure: $0.020 \leq \mathbf{St} < 0.060$.

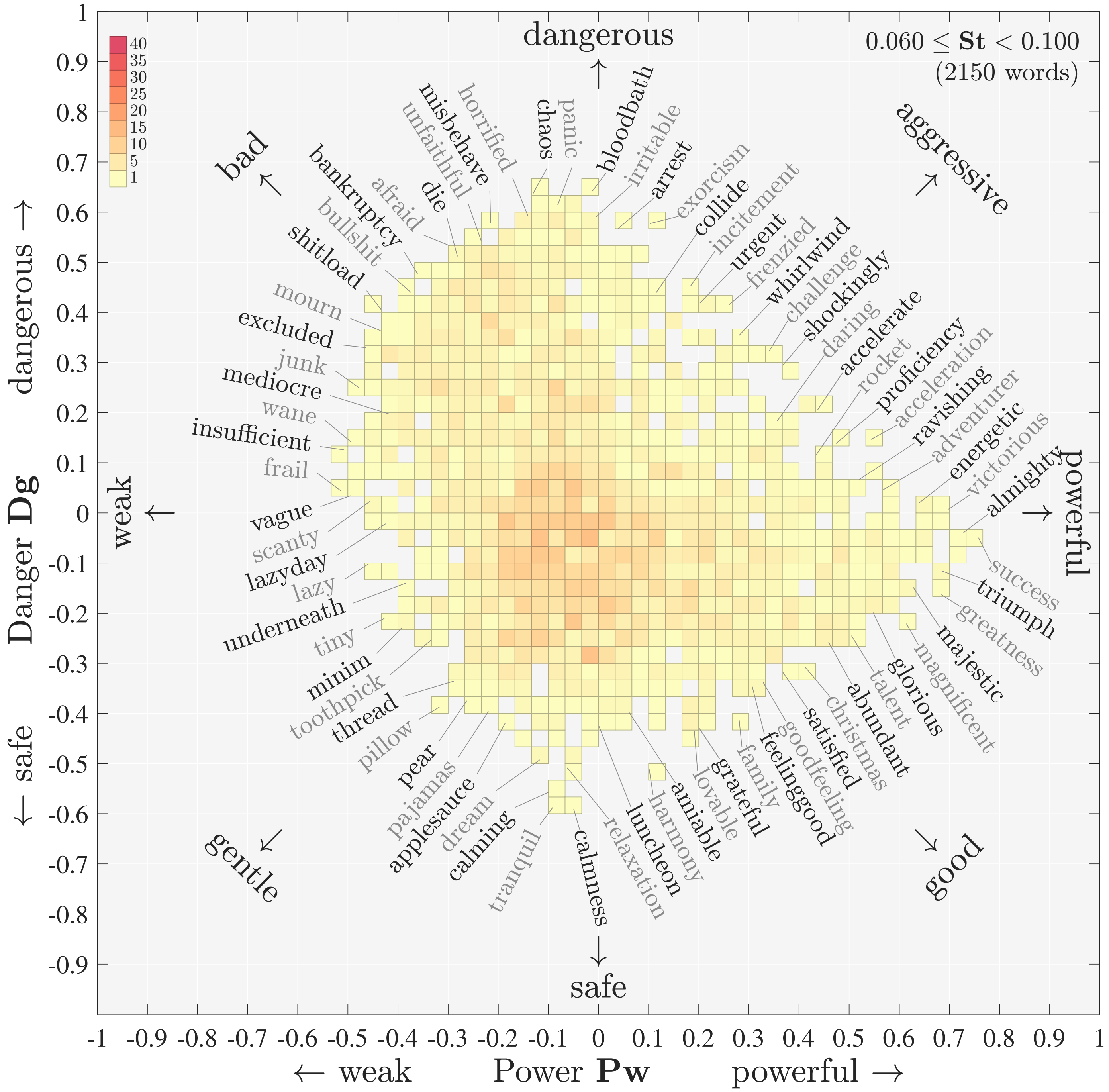

Figure S21: Ousiometric slice for power-danger plane with structure: $0.060 \leq \mathbf{St} < 0.100$.

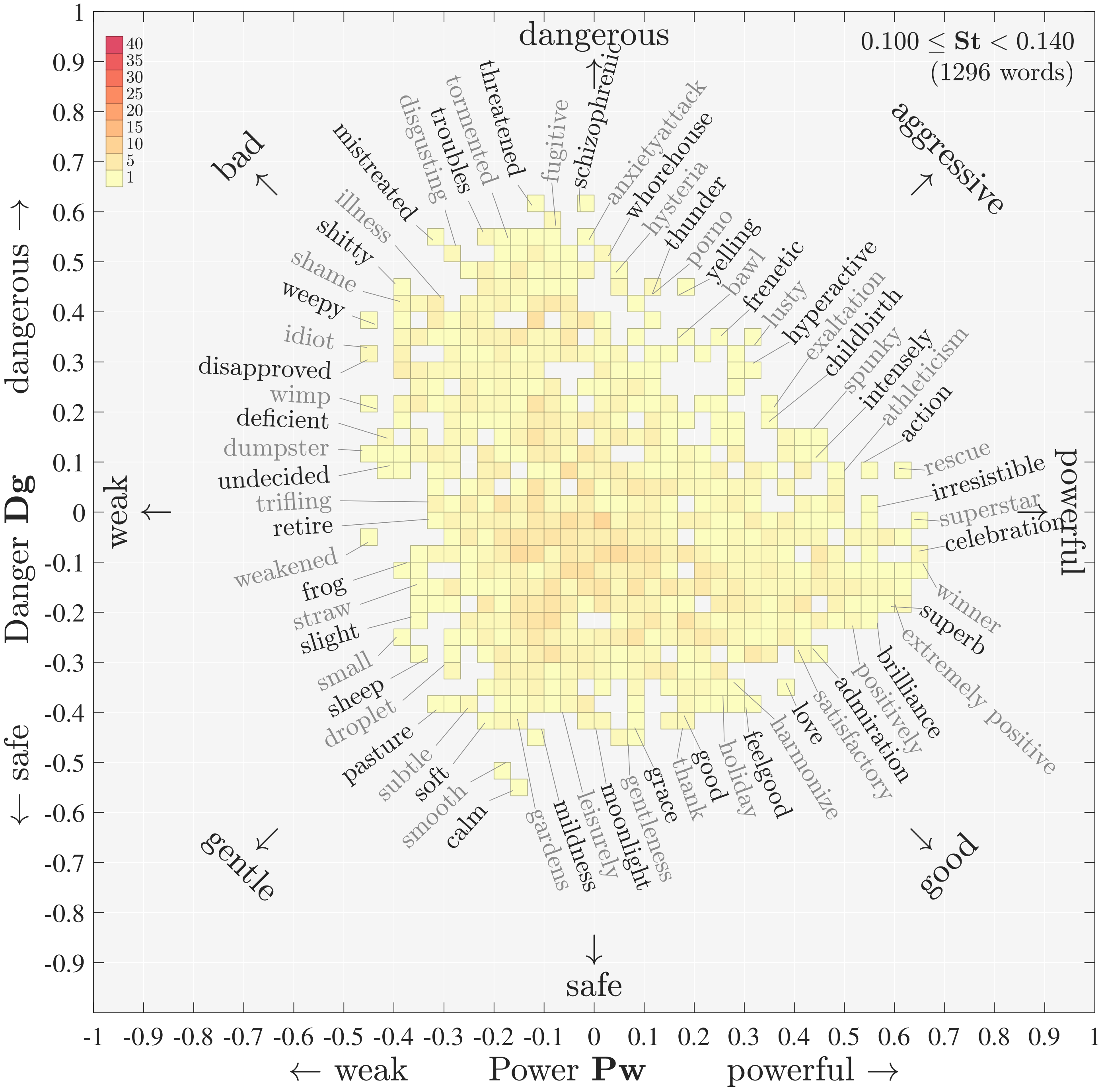

Figure S22: Ousiometric slice for power-danger plane with structure: $0.100 \leq \textbf{St} < 0.140$.

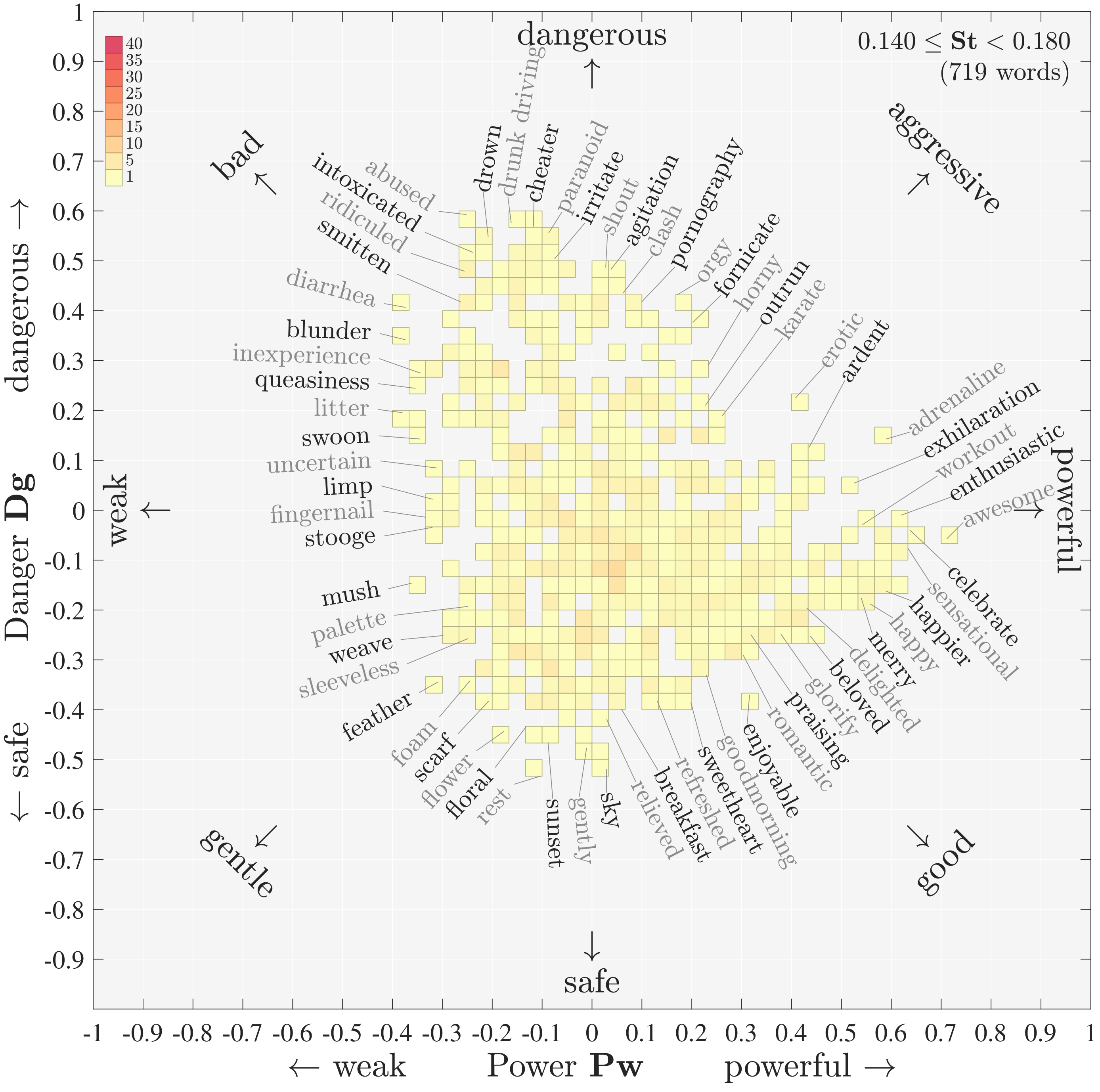

Figure S23: Ousiometric slice for power-danger plane with structure: $0.140 \leq \textbf{St} < 0.180$.

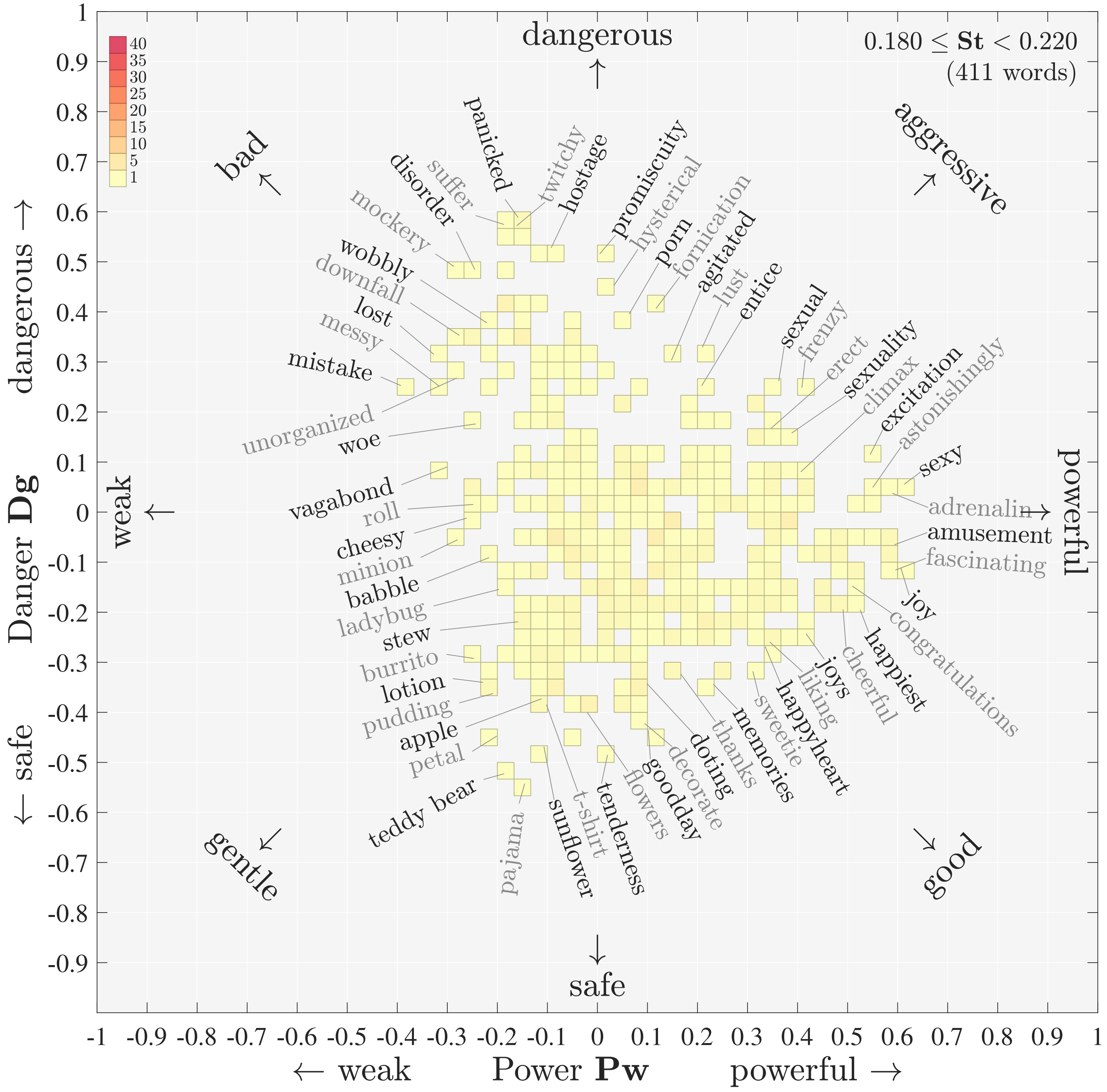

Figure S24: Ousiometric slice for power-danger plane with structure: $0.180 \leq \mathbf{St} < 0.220$.

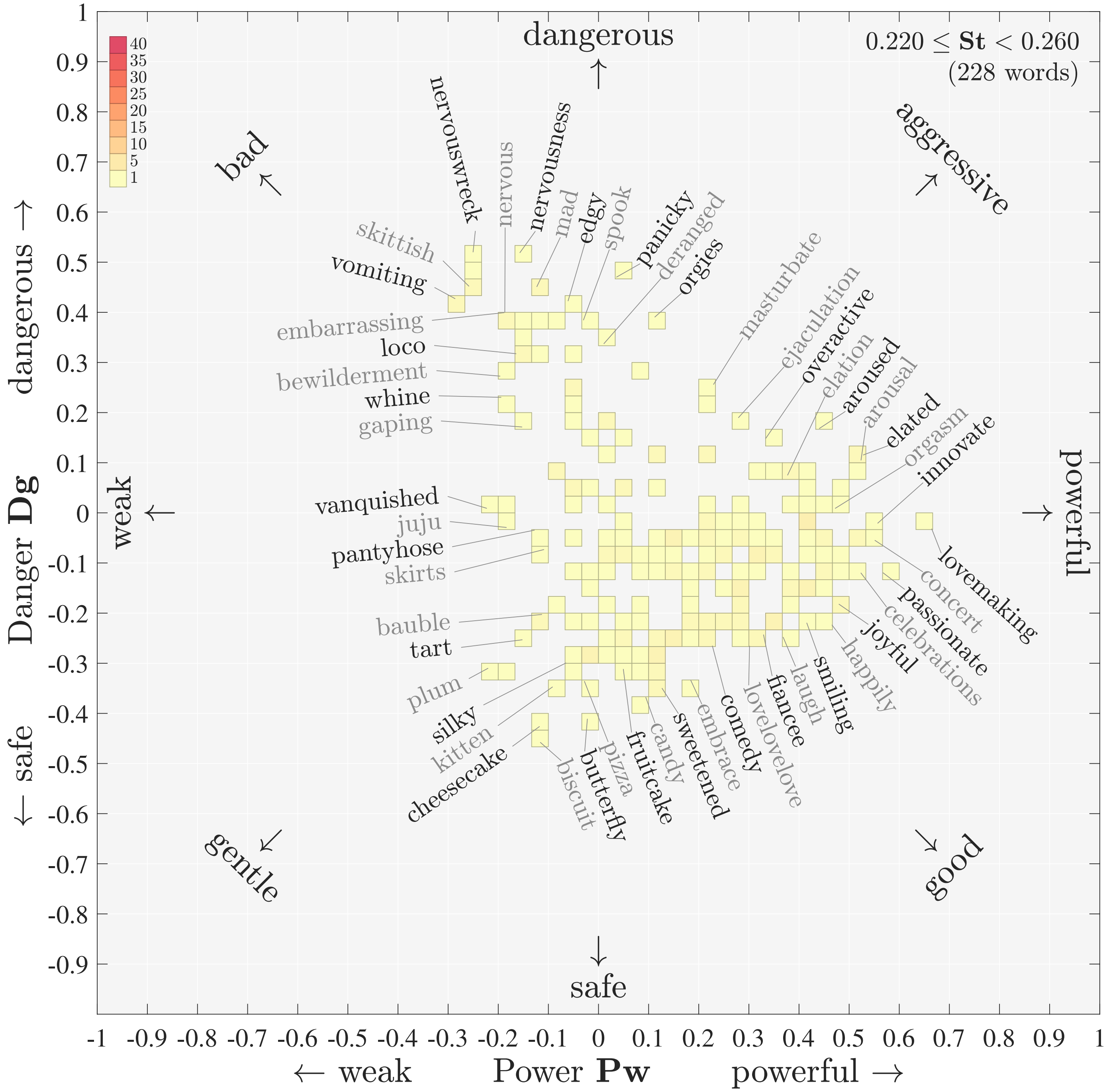

Figure S25: Ousiometric slice for power-danger plane with structure: $0.220 \leq \mathbf{St} < 0.260$.

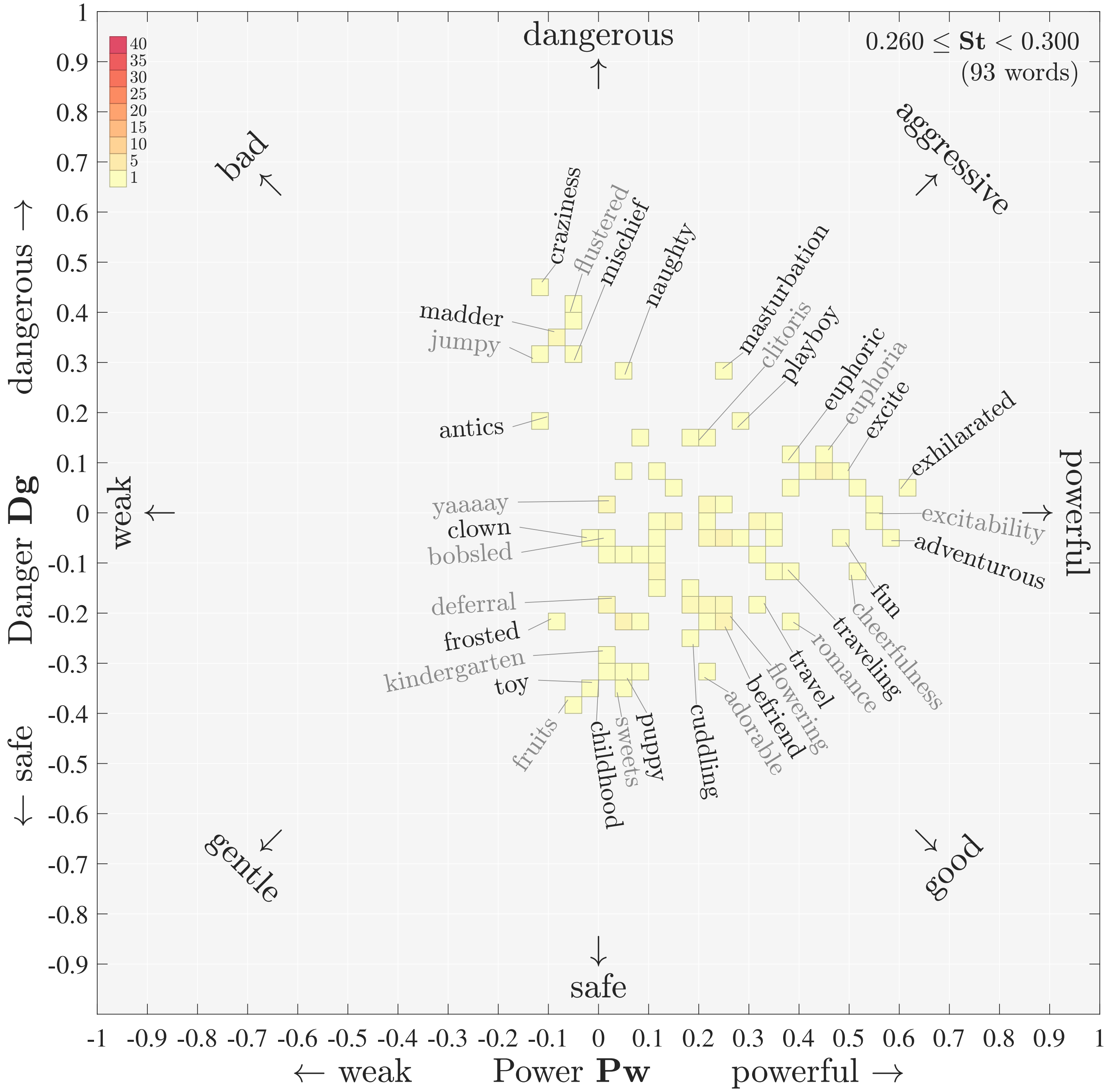

Figure S26: Ousiometric slice for power-danger plane with structure: $0.260 \leq \mathbf{St} < 0.300$.

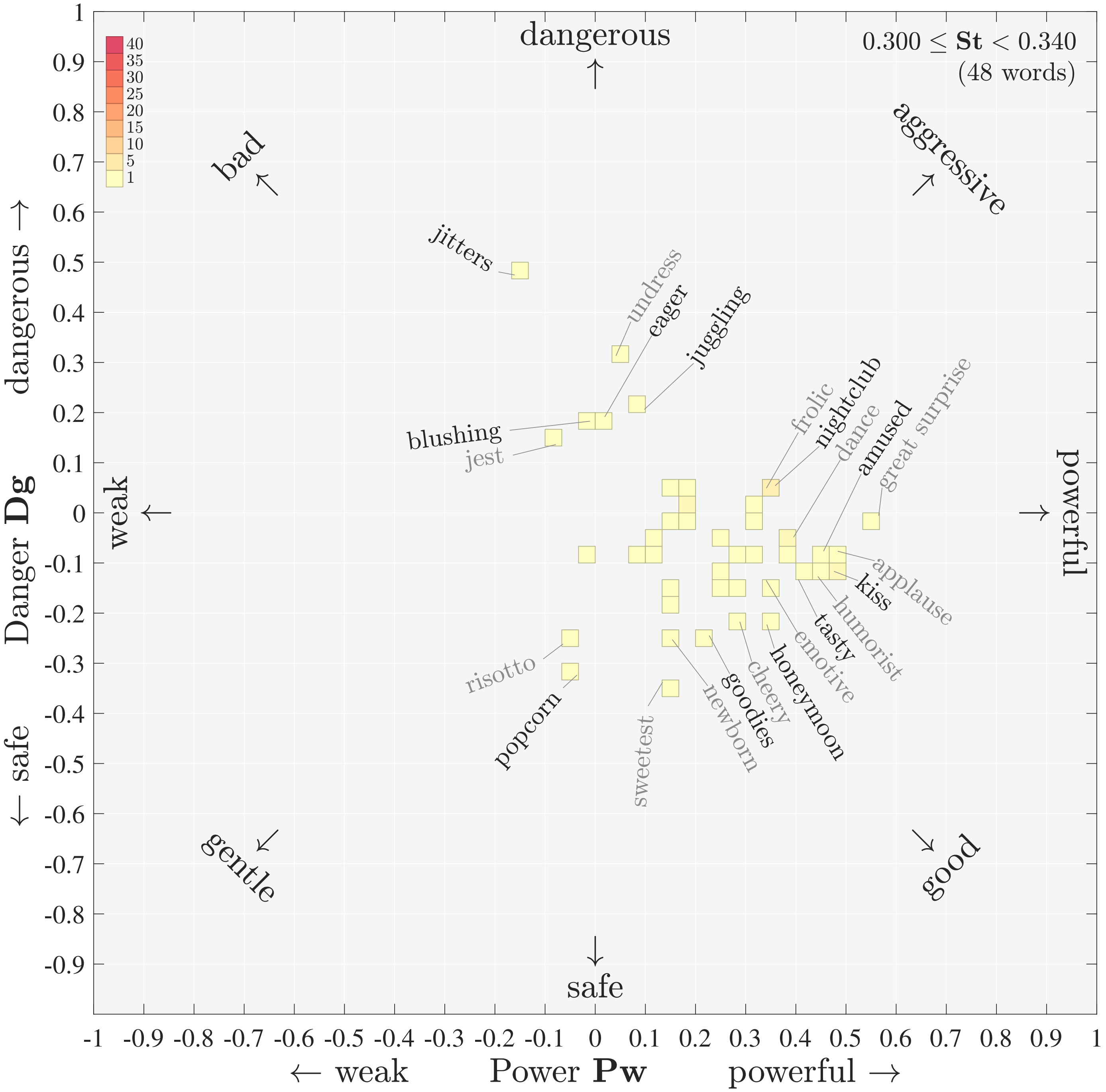

Figure S27: Ousiometric slice for power-danger plane with structure: $0.300 \leq \mathbf{St} < 0.340$.

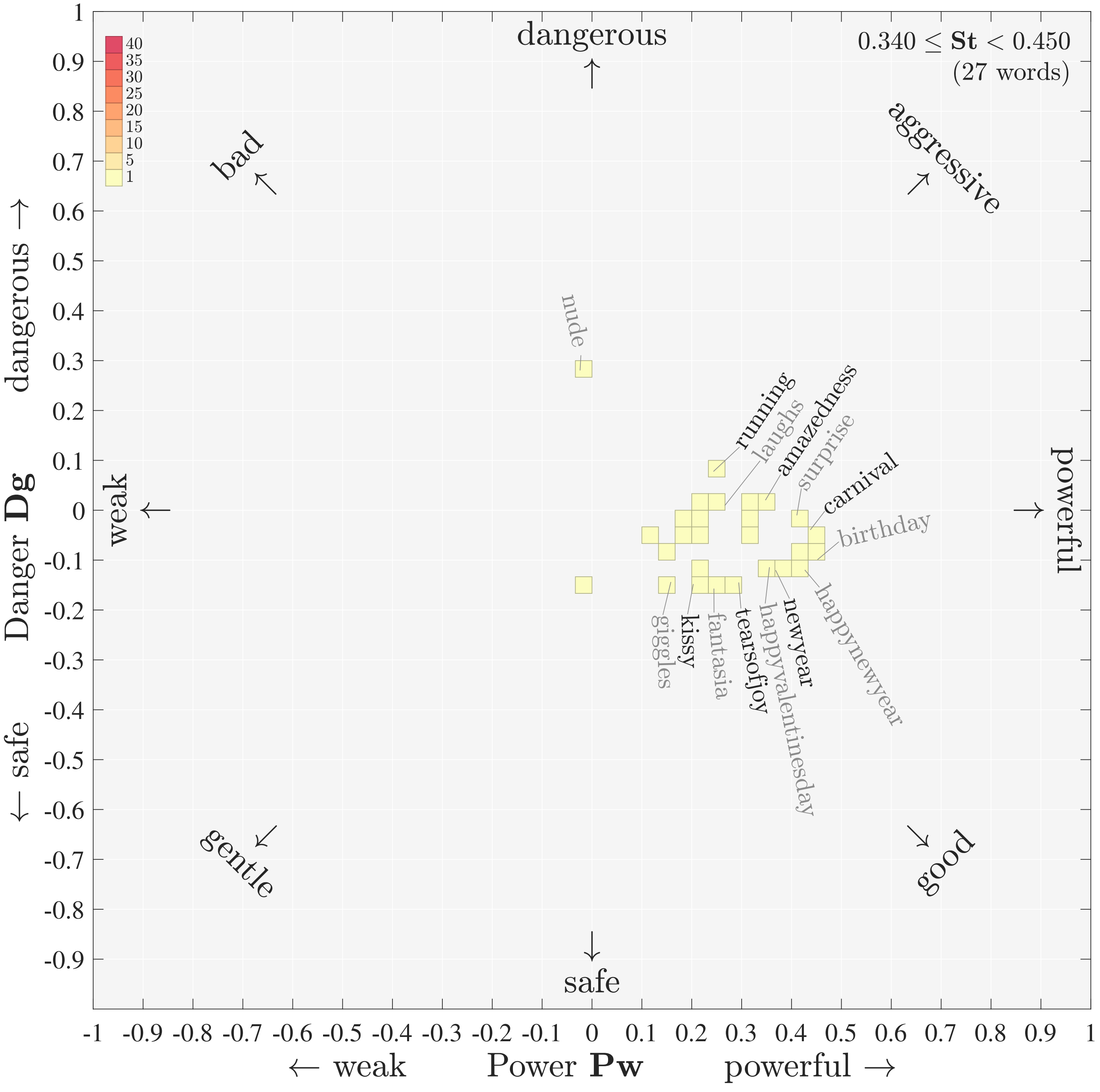

Figure S28: Ousiometric slice for power-danger plane with structure: $0.340 \leq \mathbf{St} < 0.450$.

S4 Tables for the 13 semantic differential pairs within cube-based ousiometric framework

safe ⇐ 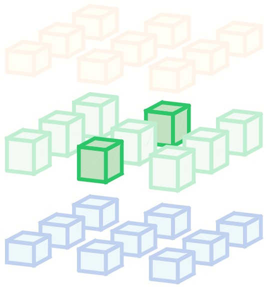 ⇒ **dangerous**

(gentle-good)
peaceful
serene
angelic

(aggressive-bad)
murderous
violent
demonic

Word	Comp. Size	Size	Angle	Cos.	Pw	Dg	St
1. **calmness**	77.1	77.9	7.9	0.99	-0.05	-0.59	0.07
2. **tranquil**	76.7	78.3	11.6	0.98	-0.09	-0.59	0.08
3. **relaxant**	73.7	75.5	12.5	0.98	-0.11	-0.56	0.05
4. **softness**	73.2	74.1	8.6	0.99	-0.08	-0.56	0.02
5. **calming**	72.7	74.3	12.0	0.98	-0.10	-0.56	0.07
6. **relaxed**	72.0	73.3	10.8	0.98	-0.10	-0.55	0.03
7. **peace**	71.8	74.3	14.9	0.97	0.14	-0.55	-0.06
8. **serenity**	71.5	72.3	8.4	0.99	0.06	-0.55	-0.05
9. **comfortable**	70.8	71.1	5.3	1.00	0.03	-0.54	0.04
10. **peacetime**	70.8	72.7	13.3	0.97	0.13	-0.54	-0.01
11. **peaceful**	69.6	70.7	10.3	0.98	0.05	-0.53	-0.09
12. **stressfree**	69.1	70.1	9.7	0.99	-0.01	-0.53	-0.09
13. **reassurance**	68.5	70.1	12.5	0.98	0.08	-0.52	-0.09
14. **natural**	67.9	68.1	3.6	1.00	-0.02	-0.52	-0.03
15. **sky**	67.8	70.4	15.6	0.96	0.03	-0.52	0.14
16. **harmony**	66.7	68.4	13.0	0.97	0.10	-0.51	0.06
17. **relaxation**	66.4	67.6	10.9	0.98	-0.06	-0.51	0.08
18. **tranquility**	65.3	65.6	5.4	1.00	-0.05	-0.50	0.00
19. **relaxing**	64.4	64.5	0.7	1.00	-0.01	-0.49	-0.00
20. **teatime**	64.4	66.9	15.9	0.96	-0.11	-0.49	0.09

Word	Comp. Size	Size	Angle	Cos.	Pw	Dg	St
1. **homicide**	89.0	89.0	179.0	-1.00	-0.01	0.68	0.01
2. **murderer**	88.5	89.9	169.9	-0.98	0.08	0.68	-0.09
3. **abduction**	86.5	88.8	166.9	-0.97	0.14	0.66	-0.06
4. **murderous**	86.3	88.6	166.8	-0.97	0.10	0.66	-0.12
5. **suicidebombing**	86.2	87.1	171.5	-0.99	0.08	0.66	-0.06
6. **killer**	86.0	86.1	177.5	-1.00	0.03	0.66	0.01
7. **dangerous**	85.9	87.6	168.8	-0.98	0.09	0.66	-0.10
8. **assassinate**	85.9	89.6	163.4	-0.96	0.16	0.66	-0.11
9. **terrorist**	85.1	87.4	166.8	-0.97	0.10	0.65	-0.12
10. **gunfight**	85.0	88.0	165.1	-0.97	0.14	0.65	-0.10
11. **gunshot**	85.0	87.1	167.4	-0.98	0.11	0.65	-0.09
12. **terrorism**	84.9	87.2	167.0	-0.97	0.13	0.65	-0.07
13. **aggresive**	84.8	86.2	169.8	-0.98	0.11	0.65	-0.05
14. **terrorists**	84.5	87.1	165.7	-0.97	0.14	0.65	-0.08
15. **tsunami**	84.3	86.7	166.4	-0.97	0.12	0.65	-0.10
16. **violate**	83.9	84.0	176.6	-1.00	0.02	0.64	0.03
17. **bloodbath**	83.8	84.2	174.2	-0.99	-0.01	0.64	0.06
18. **slaughter**	83.7	84.3	173.2	-0.99	0.07	0.64	-0.04
19. **psychopath**	83.5	83.5	179.2	-1.00	0.01	0.64	-0.00
20. **hell**	83.3	83.6	175.8	-1.00	0.01	0.64	-0.05

Figure S29: Words with largest components in safe and dangerous directions, within a cone of half angle $\frac{1}{2}\frac{180}{\pi}\cos^{-1}(2/\sqrt{6}) \simeq 17.6°$.

gentle ⇐ 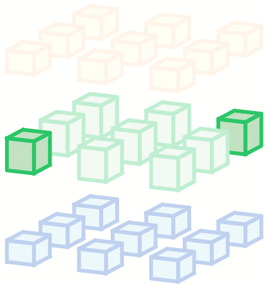 ⇒ **aggressive**

(safe-weak)
restful
meek
sleepy

(dangerous-powerful)
competitive
warlike
forceful

Word	Comp. Size	Size	Angle	Cos.	Pw	Dg	St
1. **couch**	68.4	68.5	2.8	1.00	-0.37	-0.37	0.03
2. **cotton**	67.5	68.0	6.6	0.99	-0.32	-0.41	0.01
3. **tortoise**	66.8	67.9	10.6	0.98	-0.30	-0.43	0.03
4. **mellow**	65.9	66.4	7.5	0.99	-0.31	-0.40	-0.01
5. **pasture**	65.5	67.9	15.2	0.97	-0.31	-0.39	0.12
6. **pillow**	65.1	66.3	11.2	0.98	-0.32	-0.39	0.09
7. **lawn**	63.1	65.3	15.0	0.97	-0.30	-0.39	0.11
8. **quilt**	63.0	63.7	8.3	0.99	-0.31	-0.37	0.05
9. **chair**	62.2	62.5	5.3	1.00	-0.33	-0.34	-0.04
10. **sleeping**	61.6	64.5	17.3	0.95	-0.23	-0.44	0.00
11. **asleep**	61.6	61.8	4.8	1.00	-0.31	-0.36	-0.01
12. **sofa**	61.0	61.0	2.5	1.00	-0.32	-0.34	-0.01
13. **sleepy**	60.3	60.4	3.8	1.00	-0.31	-0.34	0.02
14. **meek**	59.7	60.5	9.2	0.99	-0.37	-0.28	0.03
15. **subtle**	59.5	62.4	17.6	0.95	-0.25	-0.39	0.11
16. **minimized**	58.9	61.1	15.4	0.96	-0.41	-0.23	-0.02
17. **pear**	58.9	60.6	13.7	0.97	-0.26	-0.37	0.08
18. **napkin**	58.5	60.4	14.3	0.97	-0.40	-0.24	-0.01
19. **teacup**	57.9	58.5	8.3	0.99	-0.27	-0.36	0.02
20. **sheep**	57.9	60.2	16.0	0.96	-0.33	-0.29	0.12

Word	Comp. Size	Size	Angle	Cos.	Pw	Dg	St
1. **battle**	76.8	77.4	172.7	-0.99	0.41	0.42	-0.08
2. **warship**	73.9	76.9	163.9	-0.96	0.36	0.44	-0.15
3. **firepower**	72.1	72.3	175.7	-1.00	0.37	0.41	-0.03
4. **counterattack**	71.8	74.0	166.1	-0.97	0.30	0.48	-0.04
5. **bang**	70.5	73.4	163.7	-0.96	0.27	0.49	-0.03
6. **warrior**	69.4	72.2	163.9	-0.96	0.45	0.30	-0.11
7. **artillery**	68.3	68.9	172.4	-0.99	0.33	0.41	-0.05
8. **dominant**	67.9	70.1	165.5	-0.97	0.41	0.33	-0.12
9. **tornado**	67.8	70.9	162.8	-0.96	0.28	0.46	-0.09
10. **shelling**	67.6	68.5	171.1	-0.99	0.32	0.42	-0.04
11. **hurricanes**	67.4	69.7	165.4	-0.97	0.29	0.44	-0.08
12. **volcanic**	67.3	68.0	171.7	-0.99	0.32	0.41	-0.04
13. **wild**	67.1	68.6	167.9	-0.98	0.29	0.44	-0.03
14. **showdown**	66.2	68.9	163.8	-0.96	0.26	0.46	0.02
15. **revolution**	66.1	66.2	176.8	-1.00	0.37	0.35	-0.03
16. **gunslinger**	66.0	67.8	166.8	-0.97	0.31	0.40	-0.10
17. **fighting**	65.8	68.1	165.4	-0.97	0.29	0.43	-0.08
18. **explode**	65.4	68.2	163.8	-0.96	0.27	0.44	-0.08
19. **overbearing**	65.1	68.2	162.8	-0.96	0.33	0.37	-0.15
20. **combat**	65.0	67.8	163.3	-0.96	0.31	0.40	-0.14

Figure S30: Words with largest components in gentle and aggressive directions, within a cone of half angle $\frac{1}{2}\frac{180}{\pi}\cos^{-1}(2/\sqrt{6}) \simeq 17.6°$.

weak ⇐ 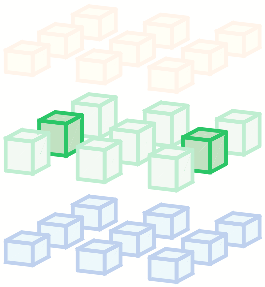 ⇒ powerful

(gentle-bad)
empty
idle
foolish

(aggressive-good)
triumphant
mighty
heroic

Word	Comp. Size	Size	Angle	Cos.	Pw	Dg	St
1. **weak**	80.0	80.2	3.6	1.00	-0.61	0.03	0.02
2. **void**	79.7	80.9	9.7	0.99	-0.61	0.02	-0.10
3. **empty**	79.7	79.8	3.3	1.00	-0.61	-0.01	-0.03
4. **penniless**	79.4	82.6	15.9	0.96	-0.61	0.17	-0.02
5. **idle**	78.3	78.9	6.6	0.99	-0.60	-0.01	-0.07
6. **sloth**	77.6	78.5	9.0	0.99	-0.59	-0.05	-0.08
7. **nothingness**	77.5	80.4	15.4	0.96	-0.59	-0.09	-0.14
8. **sorrow**	77.4	80.9	16.9	0.96	-0.59	0.13	-0.13
9. **insignificant**	75.7	78.4	15.1	0.97	-0.58	0.15	-0.04
10. **hopeless**	75.7	78.0	13.8	0.97	-0.58	0.14	-0.04
11. **tasteless**	75.3	76.3	9.6	0.99	-0.58	0.03	-0.09
12. **absent**	74.7	75.0	4.9	1.00	-0.57	0.03	-0.04
13. **hairless**	74.3	75.4	9.5	0.99	-0.57	0.07	-0.07
14. **sluggish**	74.0	75.8	12.3	0.98	-0.57	-0.07	-0.10
15. **nought**	73.6	76.7	16.2	0.96	-0.56	-0.16	-0.04
16. **dull**	73.4	75.9	14.6	0.97	-0.56	-0.05	-0.14
17. **unhelpful**	73.1	75.6	14.8	0.97	-0.56	0.15	-0.02
18. **boring**	73.0	73.2	4.4	1.00	-0.56	0.01	-0.04
19. **feeble**	73.0	73.8	8.3	0.99	-0.56	0.08	-0.01
20. **bored**	72.1	74.3	14.1	0.97	-0.55	0.01	-0.14

Word	Comp. Size	Size	Angle	Cos.	Pw	Dg	St
1. **success**	99.0	100.0	171.9	-0.99	0.76	-0.05	0.10
2. **almighty**	95.1	96.1	171.7	-0.99	0.73	-0.04	0.10
3. **triumphant**	94.8	95.8	171.9	-0.99	0.73	-0.07	0.07
4. **awesome**	92.9	96.0	165.4	-0.97	0.71	-0.06	0.18
5. **victorious**	91.2	91.9	172.9	-0.99	0.70	0.01	0.09
6. **champion**	91.1	91.8	172.9	-0.99	0.70	-0.00	0.09
7. **powerful**	90.7	90.8	178.1	-1.00	0.69	-0.02	0.02
8. **triumph**	89.3	91.4	167.7	-0.98	0.68	-0.12	0.09
9. **greatness**	89.3	92.2	165.6	-0.97	0.68	-0.16	0.07
10. **superman**	87.8	89.4	169.3	-0.98	0.67	-0.10	0.08
11. **winning**	87.4	90.0	166.3	-0.97	0.67	-0.16	0.01
12. **richness**	86.4	87.2	172.3	-0.99	0.66	-0.04	0.08
13. **breathtaking**	86.4	87.8	169.4	-0.98	0.66	-0.10	0.08
14. **winner**	85.7	88.2	166.3	-0.97	0.66	-0.10	0.12
15. **mighty**	85.3	85.6	175.3	-1.00	0.65	-0.01	0.05
16. **invincible**	85.0	85.7	172.7	-0.99	0.65	-0.00	0.08
17. **celebration**	84.7	86.8	167.2	-0.98	0.65	-0.08	0.12
18. **successful**	84.6	87.0	166.5	-0.97	0.65	-0.15	0.04
19. **victory**	83.9	85.9	167.5	-0.98	0.64	-0.14	0.04
20. **succeed**	83.9	85.4	169.0	-0.98	0.64	-0.11	0.05

Figure S31: Words with largest components in weak and powerful directions, within a cone of half angle $\frac{1}{2}\frac{180}{\pi}\cos^{-1}(2/\sqrt{6}) \simeq 17.6°$.

bad ⇐ 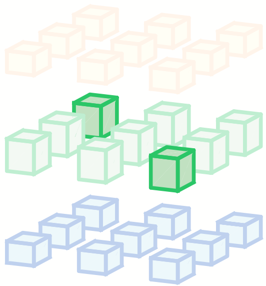 ⇒ **good**

(dangerous-weak)
suffering
failing
sorrowful

(safe-powerful)
virtuous
honorable
uplifting

Word	Comp. Size	Size	Angle	Cos.	Pw	Dg	St
1. **shitty**	78.8	80.1	10.2	0.98	-0.40	0.46	0.10
2. **shitload**	77.5	78.0	6.2	0.99	-0.43	0.41	0.06
3. **bankruptcy**	77.4	78.7	10.5	0.98	-0.36	0.48	0.07
4. **depressed**	75.5	78.6	16.1	0.96	-0.53	0.29	-0.01
5. **weepy**	75.1	77.0	12.6	0.98	-0.44	0.38	0.12
6. **suffering**	75.1	77.1	13.2	0.97	-0.33	0.49	0.07
7. **bullshit**	75.0	76.3	10.5	0.98	-0.37	0.44	0.10
8. **disagreeable**	74.7	75.5	8.2	0.99	-0.39	0.42	0.08
9. **shame**	74.6	76.3	12.1	0.98	-0.39	0.42	0.12
10. **deceased**	74.4	74.8	6.2	0.99	-0.45	0.36	0.01
11. **abandoned**	74.1	76.5	14.5	0.97	-0.50	0.30	0.03
12. **weep**	73.9	74.7	8.5	0.99	-0.41	0.39	0.08
13. **mourn**	73.7	74.6	9.0	0.99	-0.43	0.36	0.07
14. **neglect**	73.5	74.2	8.2	0.99	-0.45	0.35	0.04
15. **excluded**	73.3	74.8	11.6	0.98	-0.46	0.33	0.06
16. **nauseate**	72.9	74.2	10.6	0.98	-0.38	0.41	0.10
17. **shit**	72.8	74.4	11.6	0.98	-0.31	0.48	-0.00
18. **untrue**	72.5	73.6	9.7	0.99	-0.46	0.33	-0.01
19. **nauseating**	72.5	72.9	6.1	0.99	-0.35	0.43	0.02
20. **idiot**	72.3	74.5	13.9	0.97	-0.45	0.33	0.11

Word	Comp. Size	Size	Angle	Cos.	Pw	Dg	St
1. **perfect**	78.0	79.2	169.7	-0.98	0.49	-0.35	-0.04
2. **generous**	77.6	77.7	177.4	-1.00	0.43	-0.41	-0.02
3. **freedom**	77.5	81.1	162.8	-0.96	0.55	-0.29	-0.03
4. **trustworthy**	76.9	78.3	169.2	-0.98	0.38	-0.45	-0.10
5. **very positive**	75.8	78.2	166.0	-0.97	0.51	-0.31	0.04
6. **wisdom**	75.7	78.5	164.5	-0.96	0.39	-0.43	-0.16
7. **greatly positive**	75.6	78.5	164.4	-0.96	0.52	-0.30	-0.02
8. **honorable**	74.7	75.1	173.7	-0.99	0.43	-0.38	-0.05
9. **positivity**	74.1	77.2	163.5	-0.96	0.52	-0.28	-0.01
10. **positive**	73.9	75.8	167.2	-0.98	0.49	-0.31	-0.02
11. **guarantee**	73.0	74.0	170.5	-0.99	0.37	-0.42	-0.08
12. **healthy**	72.9	74.5	168.1	-0.98	0.36	-0.43	-0.11
13. **respectful**	72.8	73.3	173.6	-0.99	0.40	-0.39	-0.06
14. **optimistic**	72.6	76.0	162.9	-0.96	0.51	-0.28	0.05
15. **brotherhood**	72.2	72.5	174.3	-1.00	0.42	-0.36	0.04
16. **blessing**	71.8	75.0	163.4	-0.96	0.32	-0.46	-0.13
17. **favorable**	71.4	71.7	174.8	-1.00	0.38	-0.40	-0.05
18. **great trust**	71.2	73.6	165.0	-0.97	0.49	-0.28	-0.02
19. **goodness**	69.9	71.3	168.6	-0.98	0.30	-0.45	-0.02
20. **elegance**	69.5	70.4	171.1	-0.99	0.39	-0.36	-0.08

Figure S32: Words with largest components in bad and good directions, within a cone of half angle $\frac{1}{2}\frac{180}{\pi}\cos^{-1}(2/\sqrt{6}) \simeq 17.6°$.

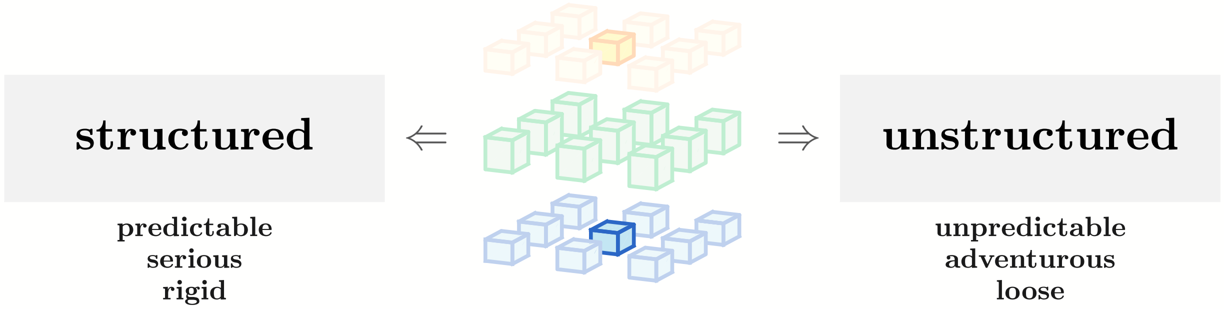

Word	Comp. Size	Size	Angle	Cos.	Pw	Dg	St
1. **century**	37.8	38.6	11.3	0.98	0.05	-0.03	-0.29
2. **archdiocese**	37.3	37.8	8.7	0.99	0.01	-0.04	-0.29
3. **psychologist**	35.7	36.3	11.0	0.98	-0.04	-0.04	-0.27
4. **staid**	34.2	35.8	17.5	0.95	-0.06	-0.06	-0.26
5. **criterion**	34.1	35.7	16.9	0.96	0.08	0.02	-0.26
6. **metropolitan**	33.6	34.8	14.6	0.97	0.06	0.03	-0.26
7. **monastic**	32.9	34.4	16.8	0.96	0.01	-0.08	-0.25
8. **district**	30.6	31.8	16.1	0.96	0.00	-0.07	-0.23
9. **iron**	29.9	31.1	16.0	0.96	0.03	-0.06	-0.23
10. **coding**	29.7	30.8	15.0	0.97	0.02	-0.06	-0.23
11. **clinical**	29.0	30.3	16.8	0.96	-0.02	0.06	-0.22
12. **diction**	28.9	29.7	13.3	0.97	0.04	-0.04	-0.22
13. **feudal**	27.4	28.2	13.4	0.97	-0.05	0.01	-0.21
14. **conservatism**	27.1	27.3	5.8	0.99	-0.01	-0.02	-0.21
15. **utilitarian**	26.8	27.2	9.8	0.99	-0.00	-0.04	-0.21
16. **ground**	26.7	27.8	16.4	0.96	-0.05	-0.03	-0.20
17. **taxonomy**	26.2	26.7	11.3	0.98	-0.01	0.04	-0.20
18. **tense**	24.8	25.6	14.9	0.97	0.03	0.04	-0.19
19. **indenture**	24.7	25.0	8.7	0.99	0.01	-0.03	-0.19
20. **disinfect**	24.5	25.2	12.9	0.97	0.03	-0.03	-0.19

Word	Comp. Size	Size	Angle	Cos.	Pw	Dg	St
1. **plaything**	40.3	42.0	163.7	-0.96	-0.01	-0.09	0.31
2. **joke**	37.1	38.7	163.8	-0.96	0.02	-0.08	0.28
3. **yaaaay**	36.1	36.3	173.4	-0.99	0.02	0.02	0.28
4. **drum**	34.4	34.9	170.9	-0.99	0.03	0.03	0.26
5. **clown**	34.3	35.1	168.2	-0.98	-0.02	-0.05	0.26
6. **bobsled**	34.2	34.9	168.9	-0.98	0.01	-0.05	0.26
7. **potpourri**	33.3	34.5	164.6	-0.96	-0.04	-0.06	0.25
8. **jump rope**	33.0	33.9	166.8	-0.97	0.06	-0.01	0.25
9. **laughable**	33.0	33.4	170.6	-0.99	-0.04	0.01	0.25
10. **ahhhhhhhh**	32.9	34.5	162.5	-0.95	-0.06	0.06	0.25
11. **weeeee**	32.7	34.0	163.7	-0.96	-0.06	0.04	0.25
12. **yaaay**	32.5	32.9	170.8	-0.99	0.00	-0.04	0.25
13. **fanciful**	31.8	33.2	163.2	-0.96	0.02	-0.07	0.24
14. **yayyyy**	31.4	32.8	163.7	-0.96	0.04	-0.06	0.24
15. **serpentine**	30.5	31.0	169.9	-0.98	-0.02	0.04	0.23
16. **yo-yo**	29.9	30.1	173.7	-0.99	0.00	0.03	0.23
17. **jamboree**	28.7	28.7	178.3	-1.00	0.00	0.01	0.22
18. **clowns**	28.4	29.7	162.6	-0.95	0.05	0.04	0.22
19. **yayy**	28.0	28.7	167.4	-0.98	0.01	-0.05	0.21
20. **cantina**	27.9	28.7	166.4	-0.97	0.04	-0.03	0.21

Figure S33: Words with largest components in structured and unstructured directions, within a cone of half angle $\frac{1}{2}\frac{180}{\pi}\cos^{-1}(2/\sqrt{6}) \simeq 17.6^\circ$.

**structured** **safe**	⇐	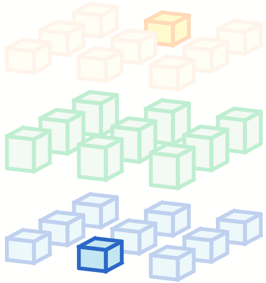	⇒	**unstructured** **dangerous**
(structured-gentle-good) **ordered** **orthodox** **canonical**				(unstructured-aggressive-bad) **jittery** **panicky** **mishcievous**

Word	Comp. Size	Size	Angle	Cos.	Pw	Dg	St
1. **buddhism**	54.4	55.1	9.3	0.99	-0.05	-0.27	-0.32
2. **bishop**	51.5	52.1	8.5	0.99	0.06	-0.28	-0.28
3. **pacific**	51.2	53.6	17.3	0.95	-0.06	-0.35	-0.20
4. **poise**	50.4	52.8	17.4	0.95	0.10	-0.32	-0.22
5. **conservatory**	46.3	48.5	17.4	0.95	0.11	-0.27	-0.23
6. **alphabet**	45.4	45.4	2.5	1.00	0.01	-0.24	-0.25
7. **marble**	45.1	45.5	7.7	0.99	0.03	-0.27	-0.22
8. **statue**	44.7	46.5	16.4	0.96	-0.09	-0.27	-0.22
9. **commonly**	44.4	46.2	16.2	0.96	-0.05	-0.30	-0.18
10. **headline**	43.5	44.9	14.4	0.97	0.07	-0.26	-0.21
11. **full**	42.4	43.7	14.1	0.97	0.08	-0.23	-0.23
12. **document**	41.7	42.2	8.5	0.99	0.04	-0.24	-0.21
13. **sector**	41.6	43.2	16.1	0.96	-0.05	-0.28	-0.17
14. **documentary**	41.2	42.6	14.8	0.97	-0.01	-0.28	-0.16
15. **senor**	40.6	40.8	5.8	0.99	-0.03	-0.21	-0.23
16. **uniform**	39.9	40.0	4.8	1.00	0.03	-0.22	-0.21
17. **therapeutic**	39.2	41.0	17.1	0.96	0.08	-0.24	-0.19
18. **habit**	39.1	40.9	16.8	0.96	-0.01	-0.28	-0.15
19. **storeroom**	38.8	39.5	10.3	0.98	-0.04	-0.23	-0.19
20. **unitary**	38.8	38.9	4.1	1.00	-0.01	-0.20	-0.22

Word	Comp. Size	Size	Angle	Cos.	Pw	Dg	St
1. **nude**	65.4	66.9	167.9	-0.98	-0.02	0.28	0.43
2. **flustered**	63.0	64.4	168.1	-0.98	-0.06	0.40	0.28
3. **edgy**	61.6	64.2	163.5	-0.96	-0.06	0.42	0.24
4. **naked**	60.5	61.9	168.0	-0.98	-0.06	0.38	0.27
5. **jittery**	58.7	60.0	168.1	-0.98	-0.09	0.35	0.29
6. **spook**	58.7	60.1	167.6	-0.98	-0.03	0.38	0.25
7. **madder**	58.1	59.9	166.1	-0.97	-0.09	0.36	0.27
8. **bonkers**	57.2	59.5	164.1	-0.96	-0.09	0.37	0.25
9. **undress**	56.8	57.0	174.4	-1.00	0.04	0.31	0.30
10. **jumpy**	55.6	58.2	162.9	-0.96	-0.13	0.31	0.29
11. **deranged**	53.3	54.1	170.3	-0.99	0.01	0.34	0.24
12. **mischief**	53.1	53.6	172.4	-0.99	-0.05	0.30	0.27
13. **intrigue**	51.1	51.9	169.7	-0.98	-0.04	0.32	0.23
14. **naughty**	49.5	50.0	171.9	-0.99	0.05	0.28	0.26
15. **evacuate**	47.7	49.1	166.3	-0.97	-0.07	0.30	0.22
16. **anal**	47.7	49.6	163.8	-0.96	-0.06	0.32	0.20
17. **gambling**	47.1	48.0	168.4	-0.98	-0.03	0.30	0.21
18. **nudity**	46.7	48.1	166.1	-0.97	0.08	0.28	0.22
19. **eager**	46.7	48.1	166.1	-0.97	0.02	0.19	0.31
20. **sarcastic**	46.4	47.2	169.7	-0.98	-0.06	0.26	0.24

Figure S34: Words with largest components in structured-safe and unstructured-dangerous directions, within a cone of half angle $\frac{1}{2}\frac{180}{\pi}\cos^{-1}(2/\sqrt{6}) \simeq 17.6°$.

structured gentle

(structured-safe-weak)
routine
regulated
ascetic

⇐ 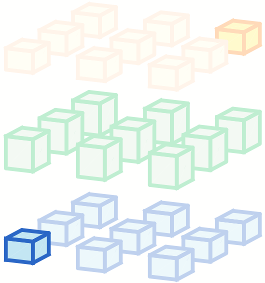 ⇒

unstructured aggressive

(unstructured-dangerous-powerful)
carnal
frenzied
explosive

Word	Comp. Size	Size	Angle	Cos.	Pw	Dg	St
1. **desk**	47.4	48.8	13.7	0.97	-0.23	-0.26	-0.14
2. **elder**	46.0	46.1	3.6	1.00	-0.22	-0.20	-0.19
3. **nun**	45.7	47.3	15.0	0.97	-0.24	-0.25	-0.13
4. **notebook**	44.3	44.6	7.2	0.99	-0.23	-0.19	-0.17
5. **dune**	43.5	44.5	12.1	0.98	-0.24	-0.20	-0.14
6. **blueprint**	42.8	43.8	12.3	0.98	-0.22	-0.22	-0.13
7. **lineal**	42.3	43.3	12.2	0.98	-0.17	-0.24	-0.15
8. **baseboard**	41.5	43.5	17.5	0.95	-0.26	-0.14	-0.15
9. **regular**	41.2	42.3	13.3	0.97	-0.14	-0.17	-0.24
10. **sample**	40.3	41.4	13.6	0.97	-0.17	-0.23	-0.13
11. **point**	40.2	41.1	12.0	0.98	-0.20	-0.21	-0.12
12. **wrench**	40.2	41.6	15.2	0.97	-0.16	-0.13	-0.24
13. **standstill**	39.5	39.5	3.8	1.00	-0.17	-0.16	-0.19
14. **shingle**	39.1	40.9	17.4	0.95	-0.20	-0.22	-0.10
15. **neutrality**	39.1	39.7	10.3	0.98	-0.20	-0.18	-0.13
16. **monk**	38.6	39.1	9.6	0.99	-0.20	-0.18	-0.13
17. **ancient**	38.4	39.2	11.7	0.98	-0.22	-0.14	-0.15
18. **monogram**	38.2	39.2	12.7	0.98	-0.20	-0.19	-0.12
19. **march**	38.2	39.4	14.3	0.97	-0.14	-0.23	-0.14
20. **zen**	38.0	38.9	12.6	0.98	-0.19	-0.20	-0.11

Word	Comp. Size	Size	Angle	Cos.	Pw	Dg	St
1. **sexual**	61.7	63.6	165.8	-0.97	0.36	0.26	0.19
2. **masturbation**	60.4	60.5	176.5	-1.00	0.25	0.29	0.27
3. **lust**	56.8	58.2	167.6	-0.98	0.21	0.33	0.21
4. **striptease**	56.6	58.1	167.1	-0.97	0.33	0.22	0.20
5. **masturbate**	56.0	56.1	177.0	-1.00	0.23	0.26	0.26
6. **playboy**	54.5	55.7	168.4	-0.98	0.28	0.17	0.27
7. **ejaculate**	53.7	55.2	166.8	-0.97	0.32	0.20	0.19
8. **ejaculation**	53.2	53.8	171.2	-0.99	0.28	0.19	0.24
9. **horny**	52.8	53.9	168.7	-0.98	0.23	0.29	0.18
10. **seduction**	51.4	53.4	164.3	-0.96	0.28	0.27	0.14
11. **entice**	50.5	50.7	174.7	-1.00	0.21	0.25	0.21
12. **jump**	50.1	52.4	163.0	-0.96	0.32	0.17	0.18
13. **vagina**	48.7	48.8	177.3	-1.00	0.20	0.22	0.23
14. **agitated**	48.2	50.4	162.8	-0.96	0.15	0.30	0.19
15. **stripper**	47.0	48.7	165.0	-0.97	0.19	0.15	0.28
16. **spirits**	46.6	47.0	173.2	-0.99	0.24	0.18	0.20
17. **clitoris**	46.6	48.0	165.8	-0.97	0.20	0.15	0.27
18. **bustling**	45.2	45.9	169.8	-0.98	0.18	0.25	0.17
19. **impulse**	45.1	46.9	164.2	-0.96	0.26	0.22	0.12
20. **karate**	44.7	46.0	166.3	-0.97	0.26	0.19	0.14

Figure S35: Words with largest components in structured-gentle and unstructured-aggressive directions, within a cone of half angle $\frac{1}{2}\frac{180}{\pi}\cos^{-1}(2/\sqrt{6}) \simeq 17.6°$.

structured weak ⇐

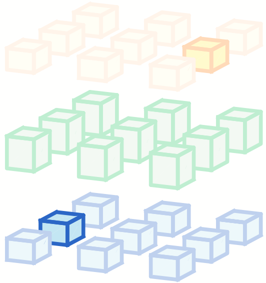

⇒ **unstructured powerful**

(structured-gentle-bad)
inert
detached
closed

(unstructured-aggressive-good)
exciting
celebratory
frolicsome

Word	Comp. Size	Size	Angle	Cos.	Pw	Dg	St
1. **no surprise**	56.0	57.3	12.0	0.98	-0.36	0.04	-0.25
2. **lone**	55.6	57.9	16.2	0.96	-0.39	0.01	-0.21
3. **stone**	55.3	56.1	9.7	0.99	-0.25	-0.01	-0.35
4. **trunk**	52.4	54.3	15.0	0.97	-0.34	-0.07	-0.22
5. **closure**	51.2	52.2	11.3	0.98	-0.33	-0.00	-0.22
6. **lethargic**	50.3	52.6	17.3	0.95	-0.35	0.03	-0.19
7. **finalized**	50.0	52.4	17.2	0.96	-0.19	-0.01	-0.35
8. **standby**	48.1	50.0	16.1	0.96	-0.33	-0.02	-0.19
9. **inactivate**	47.8	48.8	11.8	0.98	-0.31	0.00	-0.20
10. **olden**	46.8	47.9	12.3	0.98	-0.30	-0.04	-0.21
11. **unoccupied**	46.2	47.3	12.7	0.98	-0.31	-0.00	-0.19
12. **baton**	45.5	45.7	5.4	1.00	-0.23	-0.02	-0.27
13. **fragment**	43.7	45.7	17.2	0.96	-0.28	0.08	-0.19
14. **abstention**	42.9	43.0	3.0	1.00	-0.22	0.00	-0.24
15. **distance**	41.6	43.6	17.4	0.95	-0.28	0.06	-0.17
16. **anaesthesia**	40.3	42.0	16.1	0.96	-0.23	-0.09	-0.21
17. **celibacy**	39.0	39.1	3.8	1.00	-0.20	-0.01	-0.22
18. **grate**	38.9	40.7	16.8	0.96	-0.26	-0.05	-0.16
19. **delete**	38.1	39.7	16.2	0.96	-0.27	0.01	-0.15
20. **omit**	37.7	38.4	11.3	0.98	-0.23	-0.04	-0.18

Word	Comp. Size	Size	Angle	Cos.	Pw	Dg	St
1. **great surprise**	81.8	84.9	164.6	-0.96	0.57	-0.01	0.32
2. **carnival**	75.3	75.7	174.3	-1.00	0.44	-0.04	0.38
3. **birthday**	75.0	76.6	168.3	-0.98	0.45	-0.10	0.36
4. **applause**	74.9	77.0	166.8	-0.97	0.48	-0.08	0.33
5. **amusing**	73.0	75.8	164.2	-0.96	0.48	-0.10	0.31
6. **amused**	72.9	74.4	168.4	-0.98	0.46	-0.08	0.33
7. **party**	72.6	73.7	170.3	-0.99	0.42	-0.09	0.37
8. **happynewyear**	72.2	74.2	166.8	-0.97	0.43	-0.12	0.36
9. **excite**	72.2	75.6	162.7	-0.95	0.50	0.08	0.28
10. **kiss**	72.1	75.4	163.0	-0.96	0.48	-0.12	0.30
11. **extreme surprise**	71.7	75.1	162.5	-0.95	0.50	0.07	0.28
12. **fun**	70.6	73.9	162.8	-0.96	0.49	-0.06	0.27
13. **surprise**	69.4	69.7	174.6	-1.00	0.41	-0.01	0.34
14. **dancing**	69.3	69.9	172.1	-0.99	0.33	-0.03	0.42
15. **excitable**	69.2	71.7	164.9	-0.97	0.45	0.09	0.30
16. **humorist**	69.0	72.2	163.0	-0.96	0.44	-0.13	0.30
17. **tasty**	68.6	71.0	165.0	-0.97	0.40	-0.13	0.34
18. **dance**	67.8	68.3	173.1	-0.99	0.40	-0.05	0.34
19. **ecstasy**	67.0	70.3	162.5	-0.95	0.46	0.08	0.26
20. **rollercoaster**	66.4	69.4	163.3	-0.96	0.46	0.07	0.26

Figure S36: Words with largest components in structured-weak and unstructured-powerful directions, within a cone of half angle $\frac{1}{2}\frac{180}{\pi}\cos^{-1}(2/\sqrt{6}) \simeq 17.6°$.

structured
bad

(structured-dangerous-weak)
funereal
contaminated
irreperable

⇐ 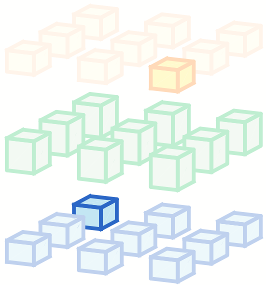 ⇒

unstructured
good

(unstructured-safe-powerful)
playful
joyful
laughing

Word	Comp. Size	Size	Angle	Cos.	Pw	Dg	St
1. **misrepresented**	58.2	60.0	14.2	0.97	-0.24	0.34	-0.19
2. **funeral**	57.7	59.6	14.6	0.97	-0.35	0.22	-0.20
3. **deletion**	56.0	57.4	13.0	0.97	-0.24	0.32	-0.18
4. **irreparable**	55.5	58.2	17.6	0.95	-0.26	0.34	-0.15
5. **improbable**	53.8	54.5	8.8	0.99	-0.29	0.23	-0.20
6. **opposed**	53.4	55.3	15.2	0.96	-0.15	0.27	-0.29
7. **coldness**	52.9	54.1	12.5	0.98	-0.27	0.27	-0.16
8. **bruising**	52.8	55.3	17.6	0.95	-0.25	0.31	-0.13
9. **vile**	52.5	55.1	17.6	0.95	-0.15	0.33	-0.22
10. **extinct**	49.9	52.0	16.3	0.96	-0.26	0.27	-0.13
11. **ineligible**	48.8	49.6	10.4	0.98	-0.27	0.17	-0.21
12. **skeptic**	48.3	48.3	1.8	1.00	-0.21	0.22	-0.21
13. **darkened**	48.3	48.7	7.9	0.99	-0.25	0.22	-0.17
14. **pantheon**	48.0	50.1	16.7	0.96	-0.14	0.20	-0.29
15. **annulment**	47.6	48.7	12.1	0.98	-0.27	0.16	-0.20
16. **farewell**	47.5	49.8	17.4	0.95	-0.12	0.27	-0.24
17. **comatose**	47.3	48.1	10.8	0.98	-0.26	0.21	-0.16
18. **deflect**	47.2	48.0	10.2	0.98	-0.20	0.26	-0.17
19. **stereotype**	46.8	48.9	17.2	0.96	-0.13	0.20	-0.29
20. **halt**	46.2	48.0	16.0	0.96	-0.28	0.14	-0.19

Word	Comp. Size	Size	Angle	Cos.	Pw	Dg	St
1. **smiling**	66.1	69.0	163.3	-0.96	0.42	-0.22	0.24
2. **romance**	66.0	68.0	166.2	-0.97	0.39	-0.22	0.27
3. **honeymoon**	65.8	66.8	170.2	-0.99	0.34	-0.22	0.31
4. **laugh**	65.0	66.2	168.7	-0.98	0.37	-0.25	0.25
5. **sweetie**	64.0	64.8	171.0	-0.99	0.31	-0.32	0.22
6. **laughter**	62.6	64.3	167.1	-0.97	0.37	-0.22	0.24
7. **scrumptious**	62.6	63.6	169.4	-0.98	0.35	-0.23	0.26
8. **fiancee**	62.0	62.6	171.9	-0.99	0.33	-0.24	0.25
9. **liking**	61.9	63.0	169.0	-0.98	0.34	-0.26	0.22
10. **cheery**	61.5	62.1	171.7	-0.99	0.29	-0.22	0.31
11. **goodies**	60.8	61.7	170.1	-0.99	0.23	-0.24	0.33
12. **adorable**	60.6	61.6	169.9	-0.98	0.21	-0.33	0.26
13. **loving**	60.1	62.9	163.1	-0.96	0.37	-0.24	0.18
14. **travel**	60.0	61.6	166.7	-0.97	0.33	-0.18	0.29
15. **lovee**	59.8	61.6	166.1	-0.97	0.35	-0.26	0.19
16. **hearts**	59.6	62.2	163.4	-0.96	0.36	-0.25	0.17
17. **lovelovelove**	59.4	59.9	173.0	-0.99	0.30	-0.27	0.22
18. **happyheart**	59.4	61.0	166.7	-0.97	0.34	-0.27	0.18
19. **memories**	59.0	60.5	167.2	-0.98	0.23	-0.34	0.21
20. **delicious**	58.4	60.2	166.0	-0.97	0.35	-0.20	0.22

Figure S37: Words with largest components in structured-bad and unstructured-good directions, within a cone of half angle $\frac{1}{2}\frac{180}{\pi}\cos^{-1}(2/\sqrt{6}) \simeq 17.6°$**.**

unstructured safe ⇐ 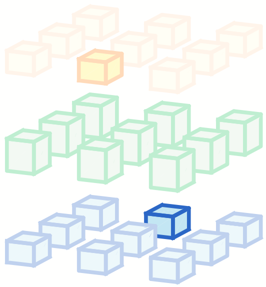 ⇒ **structured dangerous**

(unstructured-gentle-good)
nurturing
wholesome
sweet

(structured-aggressive-bad)
authoritarian
controlling
adversarial

Word	Comp. Size	Size	Angle	Cos.	Pw	Dg	St
1. **popcorn**	60.6	60.8	4.5	1.00	-0.04	-0.32	0.33
2. **sweetest**	59.7	62.3	16.5	0.96	0.13	-0.34	0.31
3. **fruits**	58.7	60.1	12.4	0.98	-0.06	-0.37	0.26
4. **sweets**	58.6	59.3	8.7	0.99	0.04	-0.36	0.28
5. **butterfly**	58.4	61.0	16.9	0.96	-0.02	-0.41	0.22
6. **toy**	58.2	58.4	4.5	1.00	-0.01	-0.34	0.29
7. **puppy**	57.7	58.3	8.0	0.99	0.06	-0.33	0.30
8. **pet**	57.1	59.6	16.8	0.96	-0.04	-0.40	0.22
9. **candy**	56.6	59.0	16.5	0.96	0.09	-0.37	0.24
10. **childhood**	54.7	55.1	6.9	0.99	0.00	-0.33	0.26
11. **comic**	54.5	56.7	16.1	0.96	0.11	-0.33	0.25
12. **colors**	54.4	56.8	16.5	0.96	-0.00	-0.38	0.21
13. **reminisce**	54.1	55.6	13.3	0.97	0.10	-0.30	0.28
14. **decorative**	53.9	55.8	15.0	0.97	0.10	-0.33	0.26
15. **pizza**	53.8	54.6	9.4	0.99	-0.03	-0.34	0.25
16. **kitten**	53.4	55.7	16.7	0.96	-0.09	-0.35	0.23
17. **nursery**	52.5	54.9	16.9	0.96	0.07	-0.35	0.21
18. **pie**	52.4	54.1	14.3	0.97	0.05	-0.35	0.22
19. **risotto**	52.1	52.9	10.0	0.98	-0.06	-0.26	0.30
20. **fruitcake**	51.0	51.8	10.2	0.98	0.05	-0.31	0.24

Word	Comp. Size	Size	Angle	Cos.	Pw	Dg	St
1. **demolish**	56.9	58.1	168.1	-0.98	0.02	0.37	-0.24
2. **protocol**	55.0	56.2	168.2	-0.98	0.05	0.25	-0.35
3. **rigidity**	49.2	49.4	174.4	-1.00	0.03	0.25	-0.28
4. **irreversible**	48.4	48.6	175.5	-1.00	-0.02	0.27	-0.25
5. **obligation**	46.8	47.8	168.5	-0.98	0.07	0.28	-0.23
6. **narcissist**	46.5	48.3	164.5	-0.96	-0.02	0.32	-0.18
7. **litigant**	45.4	45.9	172.3	-0.99	0.04	0.26	-0.23
8. **jailer**	44.6	46.7	162.7	-0.95	0.01	0.32	-0.17
9. **antagonist**	43.9	45.4	165.2	-0.97	0.07	0.28	-0.20
10. **judging**	43.5	45.3	163.9	-0.96	0.07	0.28	-0.19
11. **carnivorous**	42.3	43.7	165.8	-0.97	0.03	0.28	-0.17
12. **forensics**	42.1	42.2	176.6	-1.00	0.02	0.23	-0.23
13. **curfew**	41.1	42.9	163.1	-0.96	0.01	0.29	-0.15
14. **induce**	40.8	42.1	165.5	-0.97	0.06	0.26	-0.19
15. **python**	40.7	42.7	162.6	-0.95	0.00	0.29	-0.15
16. **penal**	40.7	41.6	167.9	-0.98	-0.03	0.26	-0.18
17. **cardiology**	40.6	41.6	168.0	-0.98	-0.05	0.25	-0.19
18. **critic**	40.1	41.5	165.1	-0.97	0.07	0.25	-0.18
19. **smug**	40.1	41.6	164.4	-0.96	-0.06	0.26	-0.18
20. **revocation**	39.8	41.2	164.8	-0.97	0.06	0.26	-0.17

Figure S38: Words with largest components in unstructured-safe and structured-dangerous directions, within a cone of half angle $\frac{1}{2}\frac{180}{\pi}\cos^{-1}(2/\sqrt{6}) \simeq 17.6^{\circ}$.

unstructured gentle ⇐ 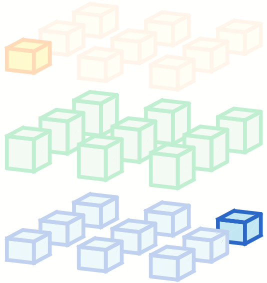 ⇒ **structured aggressive**

(unstructured-safe-weak)
whimsical
childlike
yielding

(structured-dangerous-powerful)
dictatorial
militaristic
imperious

Word	Comp. Size	Size	Angle	Cos.	Pw	Dg	St
1. **feather**	62.2	64.4	15.3	0.96	-0.31	-0.34	0.17
2. **pudding**	57.5	60.0	16.9	0.96	-0.21	-0.36	0.19
3. **lotion**	57.3	59.1	14.0	0.97	-0.23	-0.34	0.19
4. **wig**	57.3	58.5	11.7	0.98	-0.23	-0.33	0.21
5. **plum**	57.2	57.9	9.1	0.99	-0.22	-0.31	0.23
6. **burrito**	56.3	56.8	7.9	0.99	-0.25	-0.29	0.21
7. **foam**	55.7	58.4	17.5	0.95	-0.24	-0.34	0.15
8. **puree**	54.6	56.7	15.8	0.96	-0.22	-0.33	0.17
9. **bunny**	54.5	55.7	11.8	0.98	-0.19	-0.31	0.23
10. **foaming**	52.9	55.3	16.8	0.96	-0.27	-0.30	0.13
11. **platter**	52.7	55.2	17.4	0.95	-0.23	-0.32	0.14
12. **weave**	52.5	54.4	15.1	0.97	-0.30	-0.25	0.15
13. **shoes**	52.2	53.5	12.3	0.98	-0.18	-0.30	0.21
14. **cream**	52.0	54.1	16.1	0.96	-0.22	-0.31	0.15
15. **crayon**	51.4	53.5	16.2	0.96	-0.29	-0.25	0.14
16. **butter**	50.4	51.4	10.9	0.98	-0.24	-0.26	0.16
17. **sleeveless**	50.1	51.1	11.4	0.98	-0.25	-0.26	0.16
18. **littlethings**	49.9	52.2	17.1	0.96	-0.16	-0.31	0.19
19. **froth**	49.5	50.9	13.1	0.97	-0.27	-0.24	0.15
20. **lego**	49.1	50.5	13.3	0.97	-0.27	-0.23	0.15

Word	Comp. Size	Size	Angle	Cos.	Pw	Dg	St
1. **boss**	65.8	66.4	172.8	-0.99	0.29	0.24	-0.33
2. **dictatorial**	65.7	68.6	163.1	-0.96	0.23	0.41	-0.23
3. **exterminator**	65.0	67.4	164.6	-0.96	0.35	0.34	-0.18
4. **relentless**	63.2	63.5	174.2	-0.99	0.31	0.28	-0.24
5. **imperialist**	61.4	63.9	164.0	-0.96	0.27	0.37	-0.18
6. **army**	60.6	63.4	163.1	-0.96	0.37	0.26	-0.18
7. **political**	60.0	60.8	170.8	-0.99	0.31	0.29	-0.21
8. **cop**	58.7	60.6	165.4	-0.97	0.29	0.33	-0.17
9. **policy**	56.9	58.0	168.9	-0.98	0.27	0.30	-0.19
10. **courtroom**	56.5	57.2	171.2	-0.99	0.30	0.22	-0.23
11. **armada**	56.5	58.6	164.6	-0.96	0.34	0.25	-0.17
12. **military**	56.5	57.7	168.1	-0.98	0.32	0.24	-0.19
13. **commando**	54.0	54.8	170.5	-0.99	0.24	0.29	-0.19
14. **imposing**	53.4	55.1	165.9	-0.97	0.30	0.25	-0.16
15. **demanding**	53.2	55.5	163.4	-0.96	0.30	0.26	-0.14
16. **regimental**	52.2	52.6	172.9	-0.99	0.19	0.26	-0.24
17. **manipulate**	52.2	54.5	163.4	-0.96	0.17	0.33	-0.20
18. **claimant**	51.7	53.3	165.9	-0.97	0.18	0.31	-0.20
19. **politician**	51.6	53.1	166.2	-0.97	0.20	0.18	-0.31
20. **courts**	51.6	52.5	169.2	-0.98	0.26	0.25	-0.17

Figure S39: Words with largest components in unstructured-gentle and structured-aggressive directions, within a cone of half angle $\frac{1}{2}\frac{180}{\pi}\cos^{-1}(2/\sqrt{6}) \simeq 17.6°$.

unstructured weak ⇐

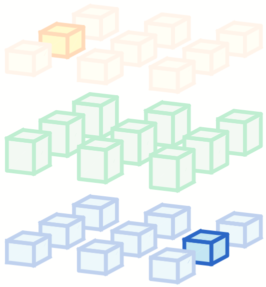

⇒ **structured powerful**

(unstructured-gentle-bad)
inchoate
hazy
drifting

(structured-aggressive-good)
commanding
authorative
hierarchical

Word	Comp. Size	Size	Angle	Cos.	Pw	Dg	St
1. **vagabond**	46.9	49.1	17.3	0.95	-0.30	0.09	0.21
2. **minion**	43.0	44.5	14.6	0.97	-0.28	-0.06	0.19
3. **roll**	42.9	43.1	4.7	1.00	-0.25	0.02	0.22
4. **vanquished**	42.5	42.5	2.4	1.00	-0.22	0.01	0.24
5. **cheesy**	41.8	42.3	9.1	0.99	-0.26	-0.01	0.19
6. **loopy**	40.0	40.8	10.7	0.98	-0.24	0.05	0.19
7. **underestimate**	40.0	42.0	17.6	0.95	-0.28	0.02	0.15
8. **vassal**	40.0	41.3	14.5	0.97	-0.25	0.06	0.18
9. **need**	39.6	40.4	11.4	0.98	-0.18	0.03	0.25
10. **dubious**	39.4	41.1	16.4	0.96	-0.27	0.04	0.16
11. **murmur**	39.4	39.7	7.2	0.99	-0.23	0.03	0.20
12. **juju**	38.4	38.9	8.8	0.99	-0.18	-0.03	0.23
13. **babble**	38.2	40.0	17.5	0.95	-0.22	-0.09	0.20
14. **hide**	37.0	37.8	11.6	0.98	-0.23	0.04	0.17
15. **leftover**	36.1	36.7	11.0	0.98	-0.23	-0.03	0.17
16. **scampi**	35.6	36.9	15.1	0.97	-0.21	-0.07	0.18
17. **hesitate**	35.4	36.0	10.7	0.98	-0.19	0.05	0.19
18. **shin**	35.2	35.6	8.3	0.99	-0.22	0.02	0.17
19. **ska**	33.8	35.2	15.9	0.96	-0.17	0.07	0.20
20. **ahhh**	33.8	34.9	14.3	0.97	-0.15	-0.04	0.22

Word	Comp. Size	Size	Angle	Cos.	Pw	Dg	St
1. **commissioner**	59.0	60.5	167.5	-0.98	0.35	-0.09	-0.29
2. **superintendent**	57.4	59.6	164.1	-0.96	0.40	0.00	-0.22
3. **supervisor**	56.8	58.6	165.9	-0.97	0.36	-0.08	-0.25
4. **business man**	55.9	58.1	164.2	-0.96	0.38	0.06	-0.23
5. **directive**	54.5	56.4	165.2	-0.97	0.37	-0.04	-0.22
6. **producer**	54.1	55.6	166.9	-0.97	0.36	0.02	-0.23
7. **governmental**	52.7	53.3	171.2	-0.99	0.32	0.03	-0.25
8. **administrator**	52.6	52.8	175.4	-1.00	0.30	-0.02	-0.27
9. **magistrate**	52.6	54.2	165.9	-0.97	0.36	-0.02	-0.21
10. **reformer**	52.5	54.2	165.4	-0.97	0.36	0.02	-0.21
11. **patriarch**	52.4	53.8	167.0	-0.97	0.31	-0.09	-0.26
12. **minister**	51.6	52.1	171.7	-0.99	0.28	-0.06	-0.28
13. **controlling**	50.5	50.9	173.6	-0.99	0.25	-0.02	-0.30
14. **warrants**	50.2	51.2	168.8	-0.98	0.27	-0.08	-0.27
15. **colonel**	50.2	52.6	162.7	-0.95	0.31	0.11	-0.24
16. **officer**	49.7	50.4	170.5	-0.99	0.26	-0.06	-0.28
17. **congressman**	49.2	50.0	169.7	-0.98	0.23	0.04	-0.30
18. **juridical**	49.1	49.6	171.9	-0.99	0.25	-0.05	-0.28
19. **patron**	48.7	49.9	167.3	-0.98	0.29	-0.08	-0.24
20. **legislator**	48.6	50.3	165.1	-0.97	0.31	0.08	-0.22

Figure S40: Words with largest components in unstructured-weak and structured-powerful directions, within a cone of half angle $\frac{1}{2}\frac{180}{\pi}\cos^{-1}(2/\sqrt{6}) \simeq 17.6°$.

unstructured
bad

(unstructured-dangerous-weak)
confused
mocked
messy

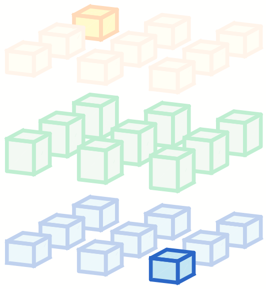

structured
good

(structured-safe-powerful)
wise
respected
stable

Word	Comp. Size	Size	Angle	Cos.	Pw	Dg	St
1. **slave**	71.3	73.6	14.2	0.97	-0.28	0.43	0.24
2. **skittish**	71.1	74.5	17.3	0.95	-0.26	0.45	0.23
3. **vomiting**	70.7	73.2	15.2	0.97	-0.29	0.43	0.22
4. **lost**	62.5	64.1	12.9	0.97	-0.33	0.32	0.19
5. **downfall**	62.0	63.8	13.3	0.97	-0.28	0.35	0.19
6. **stifled**	60.1	61.4	12.0	0.98	-0.25	0.34	0.21
7. **mocked**	60.0	61.8	13.7	0.97	-0.23	0.36	0.21
8. **wobbly**	59.6	62.4	17.3	0.95	-0.22	0.38	0.19
9. **inexperience**	58.9	61.3	15.9	0.96	-0.34	0.27	0.16
10. **unsettled**	58.8	60.7	14.1	0.97	-0.31	0.30	0.17
11. **cockroach**	58.4	60.9	16.5	0.96	-0.30	0.33	0.15
12. **messy**	58.3	59.2	9.8	0.99	-0.32	0.25	0.21
13. **queasiness**	58.2	60.6	16.1	0.96	-0.35	0.24	0.17
14. **confused**	57.4	59.0	13.3	0.97	-0.30	0.29	0.17
15. **twit**	57.1	59.6	16.8	0.96	-0.30	0.31	0.15
16. **flee**	56.2	58.8	17.1	0.96	-0.16	0.34	0.25
17. **impotence**	56.2	58.9	17.3	0.95	-0.26	0.34	0.15
18. **unorganized**	56.2	56.8	8.6	0.99	-0.28	0.27	0.20
19. **shiver**	55.8	58.1	15.9	0.96	-0.18	0.34	0.21
20. **shaky**	55.4	56.8	12.7	0.98	-0.21	0.32	0.20

Word	Comp. Size	Size	Angle	Cos.	Pw	Dg	St
1. **confidence**	84.2	88.3	162.6	-0.95	0.44	-0.47	-0.21
2. **reliable**	76.6	80.2	162.6	-0.95	0.35	-0.46	-0.20
3. **wise**	72.7	75.7	163.9	-0.96	0.33	-0.43	-0.20
4. **secure**	68.9	72.0	163.0	-0.96	0.41	-0.31	-0.19
5. **respectable**	68.3	71.1	164.0	-0.96	0.38	-0.34	-0.18
6. **constitute**	68.0	69.7	167.3	-0.98	0.38	-0.31	-0.21
7. **stability**	67.0	68.4	168.0	-0.98	0.30	-0.37	-0.22
8. **autonomy**	64.7	65.9	169.2	-0.98	0.35	-0.29	-0.22
9. **educator**	64.4	66.5	165.4	-0.97	0.32	-0.35	-0.18
10. **soundness**	63.3	65.0	166.7	-0.97	0.36	-0.27	-0.20
11. **sacred**	63.2	66.3	162.4	-0.95	0.37	-0.31	-0.16
12. **parent**	62.8	64.9	165.2	-0.97	0.35	-0.31	-0.18
13. **maestro**	62.3	64.8	163.9	-0.96	0.34	-0.32	-0.16
14. **civilized**	62.3	64.7	164.3	-0.96	0.23	-0.38	-0.21
15. **propriety**	61.8	64.6	163.1	-0.96	0.27	-0.38	-0.17
16. **securities**	61.5	61.9	173.6	-0.99	0.29	-0.30	-0.23
17. **property**	60.8	62.4	167.1	-0.97	0.21	-0.35	-0.25
18. **father**	60.4	62.6	164.8	-0.96	0.31	-0.33	-0.16
19. **proprietary**	60.3	61.4	168.8	-0.98	0.26	-0.34	-0.21
20. **order**	59.8	61.1	168.1	-0.98	0.27	-0.33	-0.19

Figure S41: Words with largest components in unstructured-bad and structured-good directions, within a cone of half angle $\frac{1}{2}\frac{180}{\pi}\cos^{-1}(2/\sqrt{6}) \simeq 17.6°$.

S5 Ousiogram analysis sequences for real corpora

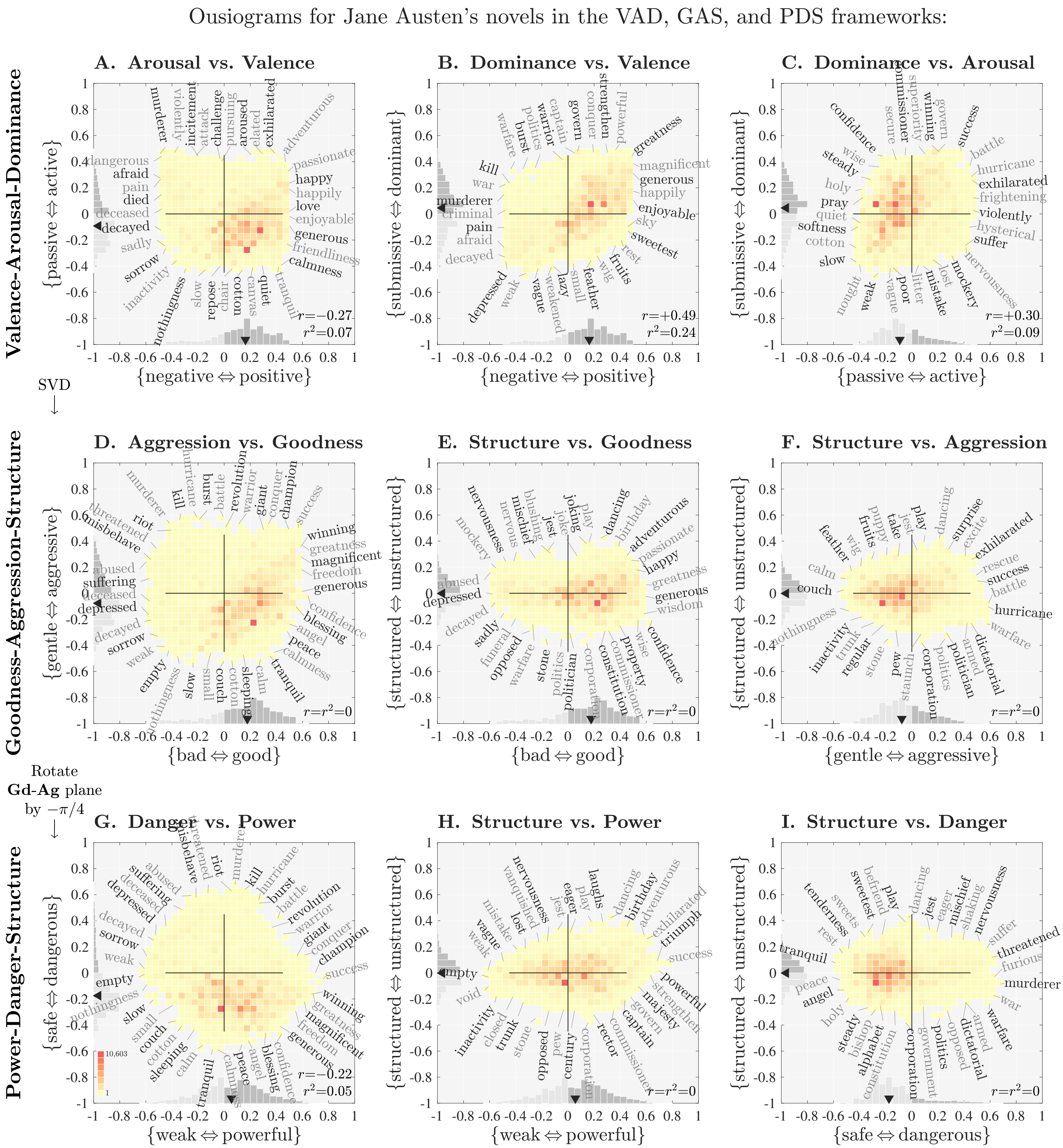

Figure S42: Ousiograms showing the VAD-GAS-PDS analytic sequence for Jane Austen's writings. The novels are "Sense and Sensibility," "Pride and Prejudice," "Mansfield Park," "Emma," "Northanger Abbey," and "Persuasion," published in 1811–1818. We obtained all novels from the Gutenberg Project: http://www.gutenberg.org. The underlying word frequency distribution is built by merging all books and then constructing a word frequency distribution. Panel G corresponds to Fig. 6A.

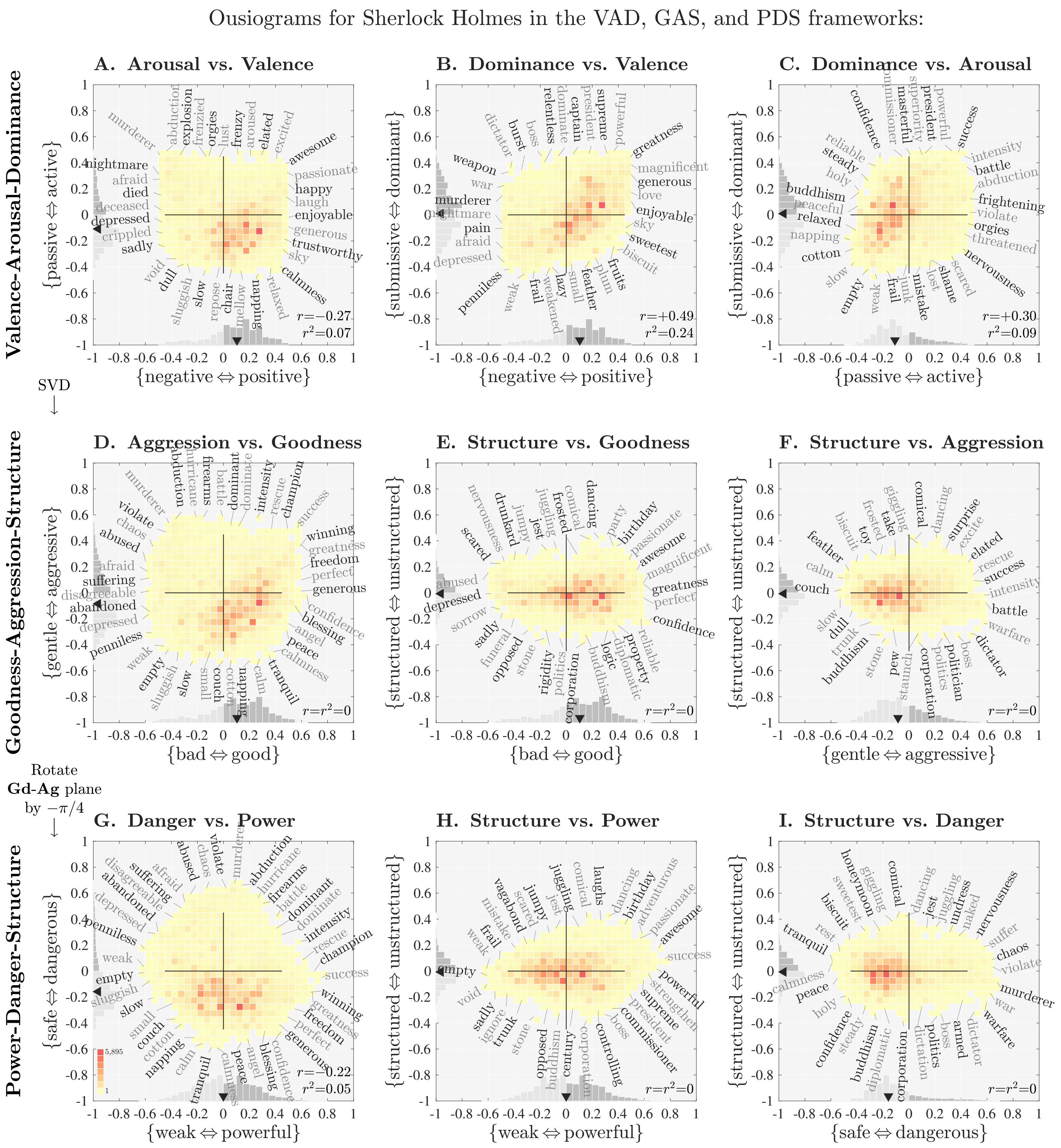

Figure S43: Ousiograms showing the VAD-GAS-PDS analytic sequence for Sir Arthur Conan Doyle's Sherlock Holmes novels and short stories. We obtained four novels and forty-four short stories from the complete Sherlock Holmes Canon https://sherlock-holm.es/ (due to copyright, twelve short stories contained in the "Case-Book of Sherlock Holmes" were not available from this source). The underlying word frequency distribution is built by merging all books and then constructing a word frequency distribution. Panel G corresponds to Fig. 6B.

Ousiograms for the New York Times in the VAD, GAS, and PDS frameworks:

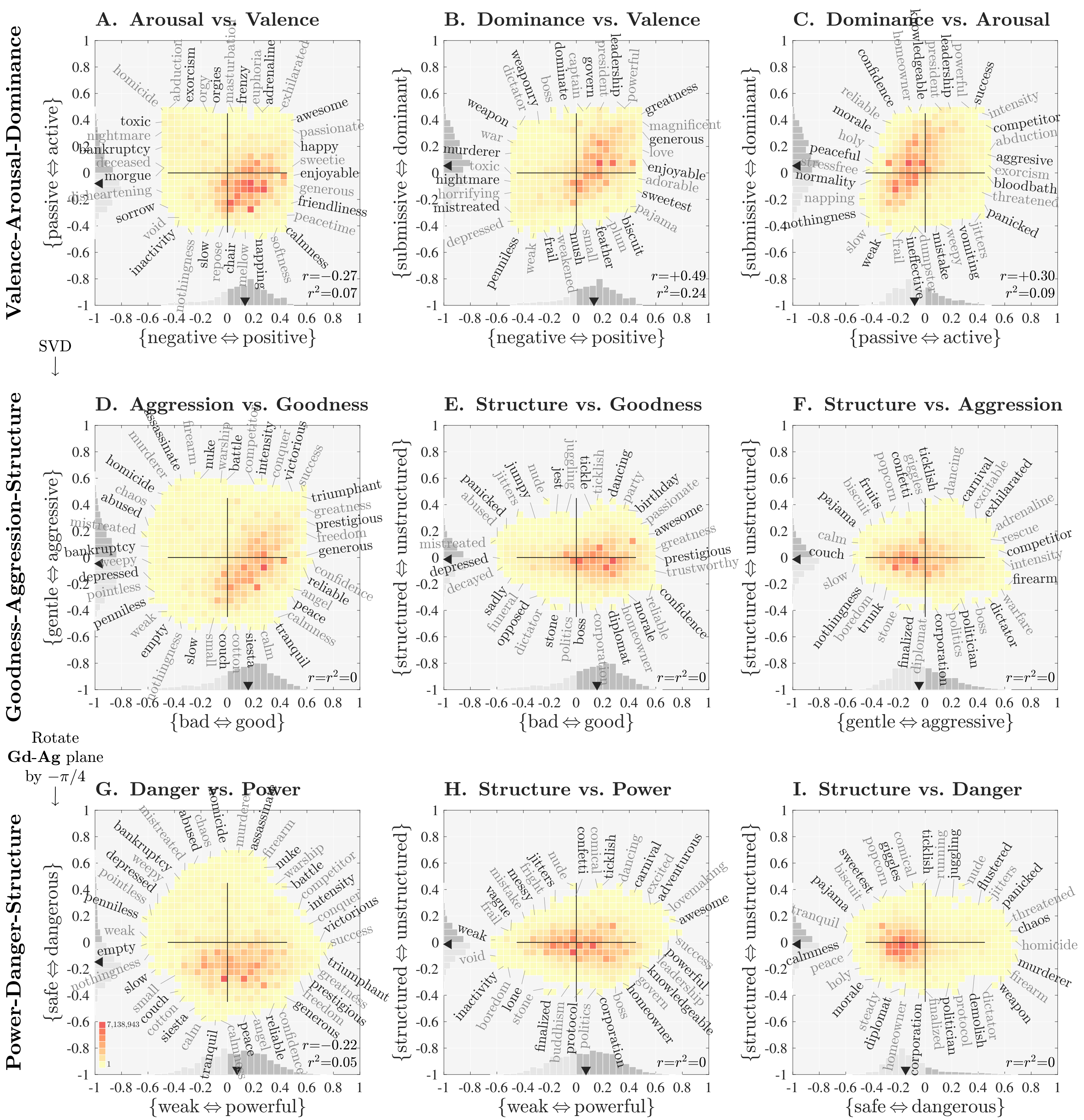

Figure S44: Ousiograms showing the VAD-GAS-PDS analytic sequence for the New York Times. The underlying word frequency distribution is built from a 1987–2007 annotated corpus [54]. Panel G corresponds to Fig. 6C.

Ousiograms for Wikipedia in the VAD, GAS, and PDS frameworks:

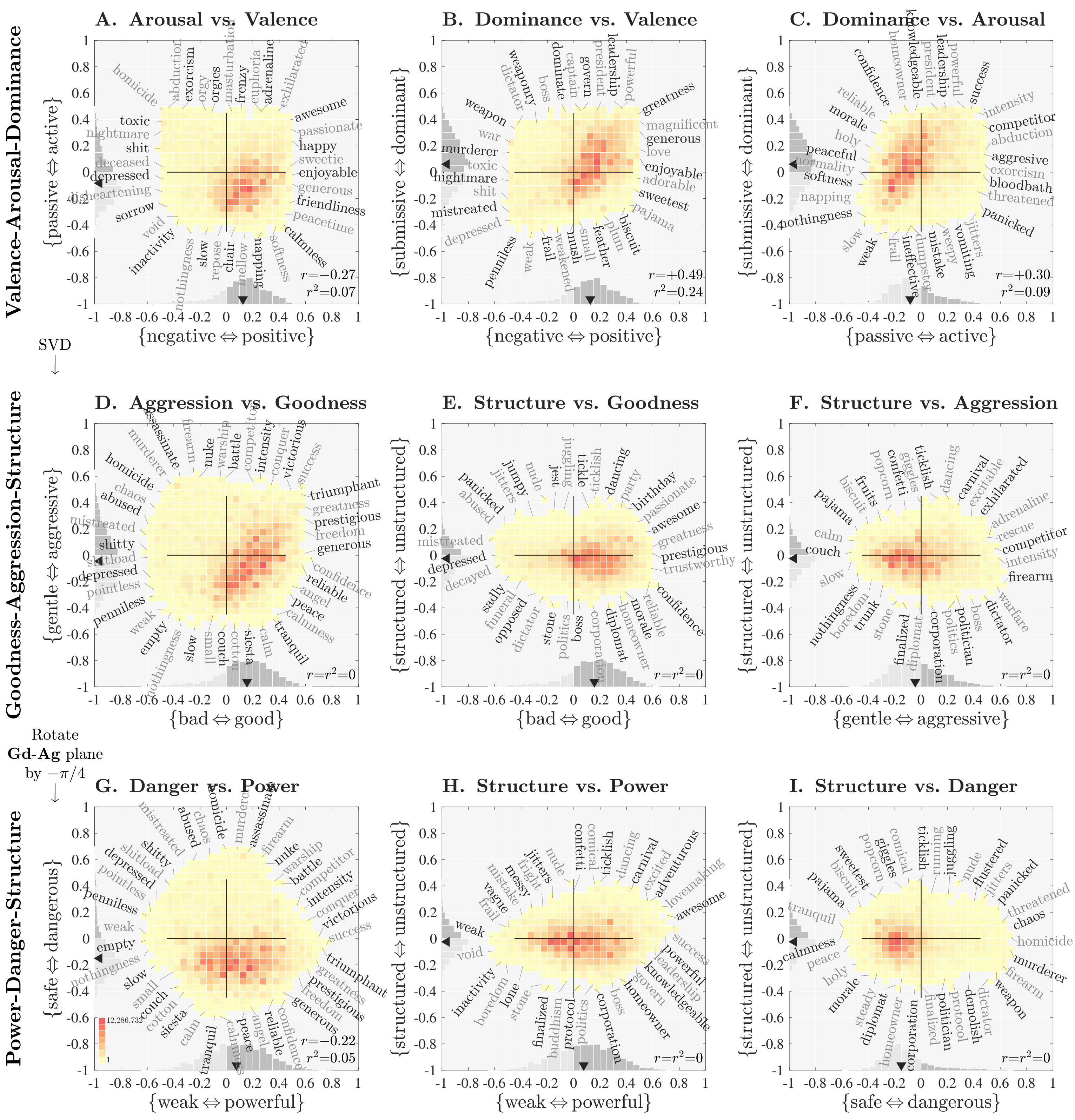

Figure S45: Ousiograms showing the VAD-GAS-PDS analytic sequence for Wikipedia. The underlying word frequency distribution is based on the March 2019 dump of the English Wikipedia [55]. Panel G corresponds to Fig. 6D.

Ousiograms for RadioTalk in the VAD, GAS, and PDS frameworks:

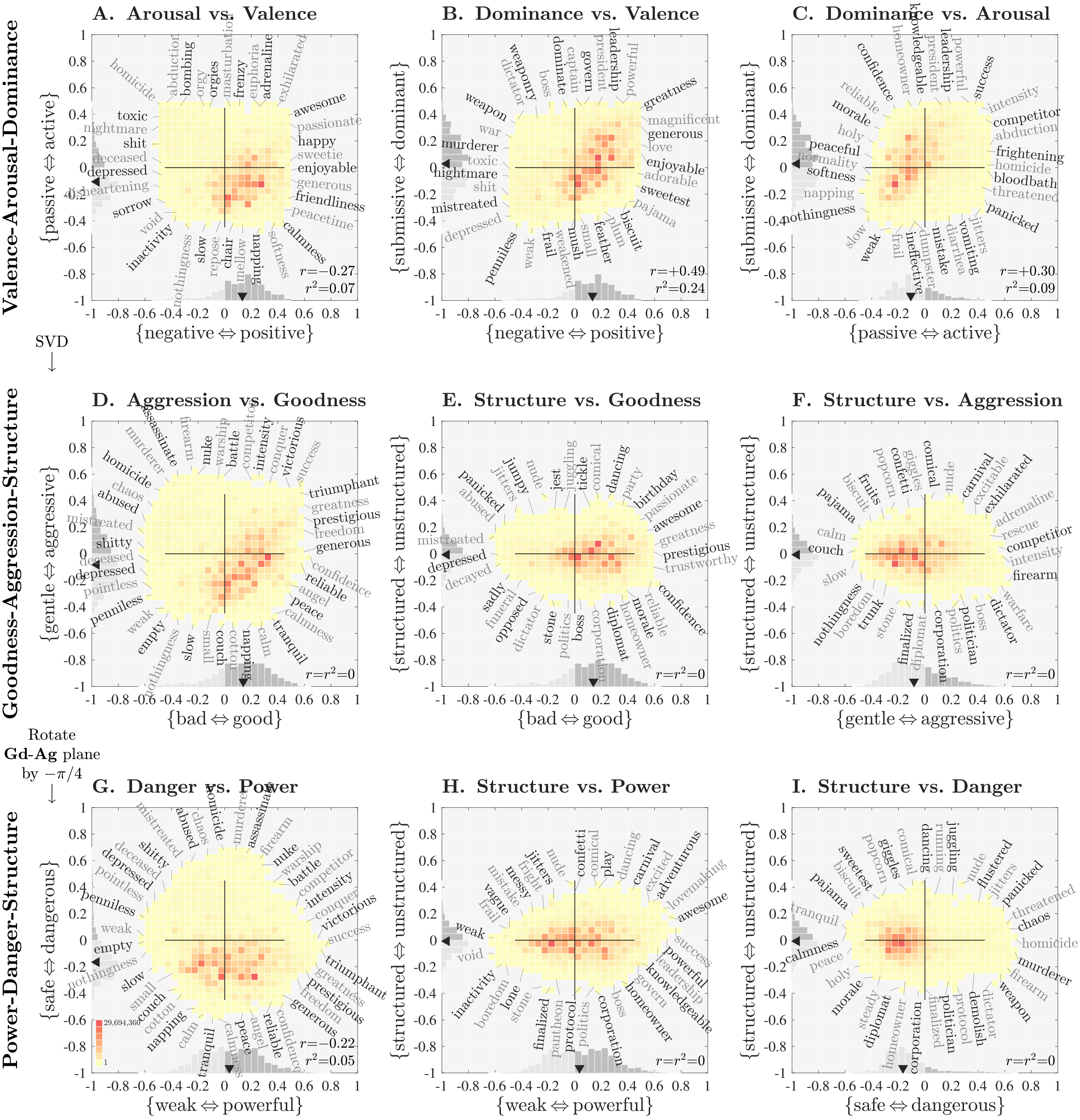

Figure S46: Ousiograms showing the VAD-GAS-PDS analytic sequence for the RadioTalk corpus. The underlying word frequency distribution is derived from a corpus of automated transcriptions of talk radio in the US covering the time period 2018/10–2019/03 [56]. Panel G corresponds to Fig. 6E.

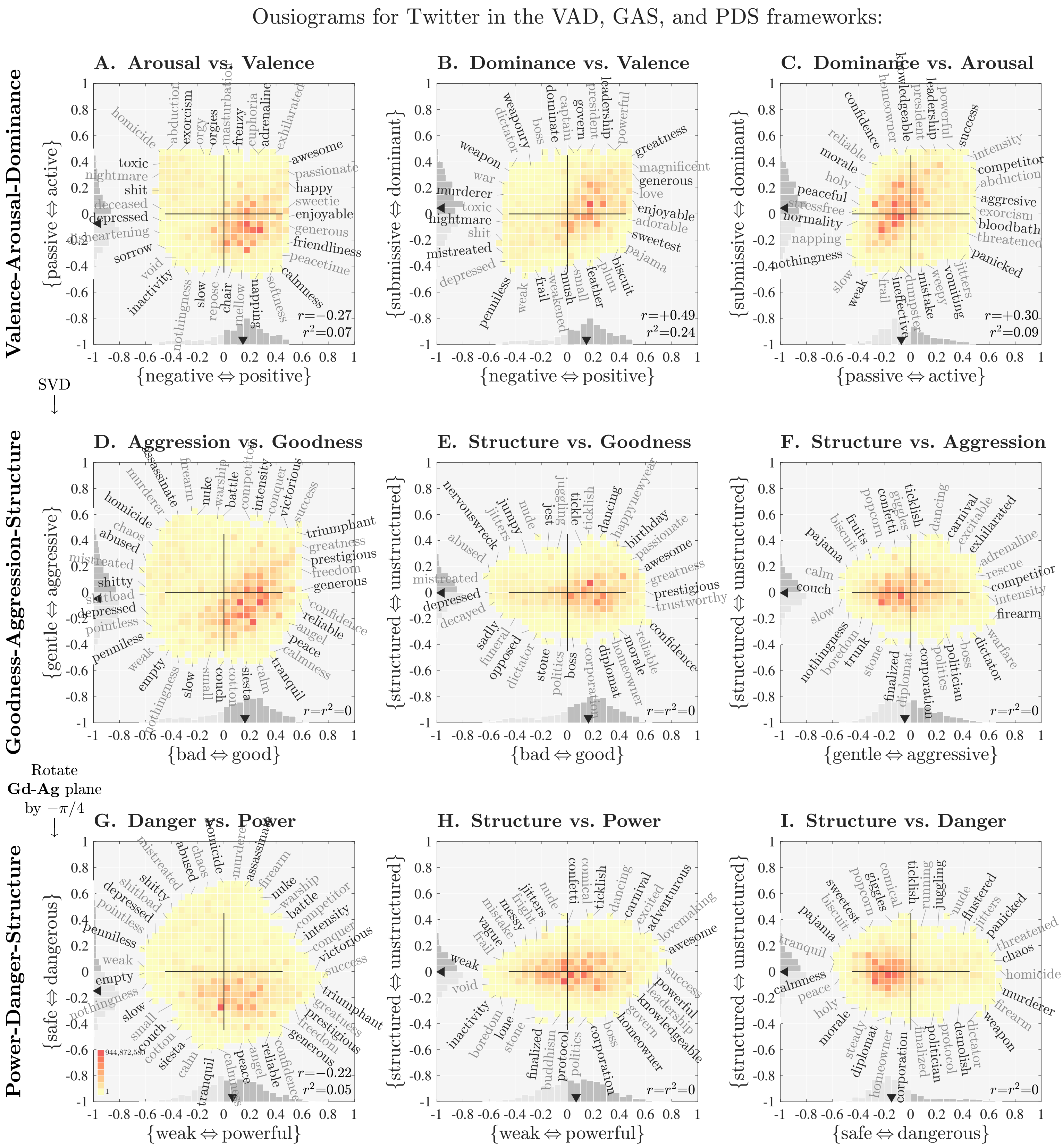

Figure S47: Ousiograms showing the VAD-GAS-PDS analytic sequence for Twitter. The underlying word frequency distribution is an equal weighting of day-scale word frequency distributions derived from approximately 10% of English tweets in 2020 [57]. In contrast to the word frequency distributions obtained from ‘flat’ corpora, the word frequency distribution for Twitter encodes a strong sense of popularity as social amplification is naturally included through retweets. Panel G corresponds to Fig. 6F.

S6 Ousiometric time series and trajectory for Les Misérables: Flipbook

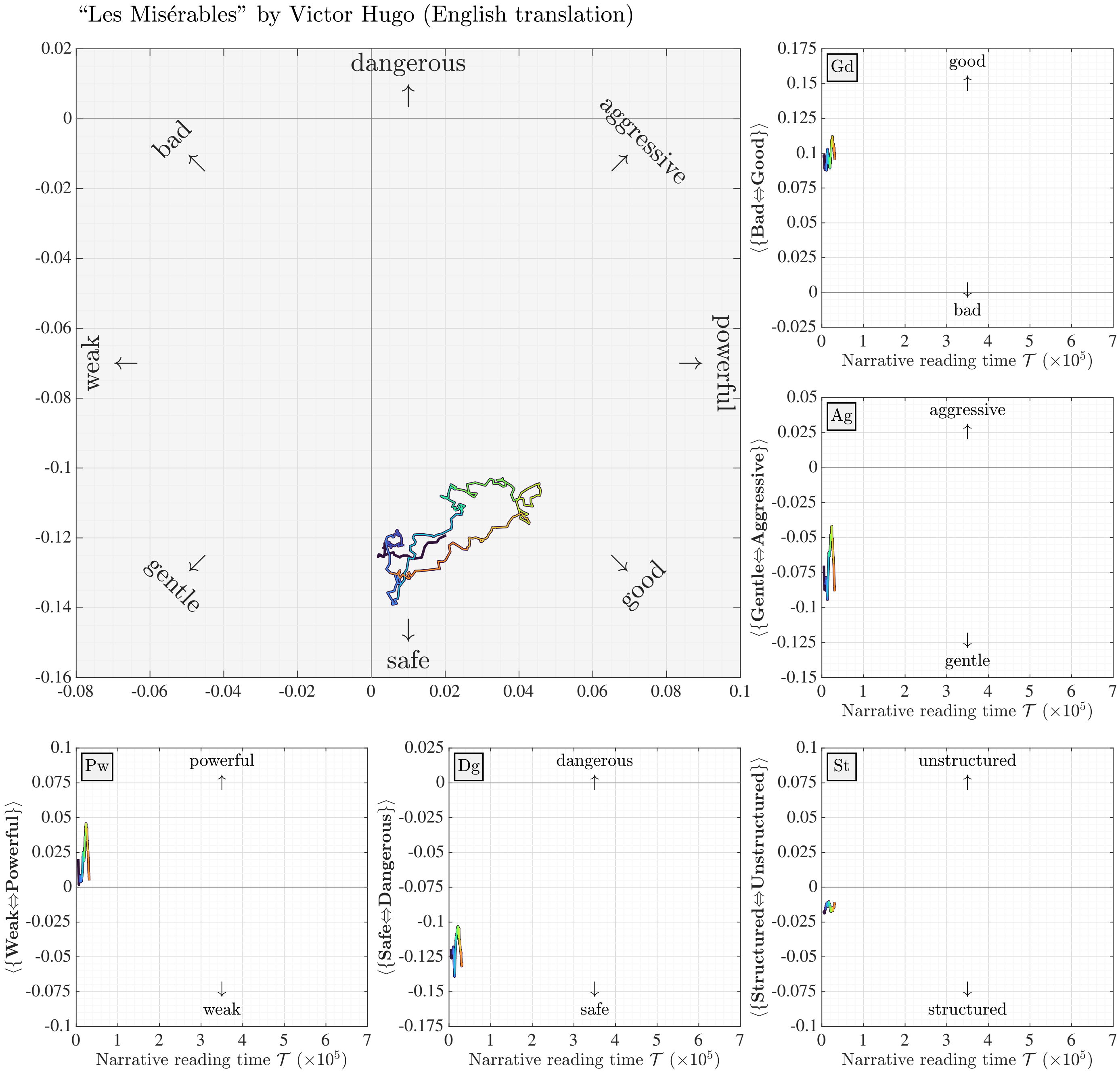

Figure S48: Epoch 1 of 25 in Victor Hugo's "Les Misérables."

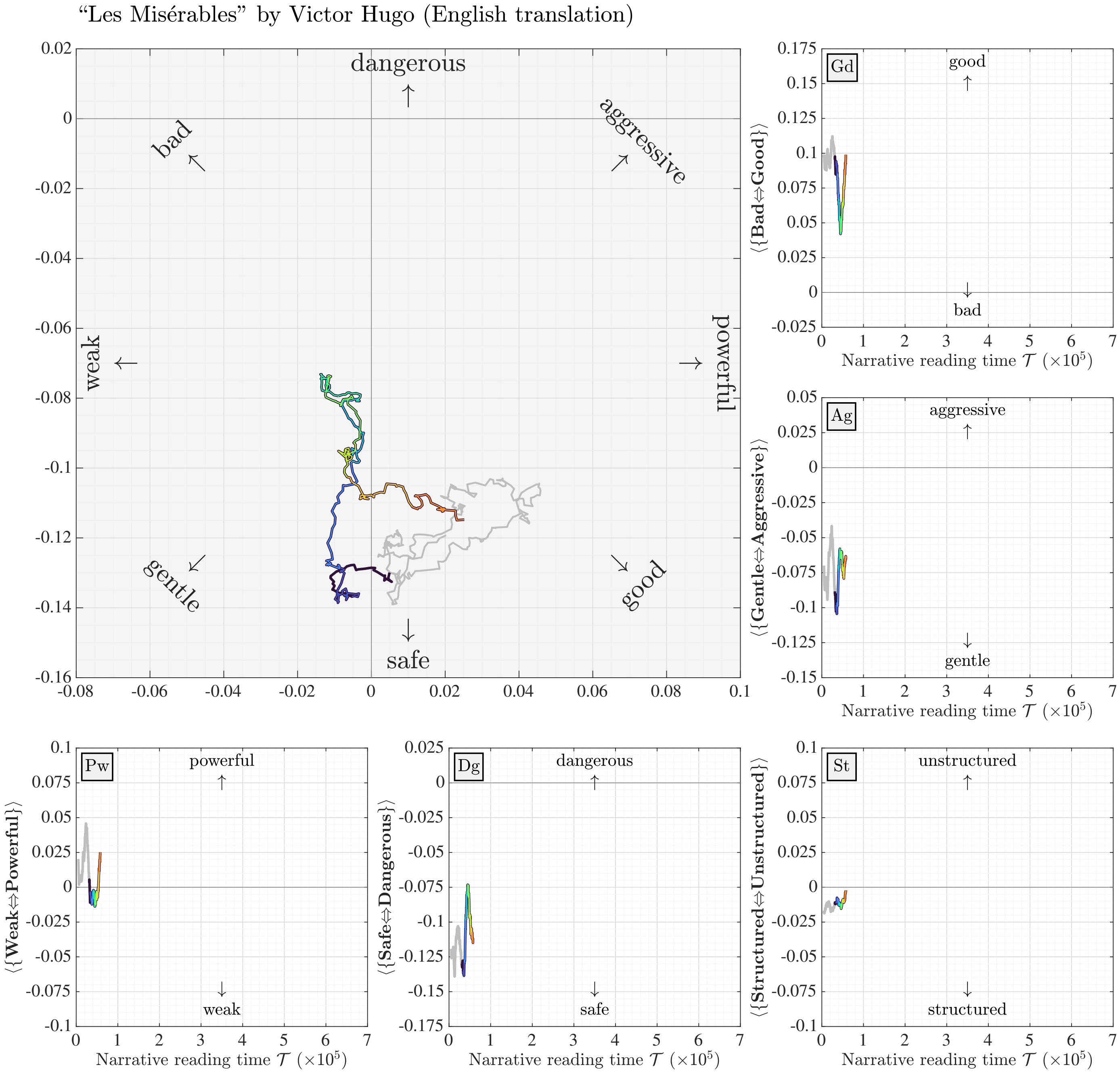

Figure S49: Epoch 2 of 25 in Victor Hugo's "Les Misérables."

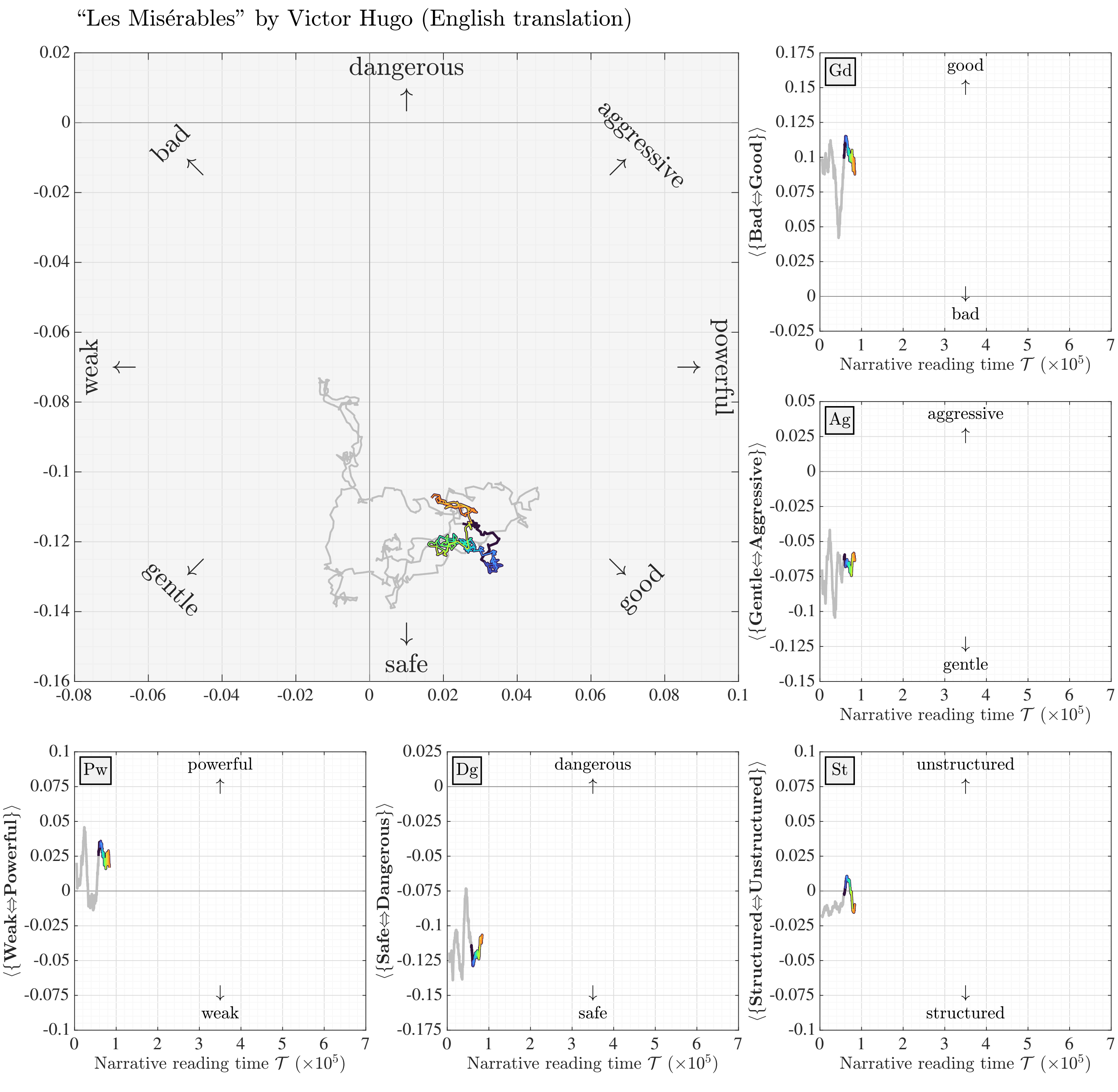

Figure S50: Epoch 3 of 25 in Victor Hugo's "Les Misérables."

“Les Misérables” by Victor Hugo (English translation)

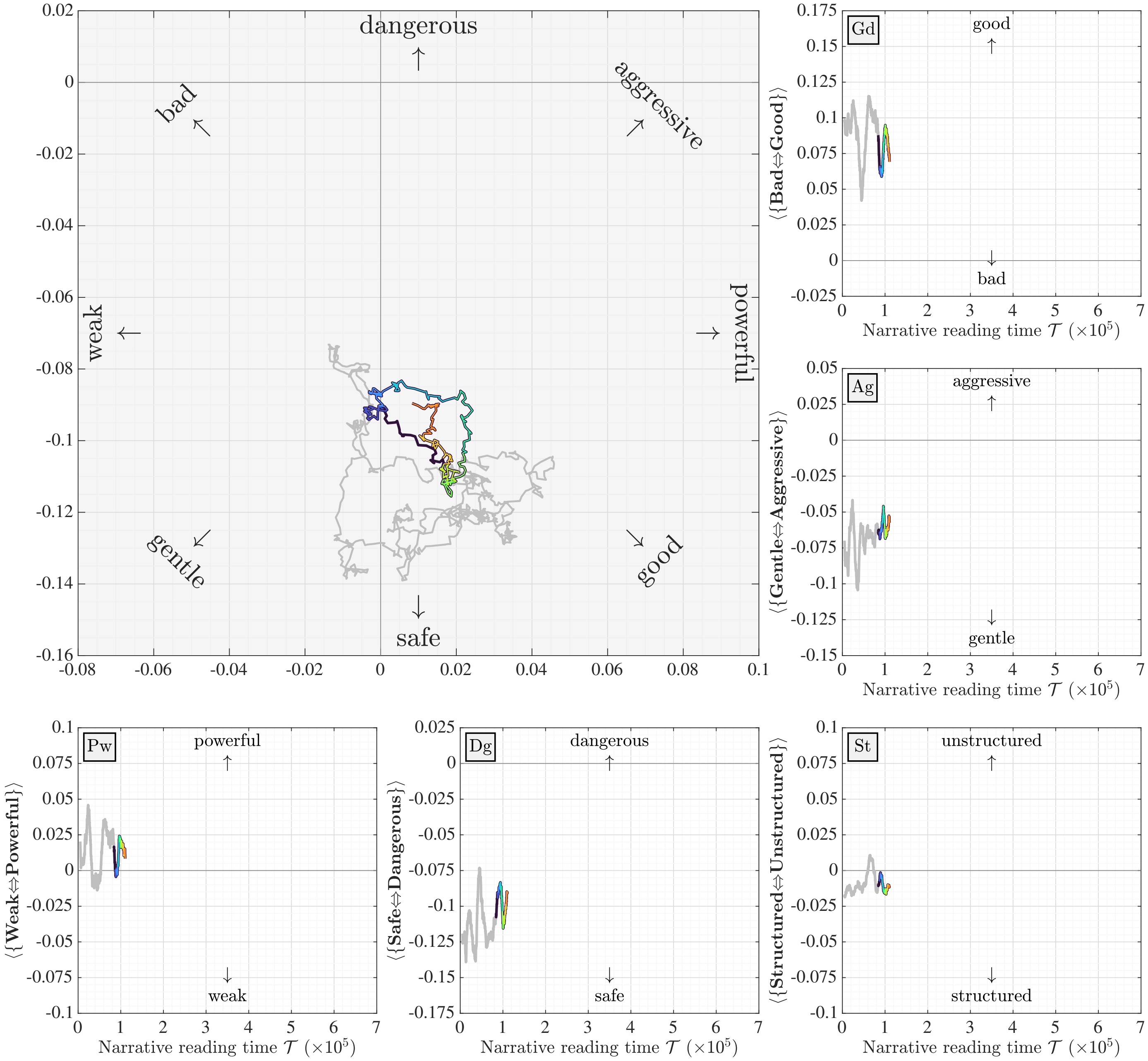

Figure S51: Epoch 4 of 25 in Victor Hugo’s “Les Misérables.”

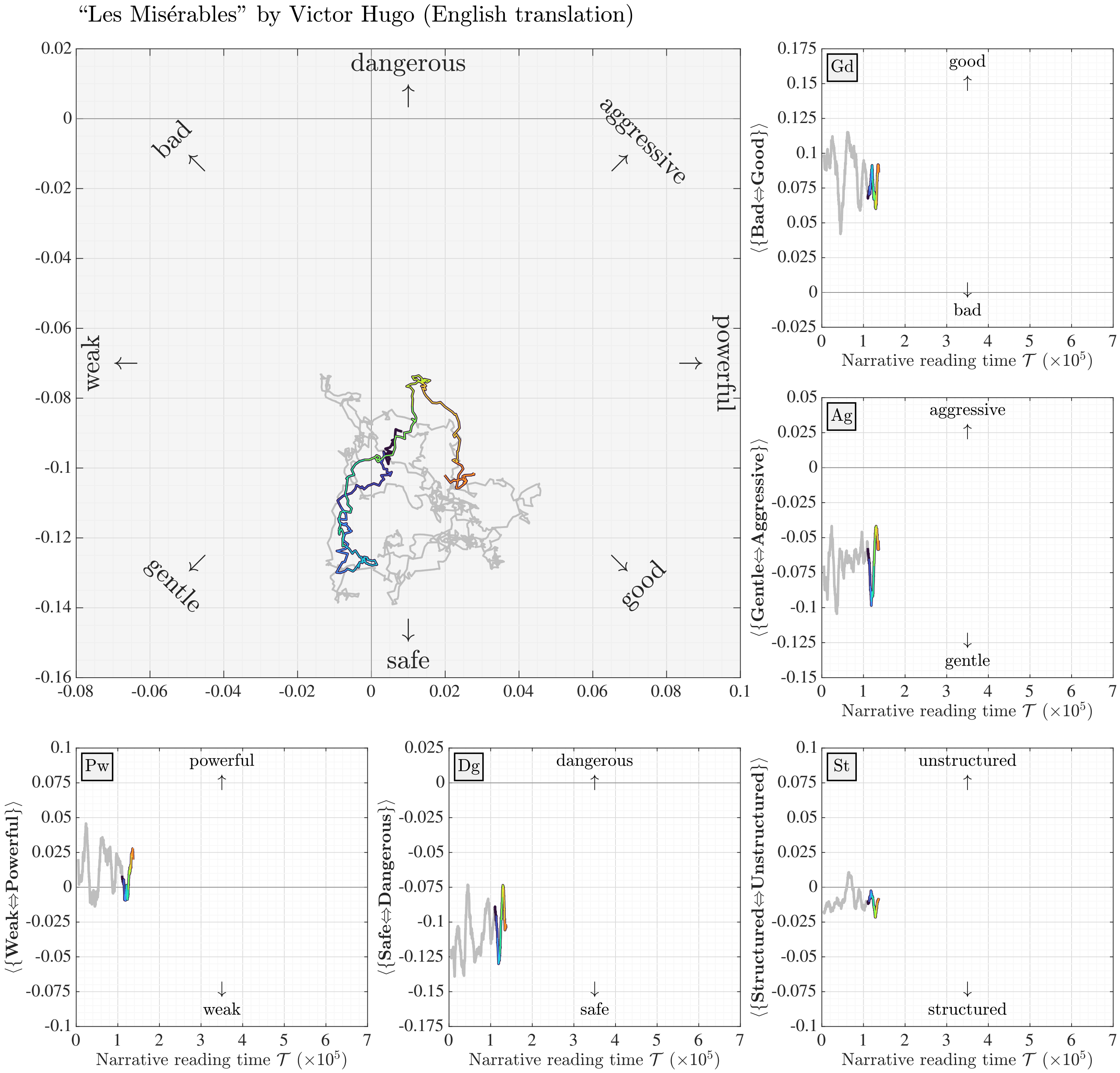

Figure S52: Epoch 5 of 25 in Victor Hugo's "Les Misérables."

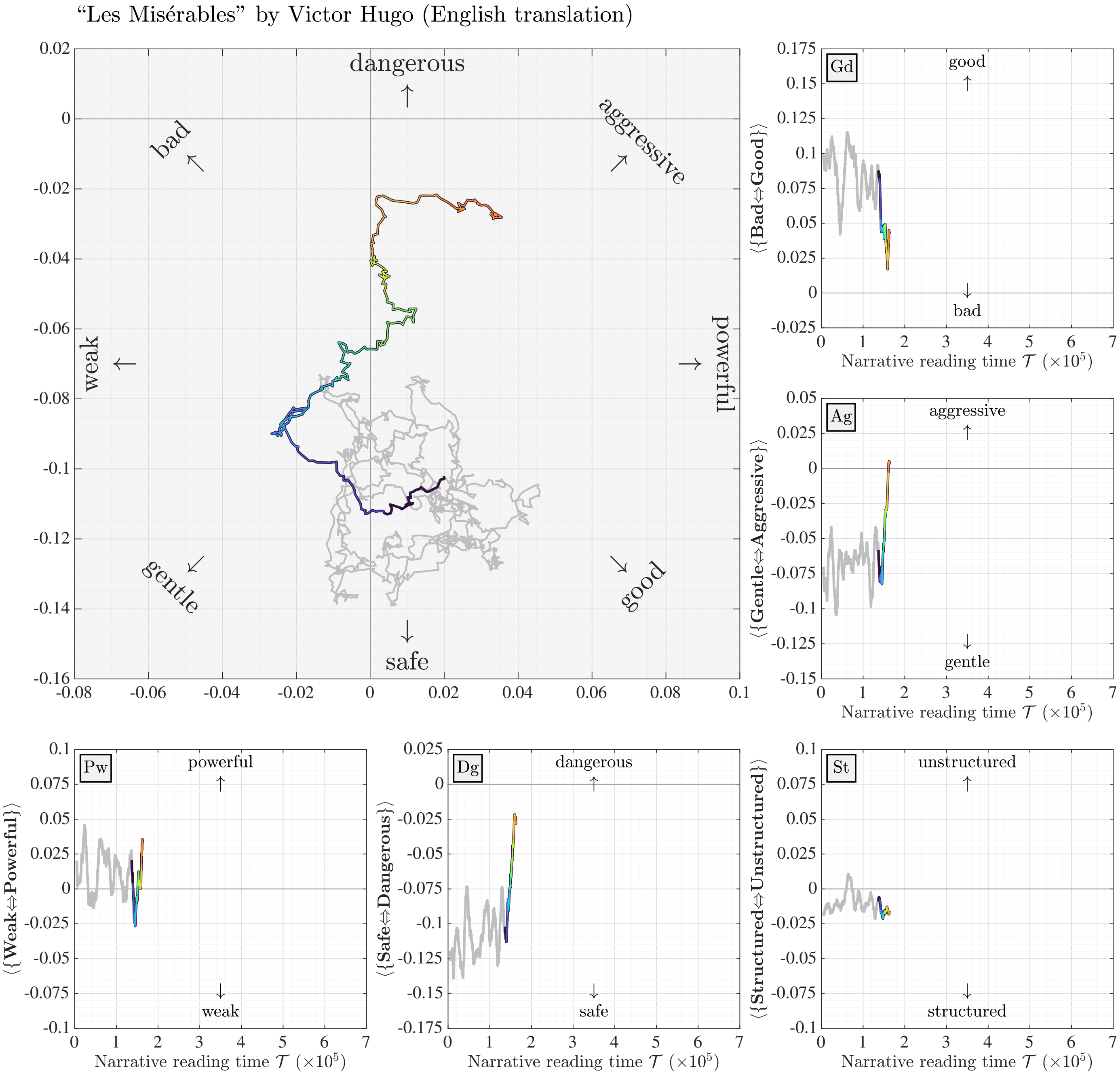

Figure S53: Epoch 6 of 25 in Victor Hugo’s “Les Misérables.”

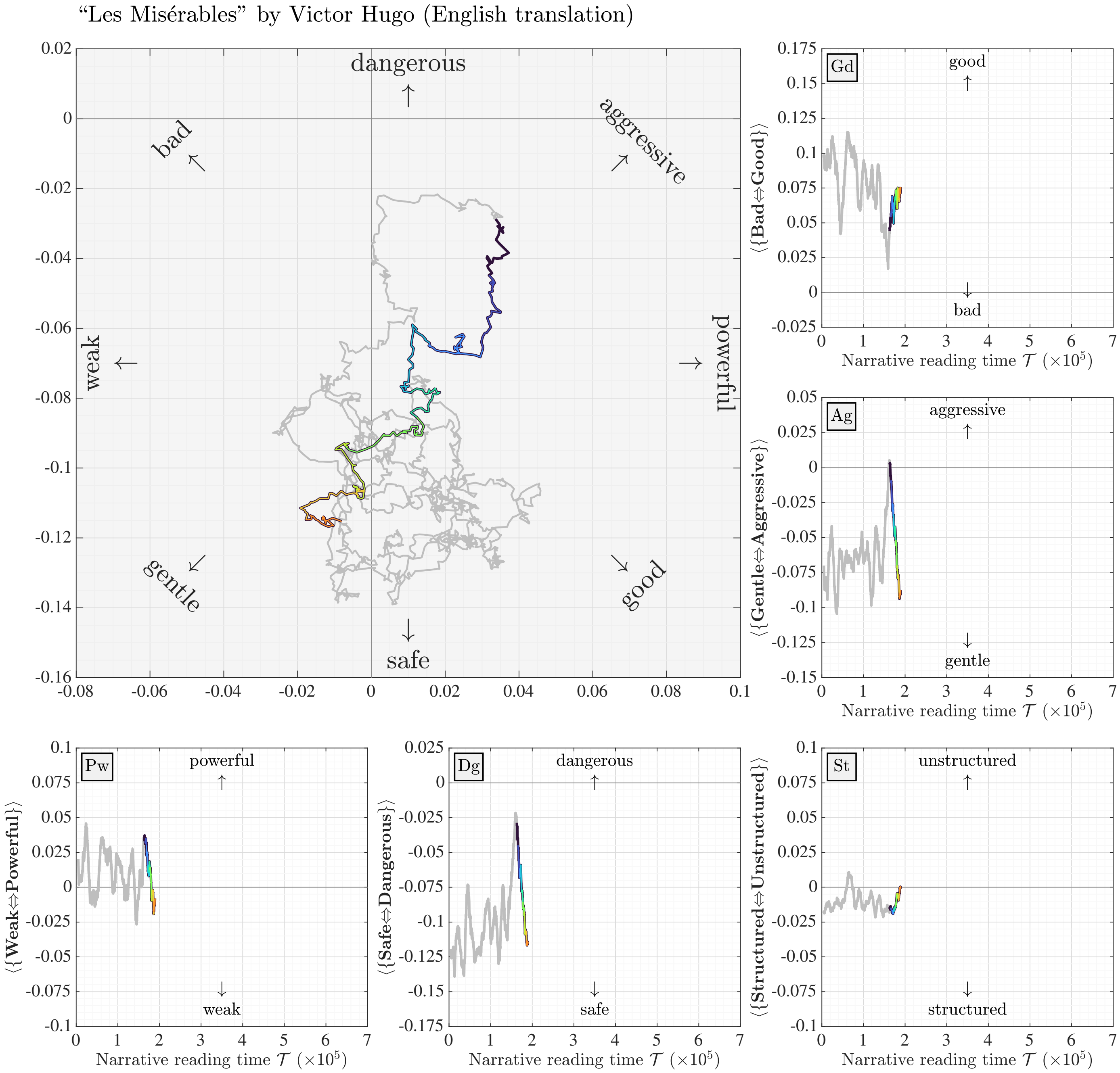

Figure S54: Epoch 7 of 25 in Victor Hugo’s “Les Misérables.”

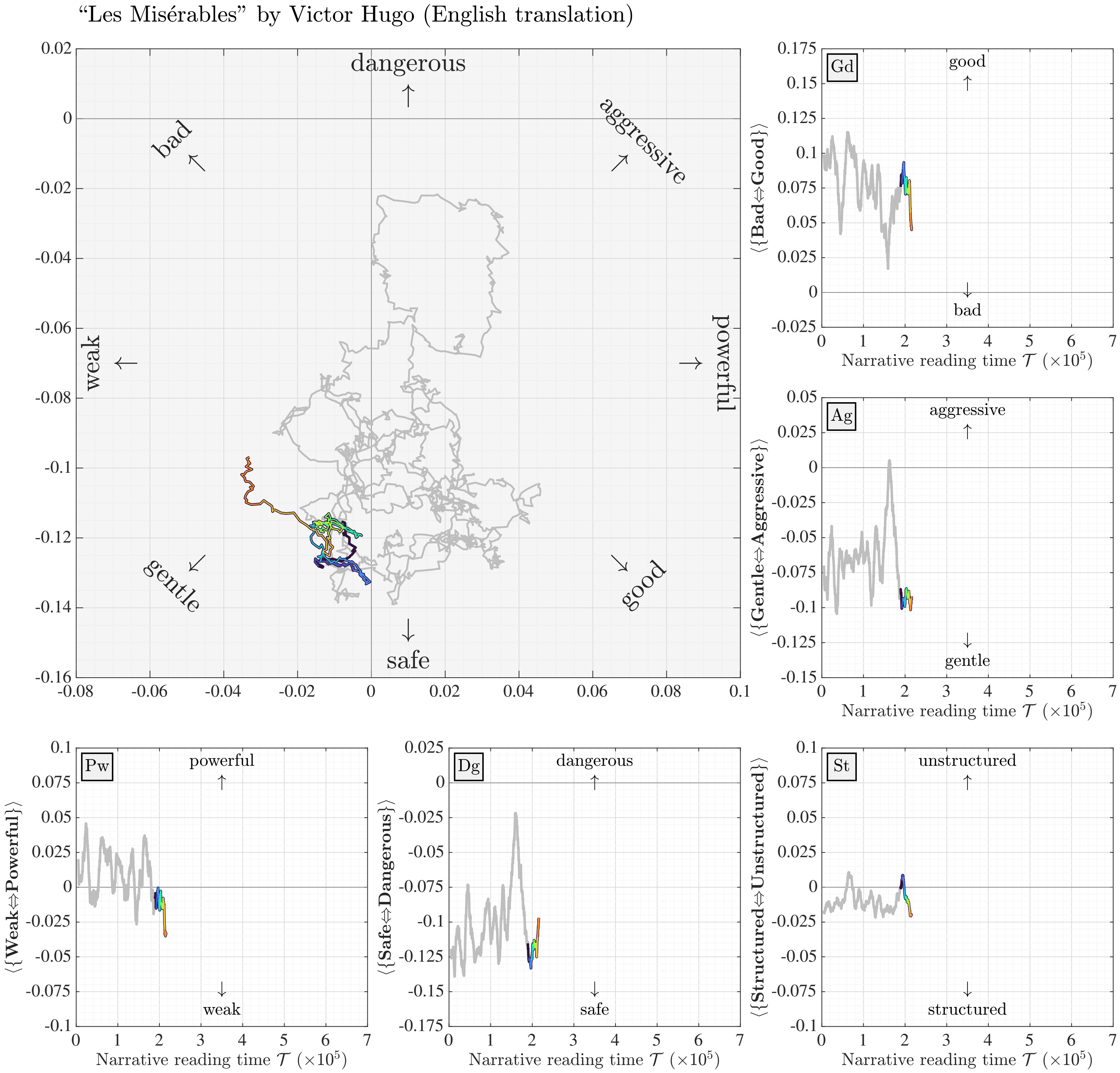

Figure S55: Epoch 8 of 25 in Victor Hugo's "Les Misérables."

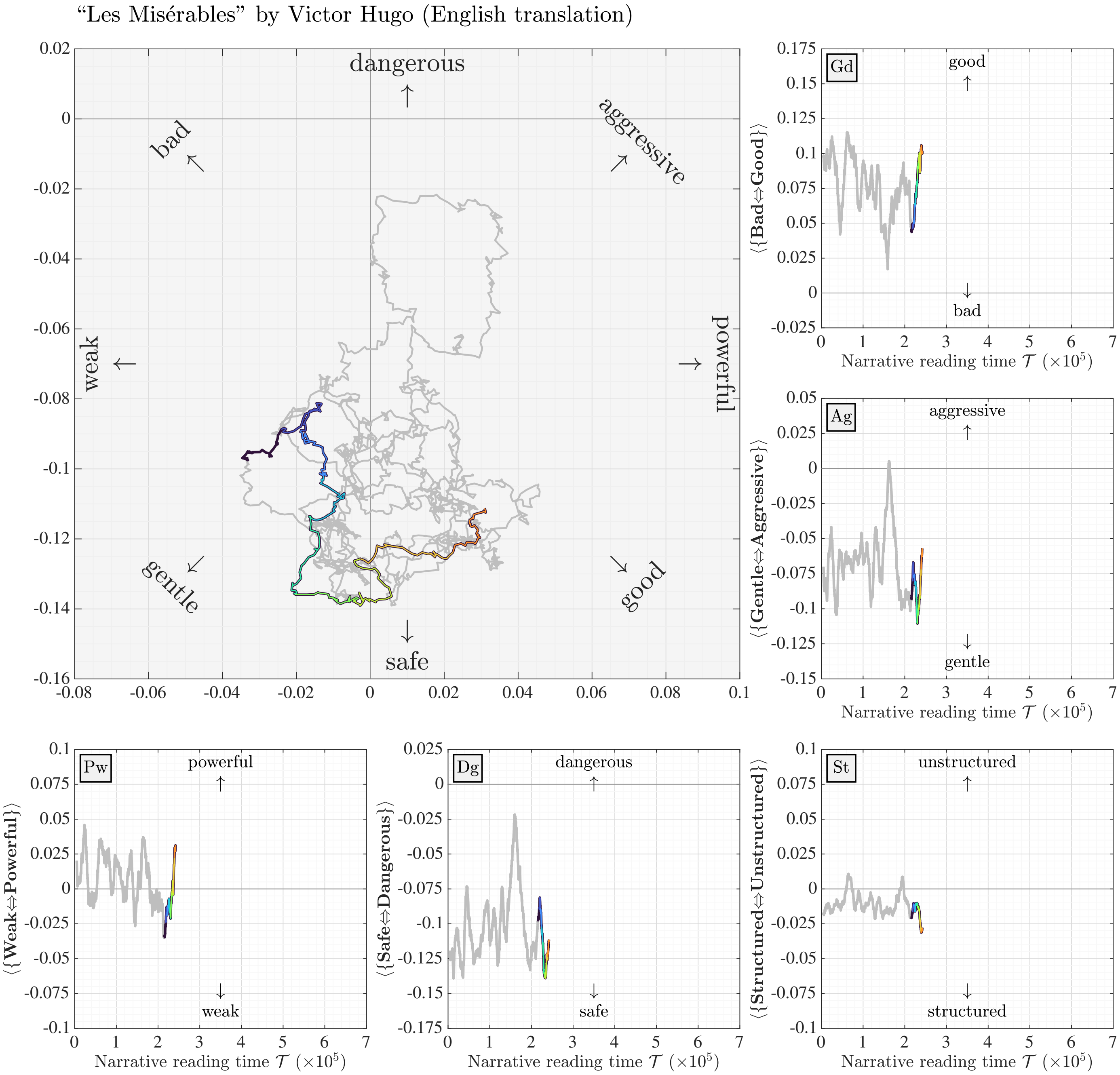

Figure S56: Epoch 9 of 25 in Victor Hugo's "Les Misérables."

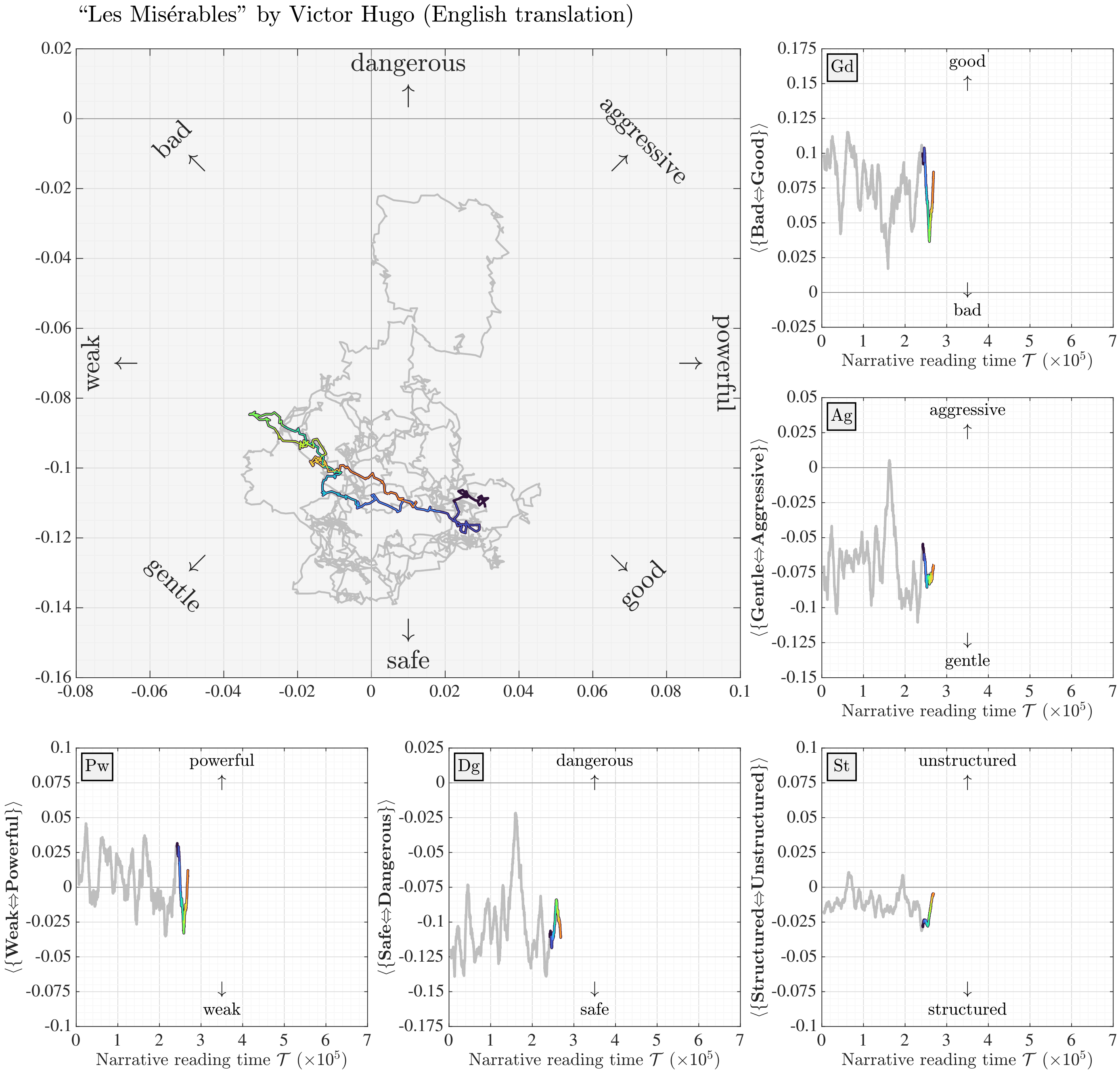

Figure S57: Epoch 10 of 25 in Victor Hugo's "Les Misérables."

“Les Misérables” by Victor Hugo (English translation)

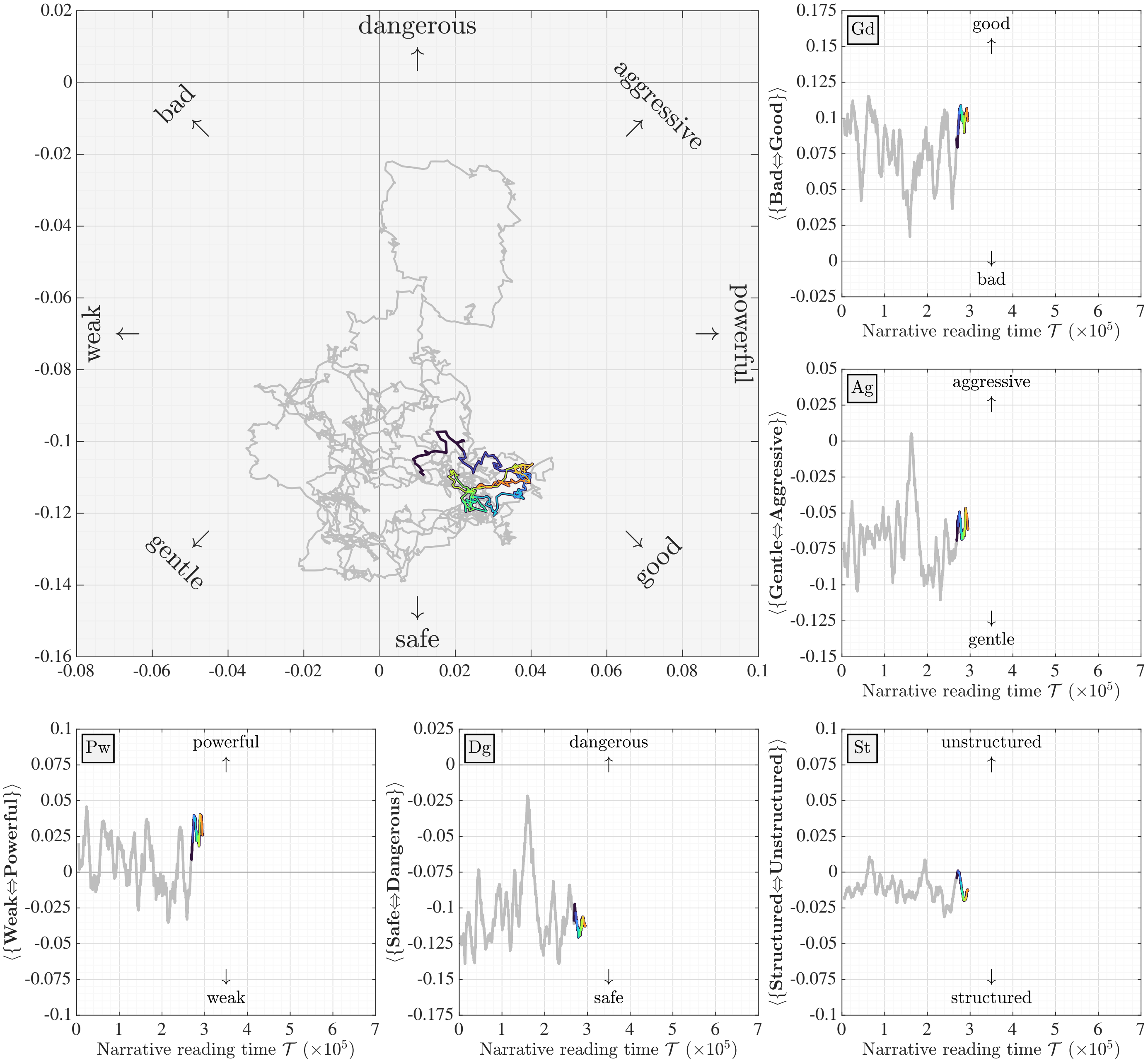

Figure S58: Epoch 11 of 25 in Victor Hugo’s “Les Misérables.”

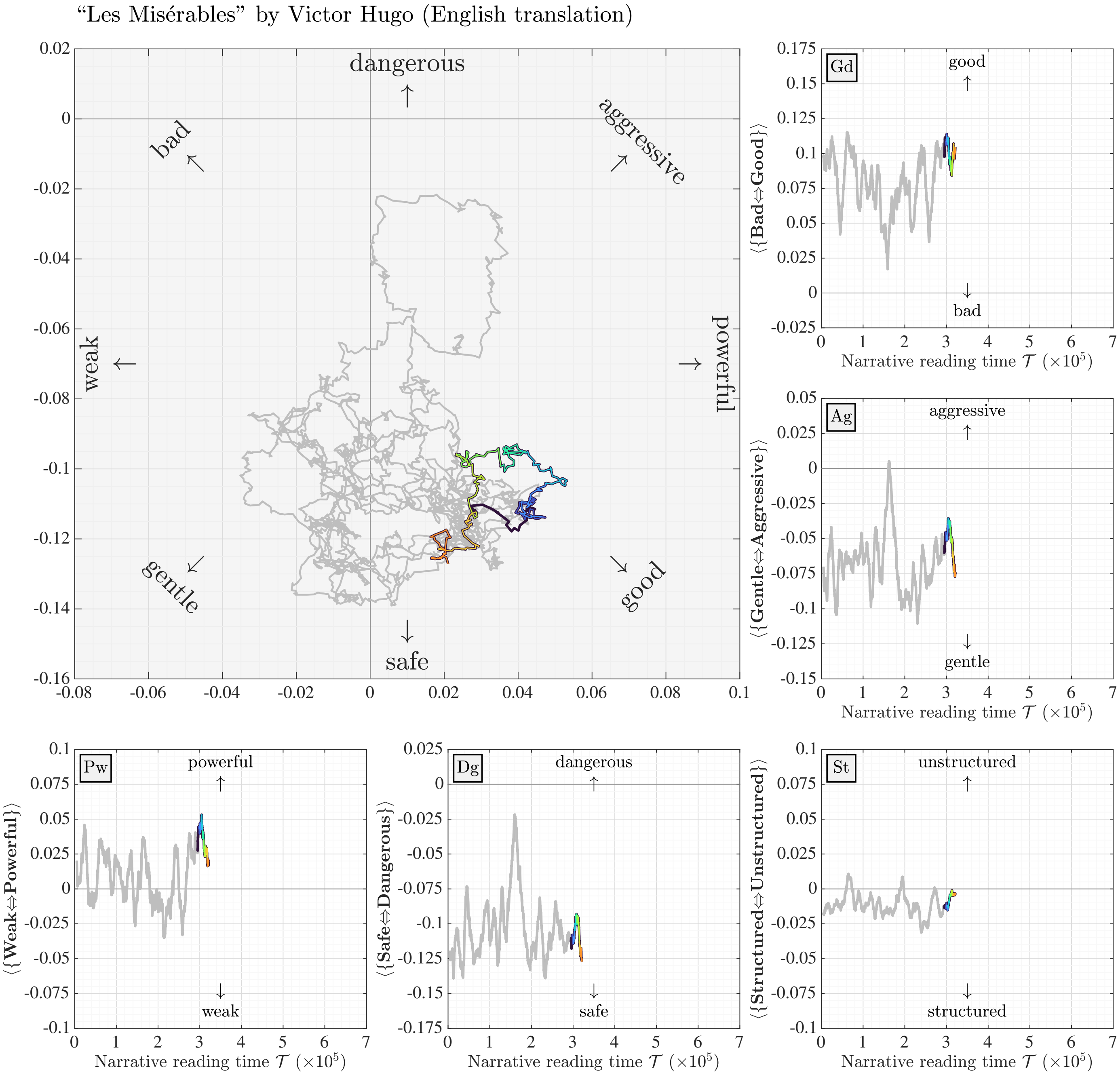

Figure S59: Epoch 12 of 25 in Victor Hugo’s “Les Misérables.”

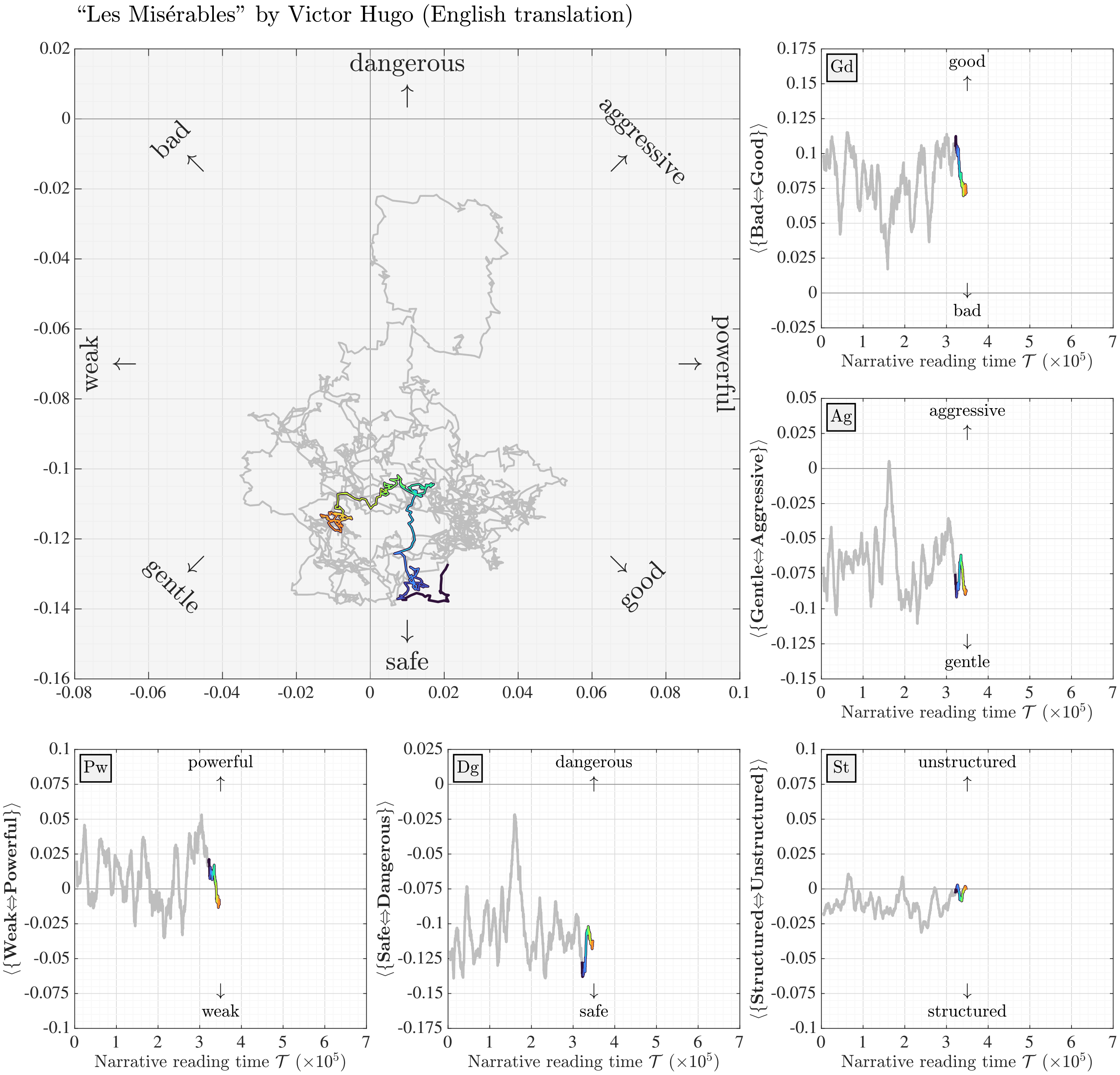

Figure S60: Epoch 13 of 25 in Victor Hugo's "Les Misérables."

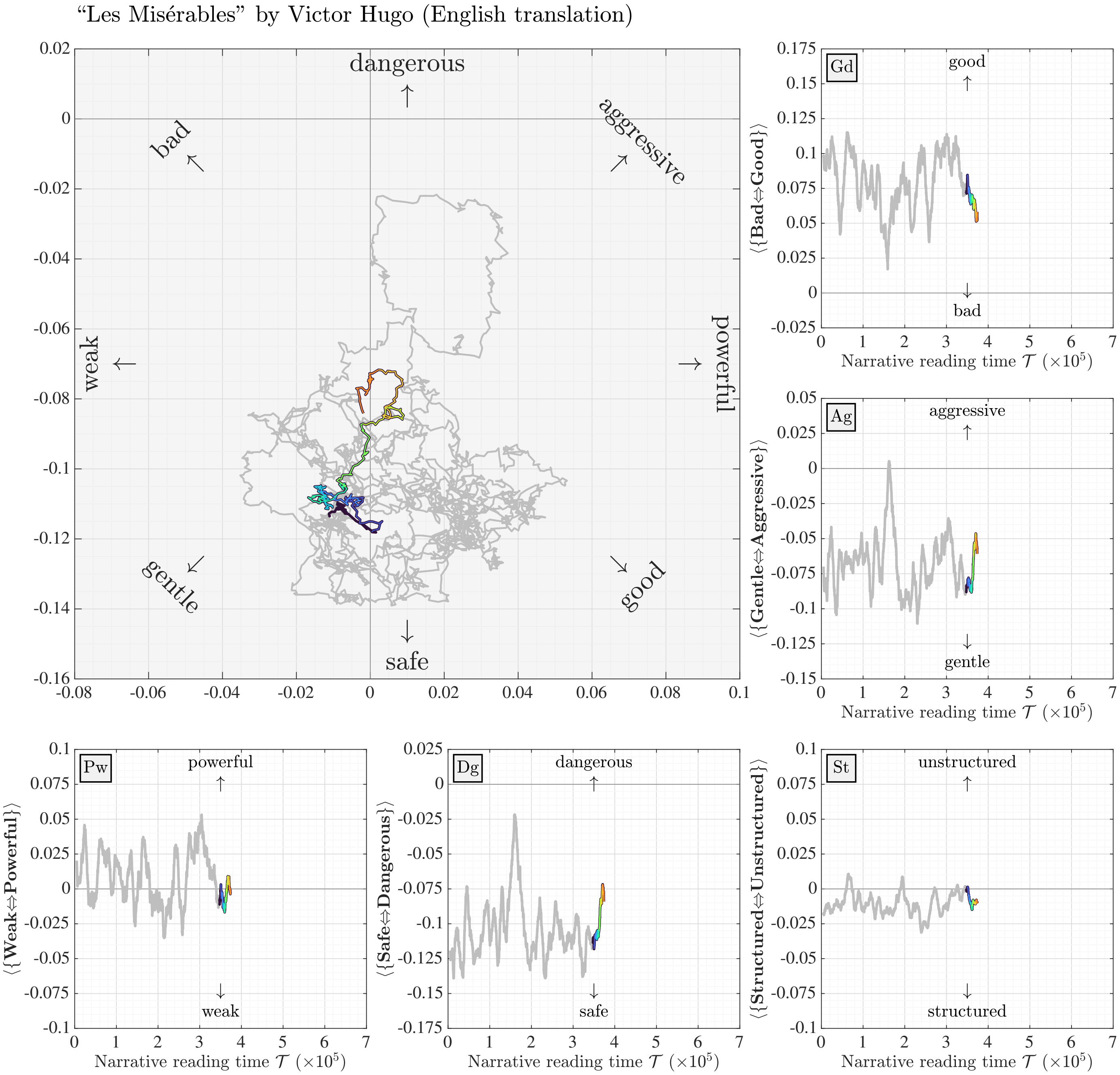

Figure S61: Epoch 14 of 25 in Victor Hugo's "Les Misérables."

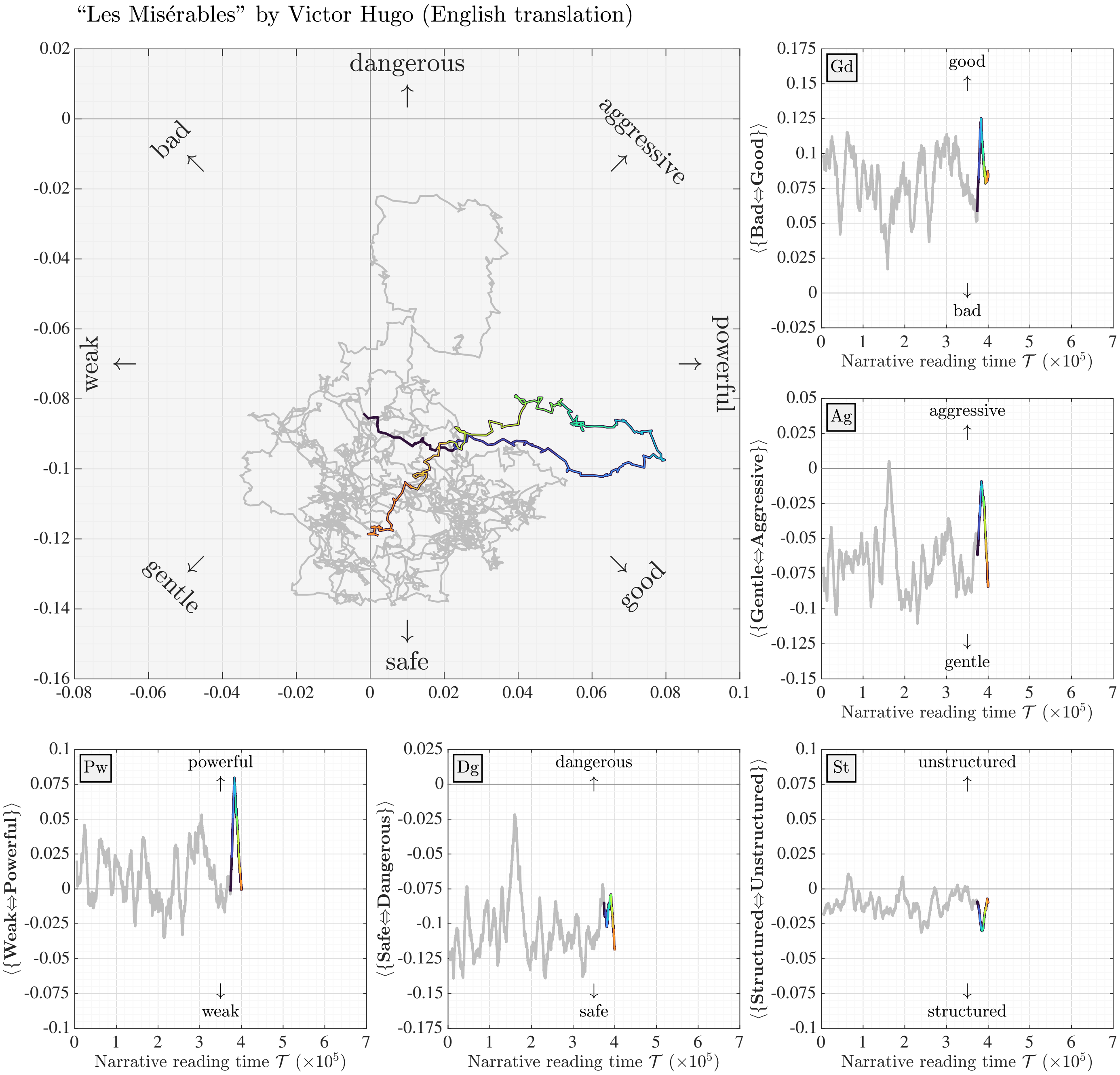

Figure S62: Epoch 15 of 25 in Victor Hugo's "Les Misérables."

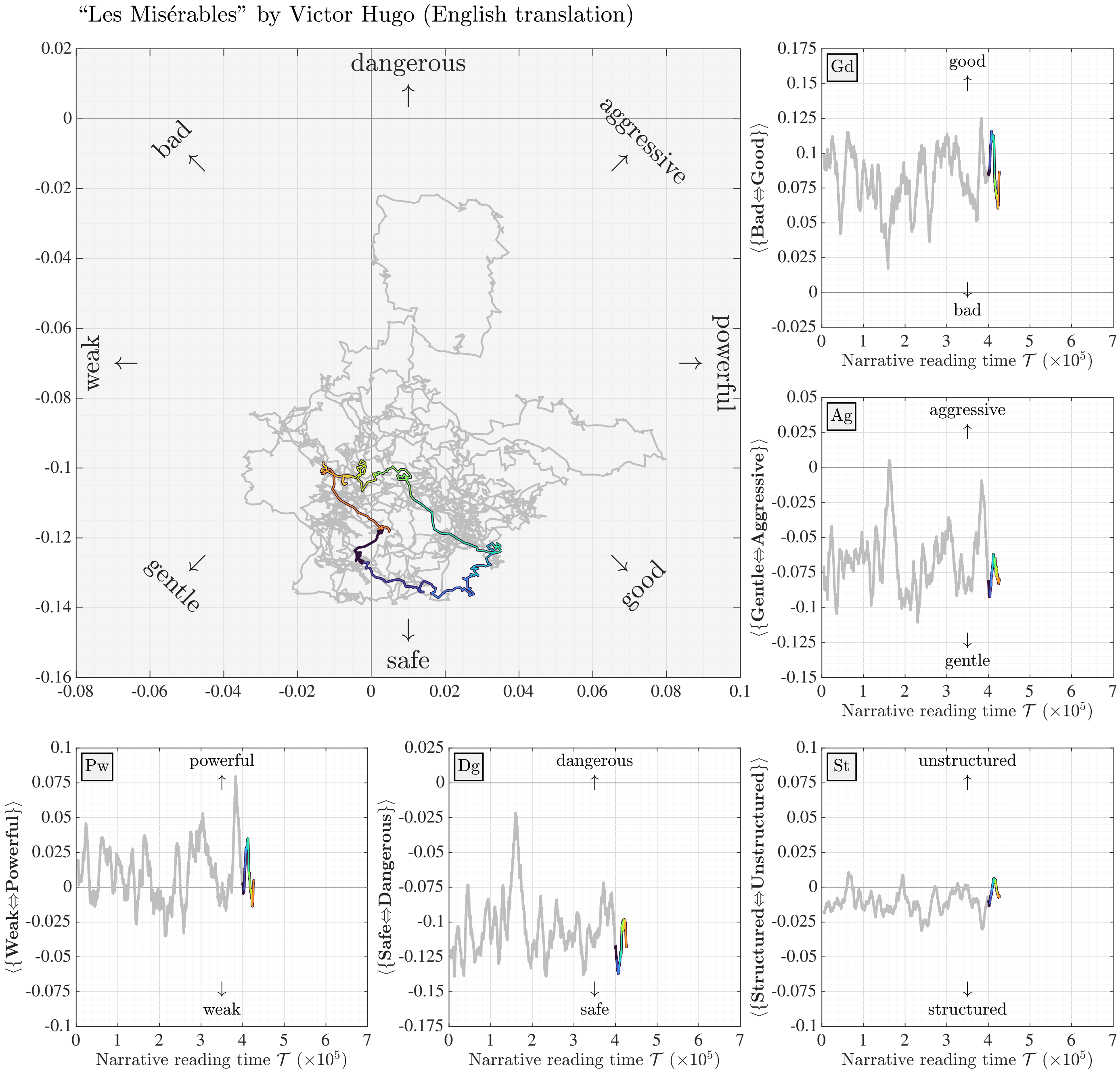

Figure S63: Epoch 16 of 25 in Victor Hugo's "Les Misérables."

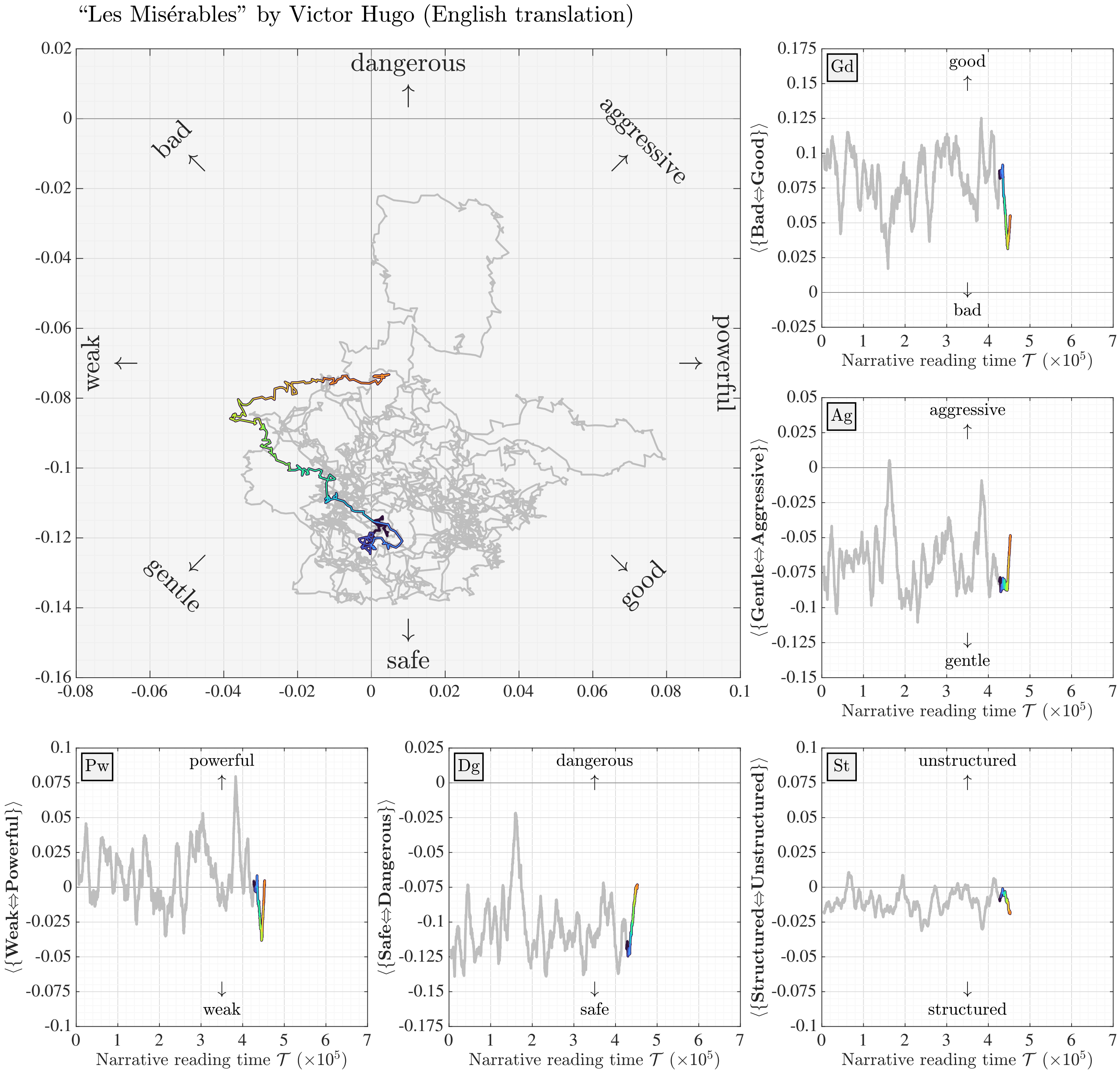

Figure S64: Epoch 17 of 25 in Victor Hugo's "Les Misérables."

“Les Misérables” by Victor Hugo (English translation)

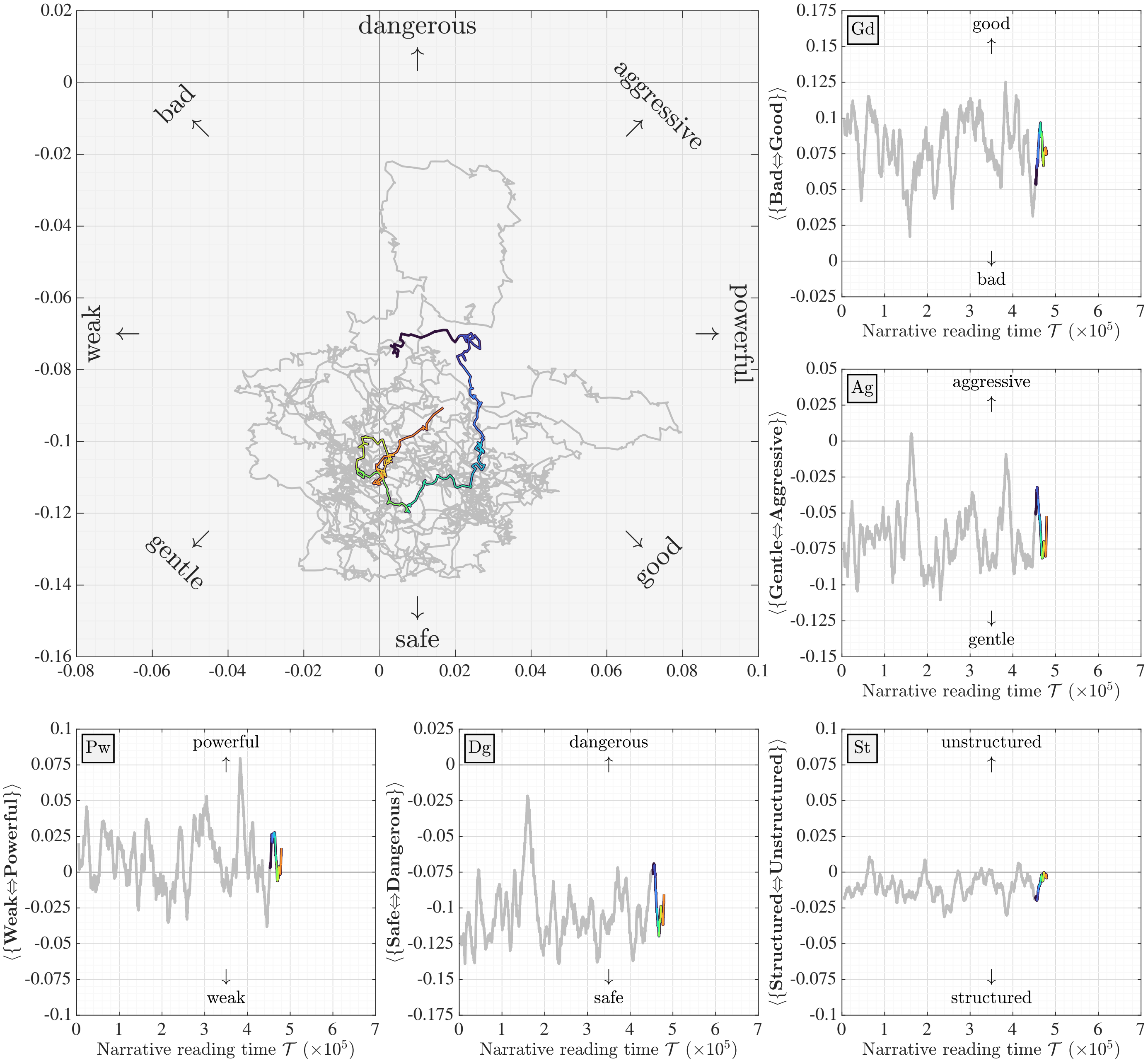

Figure S65: Epoch 18 of 25 in Victor Hugo’s “Les Misérables.”

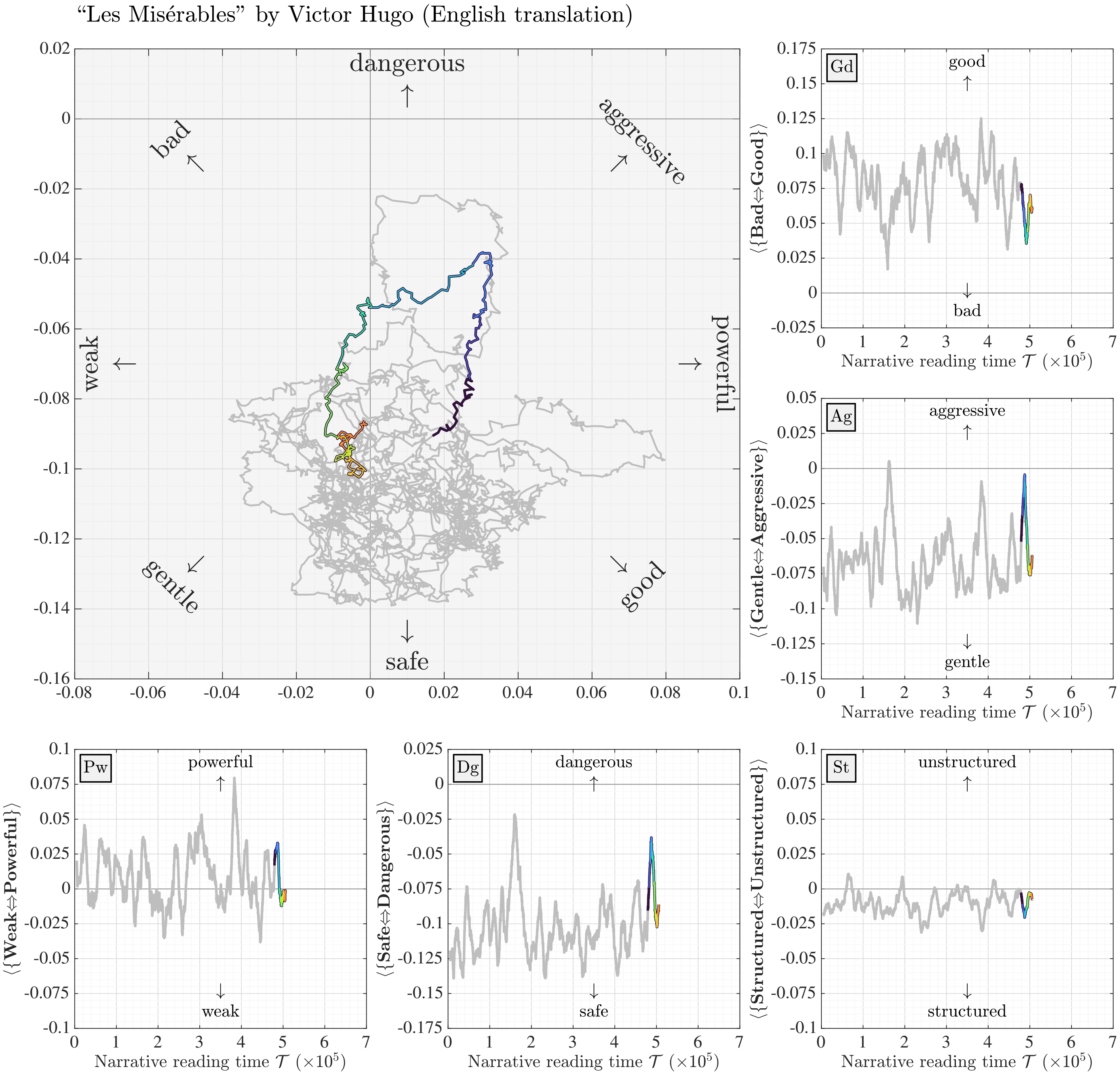

Figure S66: Epoch 19 of 25 in Victor Hugo's "Les Misérables."

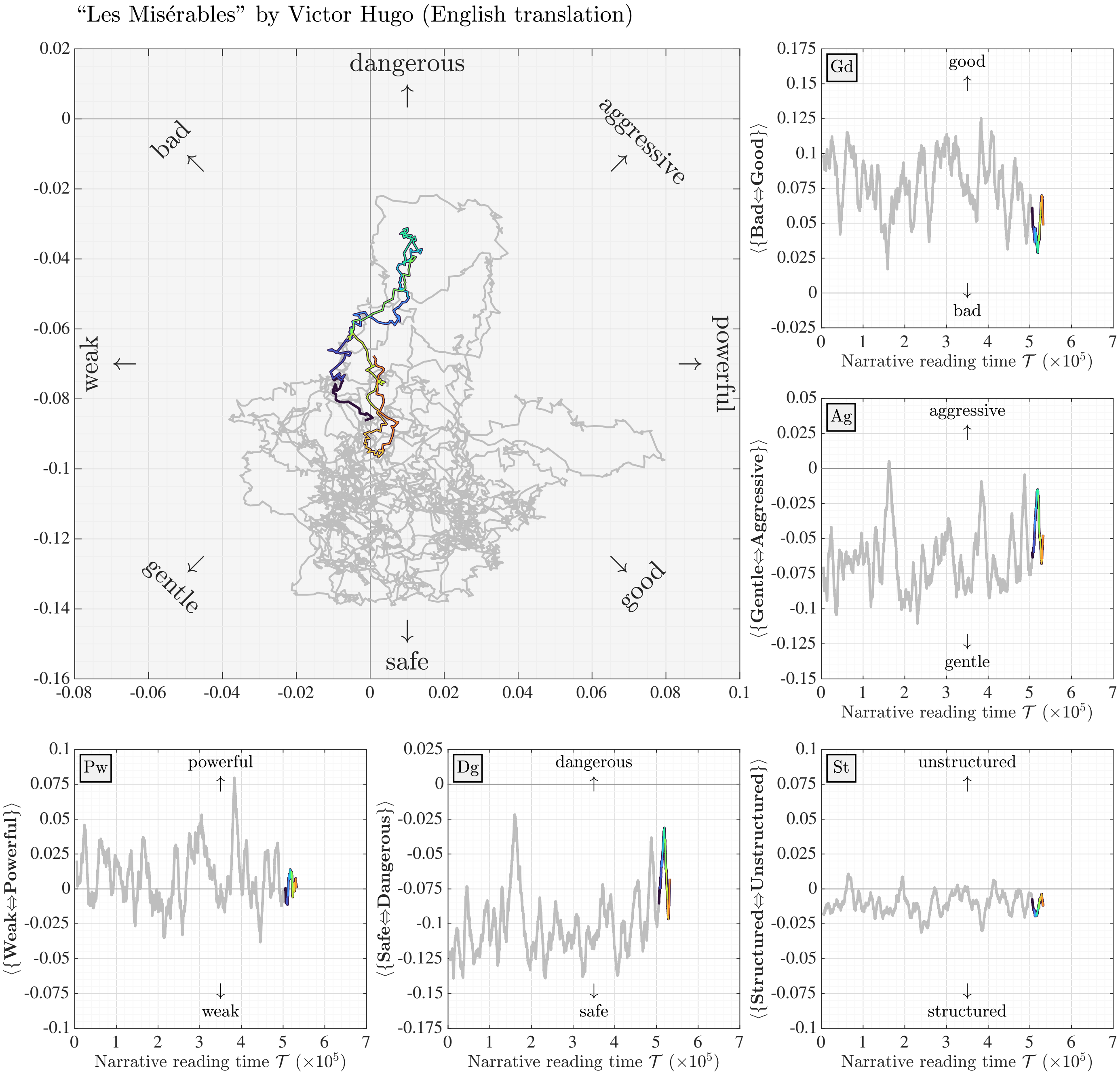

Figure S67: Epoch 20 of 25 in Victor Hugo's "Les Misérables."

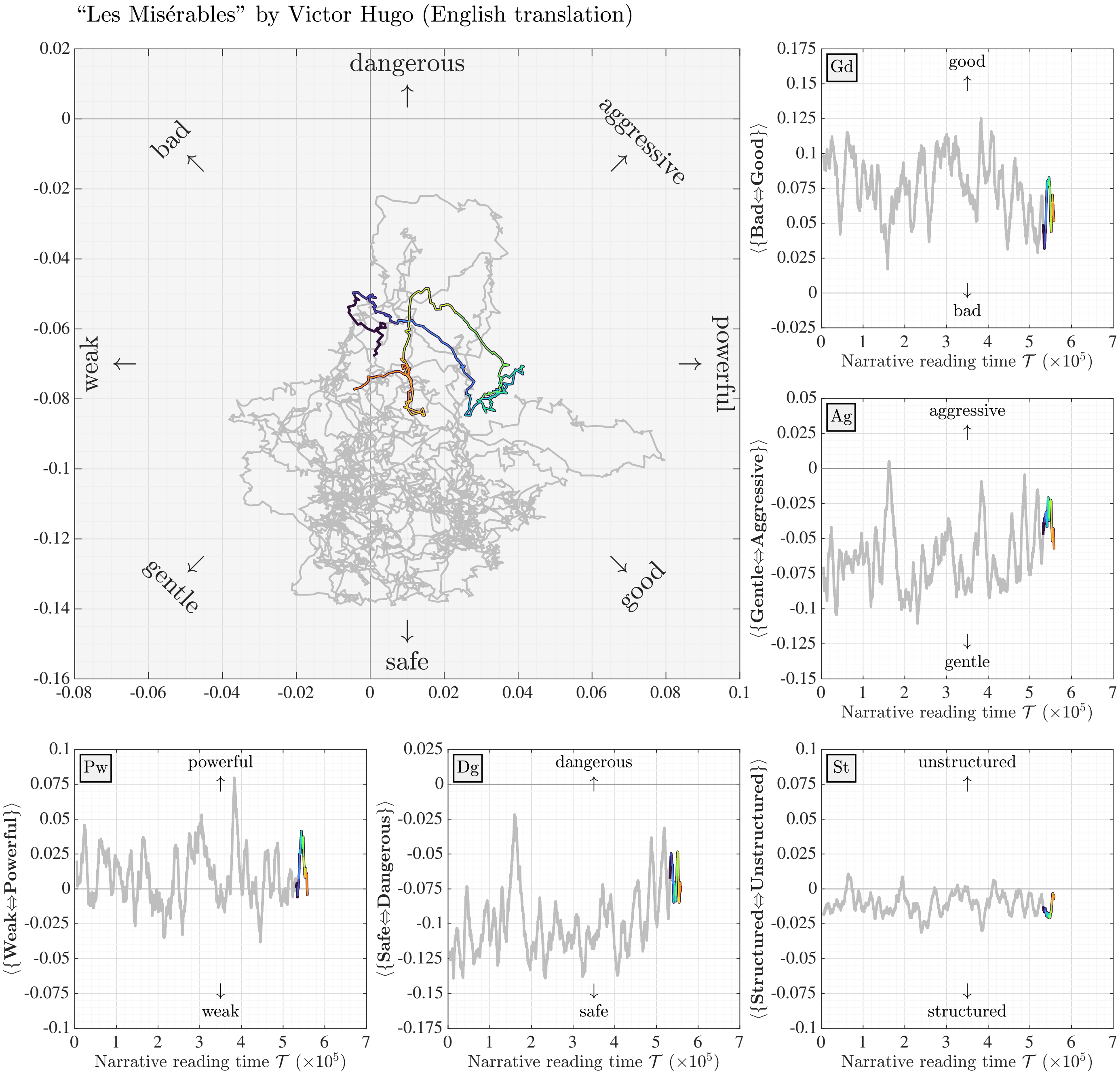

Figure S68: Epoch 21 of 25 in Victor Hugo's "Les Misérables."

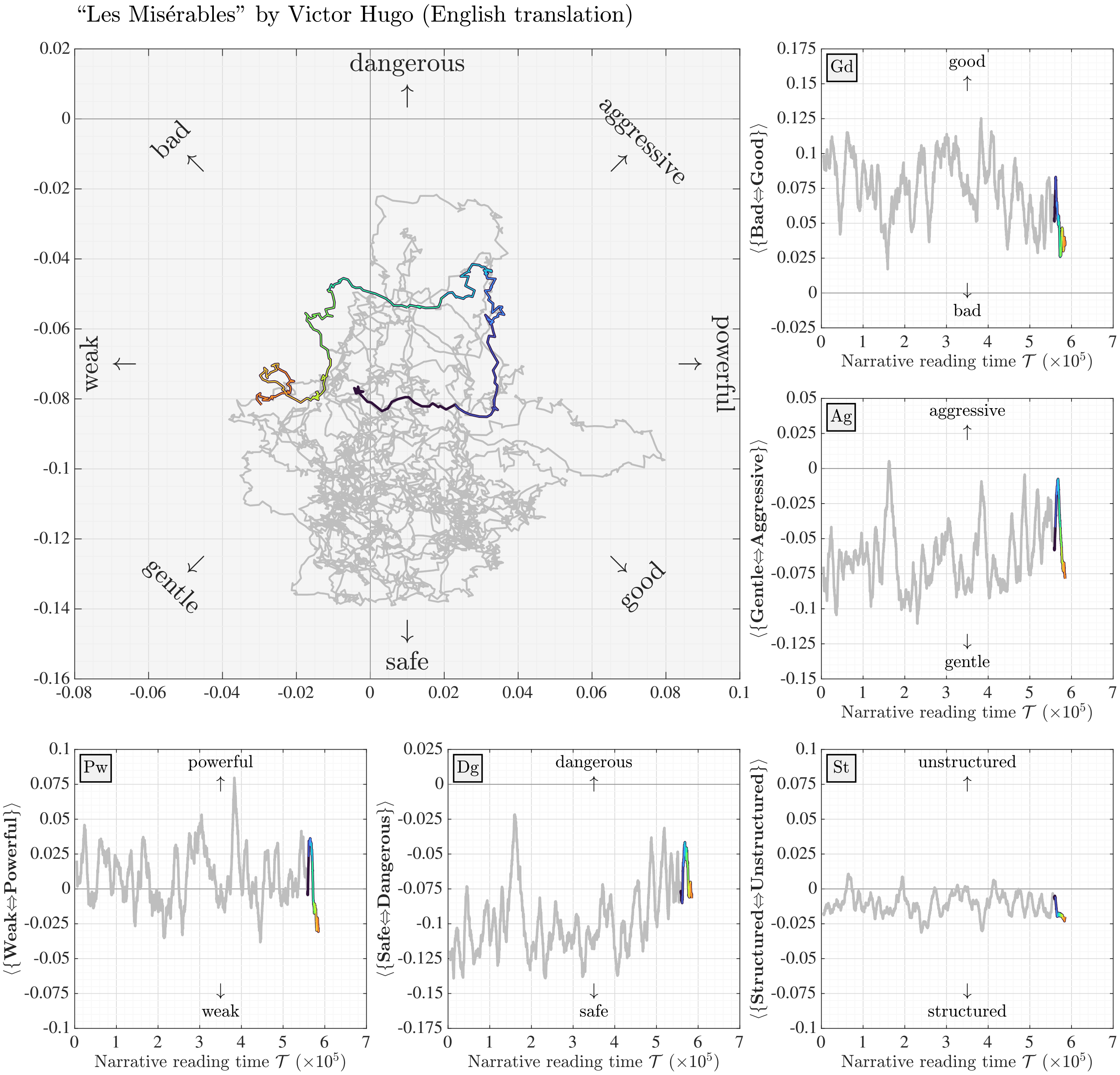

Figure S69: Epoch 22 of 25 in Victor Hugo's "Les Misérables."

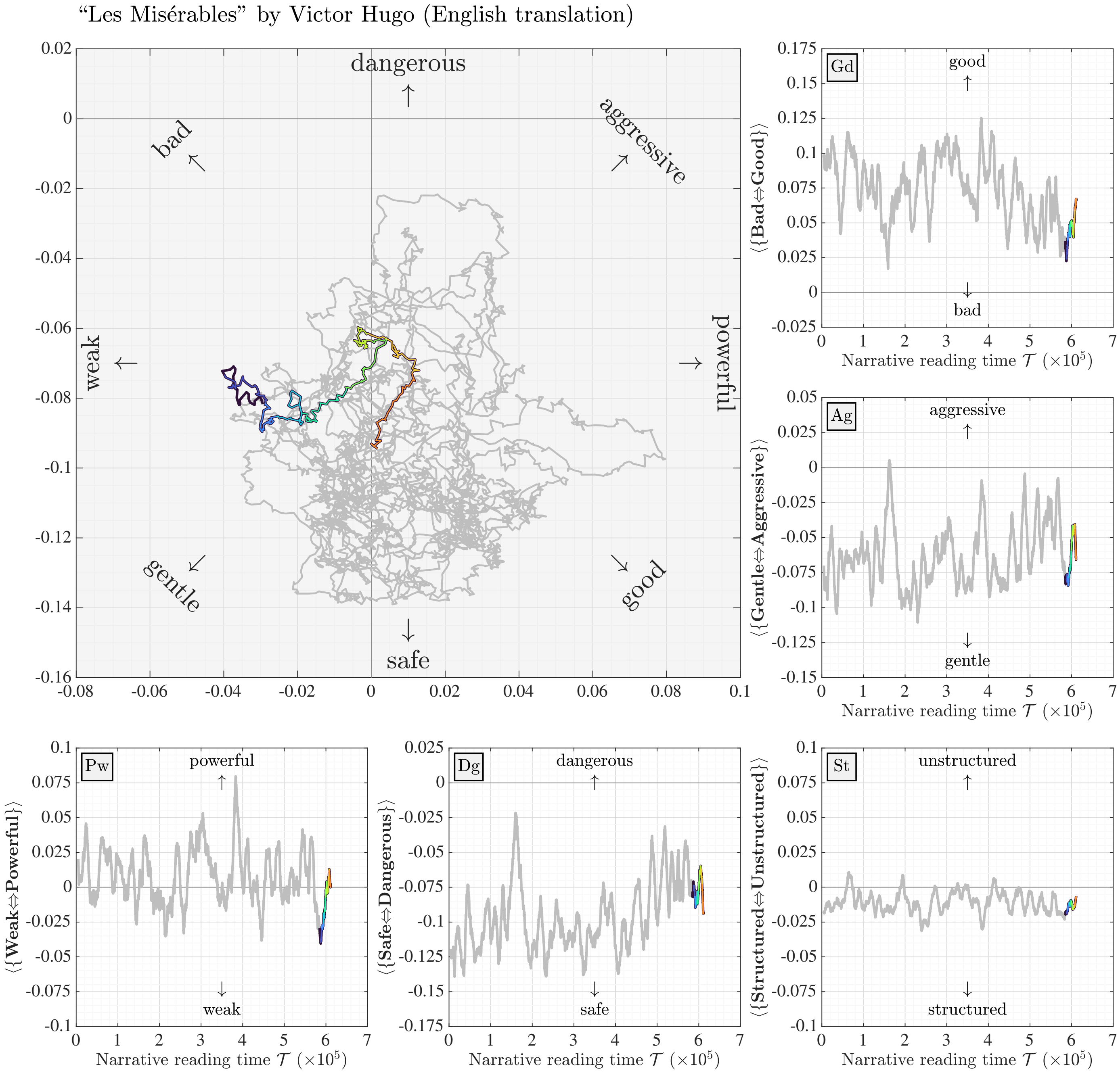

Figure S70: Epoch 23 of 25 in Victor Hugo's "Les Misérables."

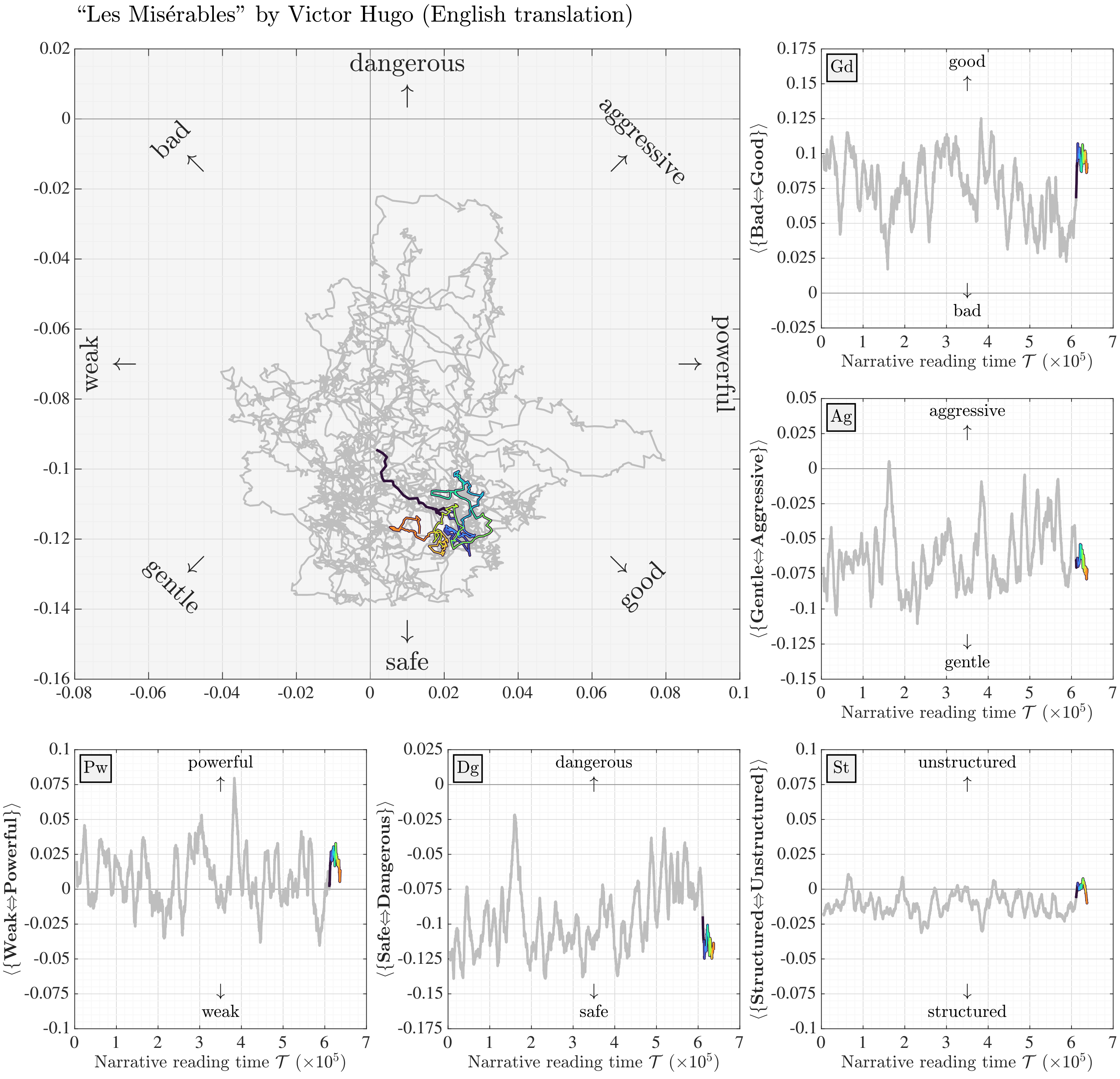

Figure S71: Epoch 24 of 25 in Victor Hugo's "Les Misérables."

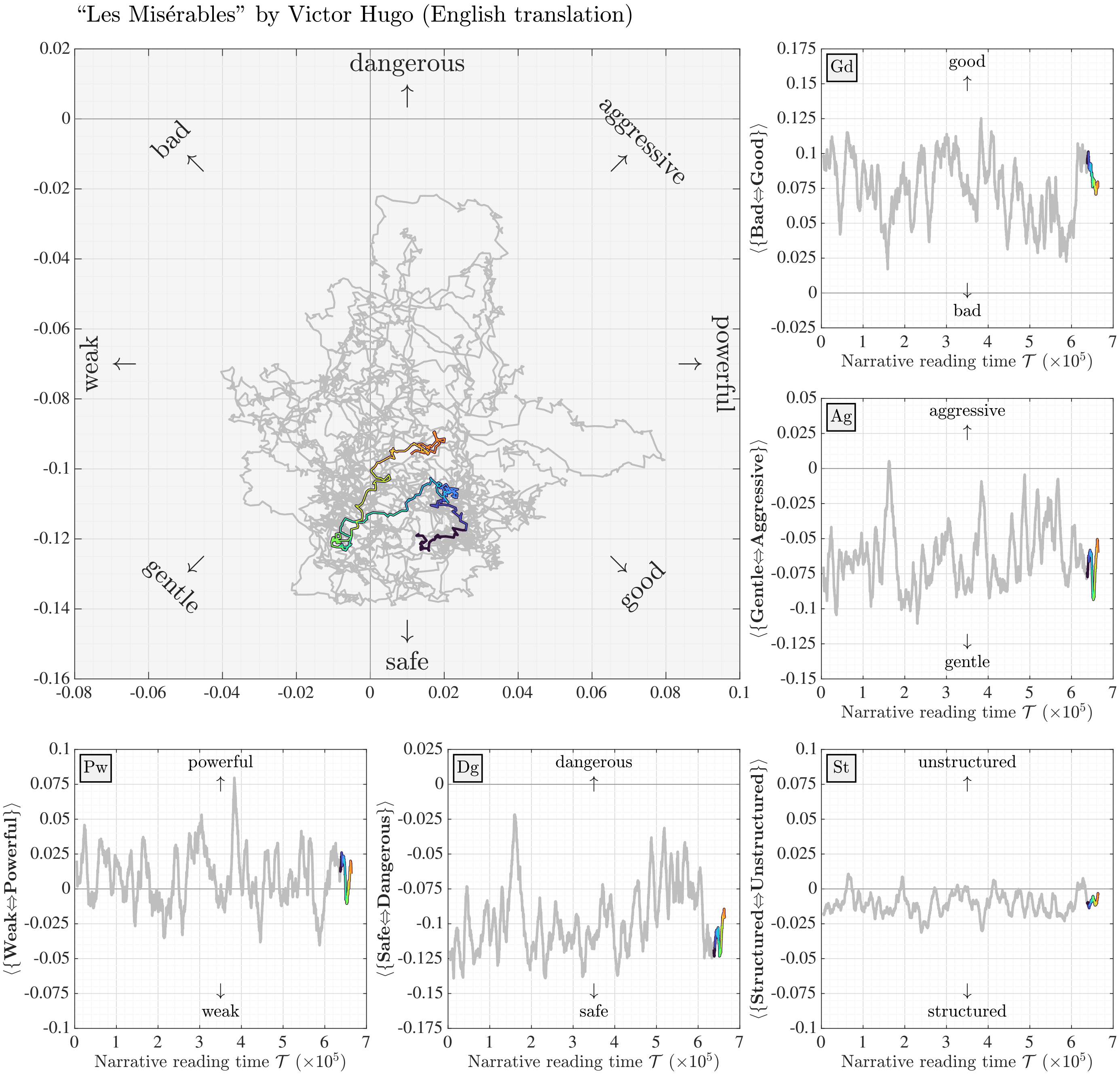

Figure S72: Epoch 25 of 25 in Victor Hugo's "Les Misérables."

S7 Hedonometer for Les Misérables

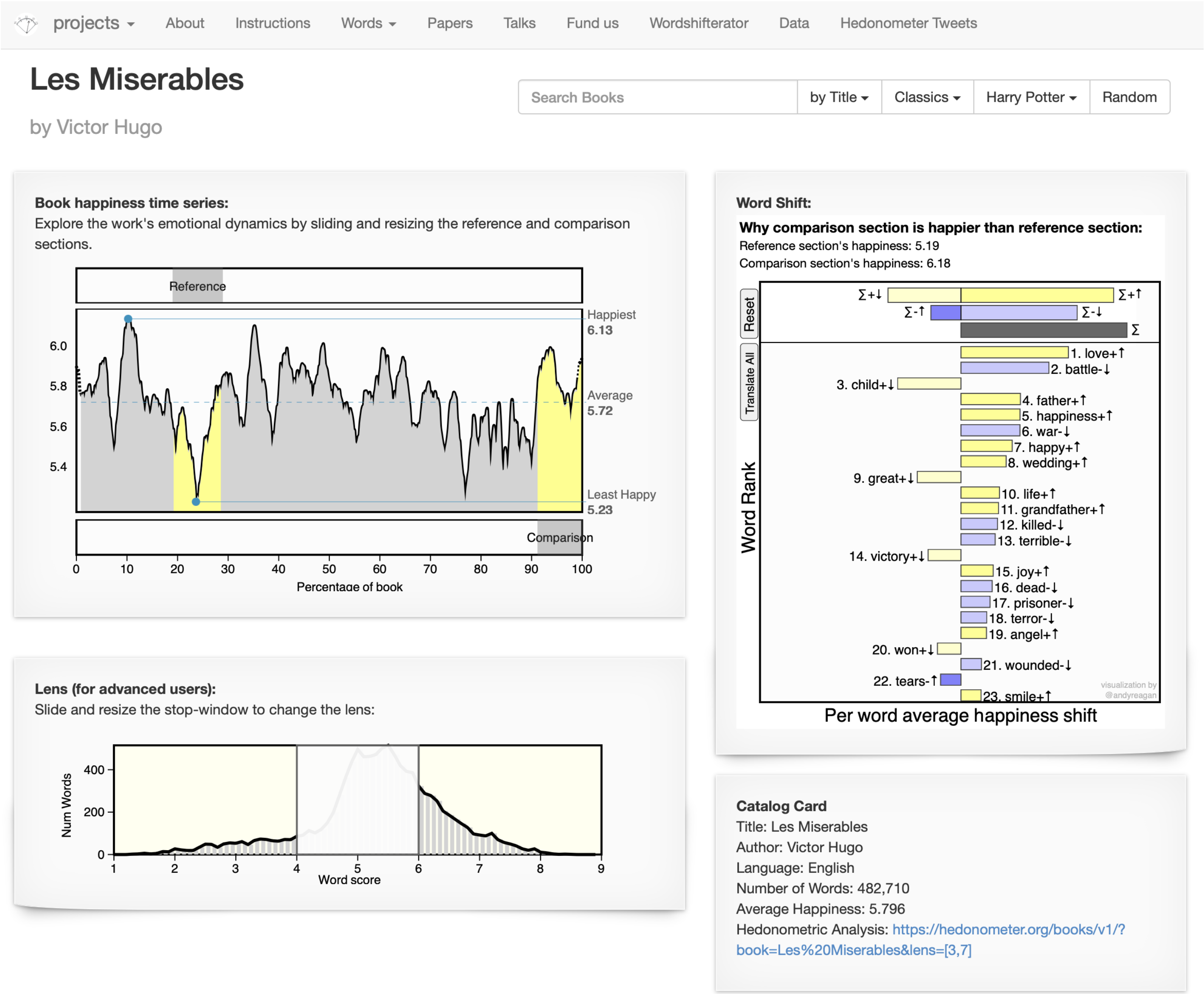

Figure S73: Hedonometric time series for Les Misérables. The time series shows a similar profile to the ousiometric {bad ⇔ good} time series in Fig. 9 in Sec. 5. Screenshot from interactive viewer at https://hedonometer.org based on Ref. [72].

S8 Ousiometer prototype for high frequency social media

For an example corpus, we assess English language Twitter [57, 65] for the historically turbulent time period 2020/01/01 to 2021/01/31 [81]. In Fig. S74, we show ousiometric time series for the three frameworks of VAD, GAS, and PDS. We explain how we compute these time series and then briefly discuss how they track specific historical events.

We now apply our prototype ousiometer to English language Twitter at a base resolution of 15 minutes [57, 65]. We use Eq. (5) to generate the ousiometric time series in Fig. S74. The three columns of Fig. S74 correspond to the VAD, GAS, and PDS frameworks. The rows from top to bottom move from the year scale of 2020 and the start of 2021, focusing in on the attack on the US Capitol by supporters of President Trump on 2021/01/06. The specific time frames are 13 months (2020/01/01 to 2021/01/31), 5 weeks (2020/12/19 to 2021/01/23), and 3 days (2021/01/05 to 2021/01/07). We overlay day-scale and hour-scale smoothing for the first two rows respectively.

Looking across all panels, we see the various ousiometric biases in the context of Twitter. Valence, dominance, goodness, and power all show positive biases, while arousal, aggression, and danger present negative averages. Structure is the only neutral dimension.

At the year scale in Figs. S74A–C, the three frameworks show evidence of major shocks, trends, and daily fluctuations, all to varying degrees. The two major events in the first half of 2020—those leading to long-lasting societal effects—were the global realization of the COVID-19 pandemic in mid March and the murder of George Floyd at the end of May and subsequent Black Lives Matter protests [80]. A number of other events also stand out including the assassination of the Iranian general Soleimani by the US on 2020/01/03, which led to talk of war.

We only see the COVID-19-response shock in four dimensions—valence, goodness, power, and danger—while the shock of George Floyd's murder registers in all eight dimensions. The COVID-19 shock is muted in part because we are (understandably) missing key words in the NRC VAD lens such as 'coronavirus', and 'covid'. The word 'pandemic' points directly to danger with PDS scores (0.00,0.45,-0.03), as does 'virus' with (-0.04, 0.32, 0.06). As we discuss below, expanding the NRC VAD lexicon is an evidently needed step for improving the ousiometer.

Moving to the five weeks of the second row of Fig. S74, the main signal deviations are due to Christmas, New Year's Eve and Day, and the 2021/01/06 attack on the US Capitol. We also now see a daily cycle across all dimensions, reminiscent of what we found when measuring happiness (valence) on Twitter using the hedonometer [14, 76].

Finally, the time series in the bottom row of Fig. S74 show, in high temporal resolution, the collective shock expressed on Twitter in response to the attack on the US Capitol. For over roughly two hours starting after midday on 2021/01/06, we see the strongest shocks occur in valence (decreasing, Fig. S74G) and danger (increasing, Fig. S74I).

For the main dimensions of the orthogonal frameworks, GAS and PDS, it is danger **Dg** that is the real dimension of change. In the PDS framework, while danger rises, power **Pw** remains relatively constant throughout the attack. In the GAS framework, the time series for goodness and aggression mirror each other and are projections of the danger signal. We also observe an increase in more rigid and serious 1-grams, as the structure score **St** drops through the attack.

While we have presented the GAS and PDS time series as distinct sets and notwithstanding that they are of course linear transformations of each other, we suggest that showing all five time series is of value. The eight cardinal and intercardinal points of the power-danger plane are all meaningful, and it is helpful to reflect on which one might be dominating. We are after all plotting time series that represent the harder-to-visualize trajectory of a curve in PDS space.

For a deeper analysis of all time series, and beyond the scope of the present paper, we would use word shift graphs [14, 44, 46, 63, 73] to illuminate which 1-grams drive changes in ousiometric scores.

We note that these instruments are not inherently predictive for social phenomena, but rather extract real-time signals of essential meaning from online text. Analysis of such signals in pursuit of prediction is itself a separate, massive, and fraught enterprise [77–79].

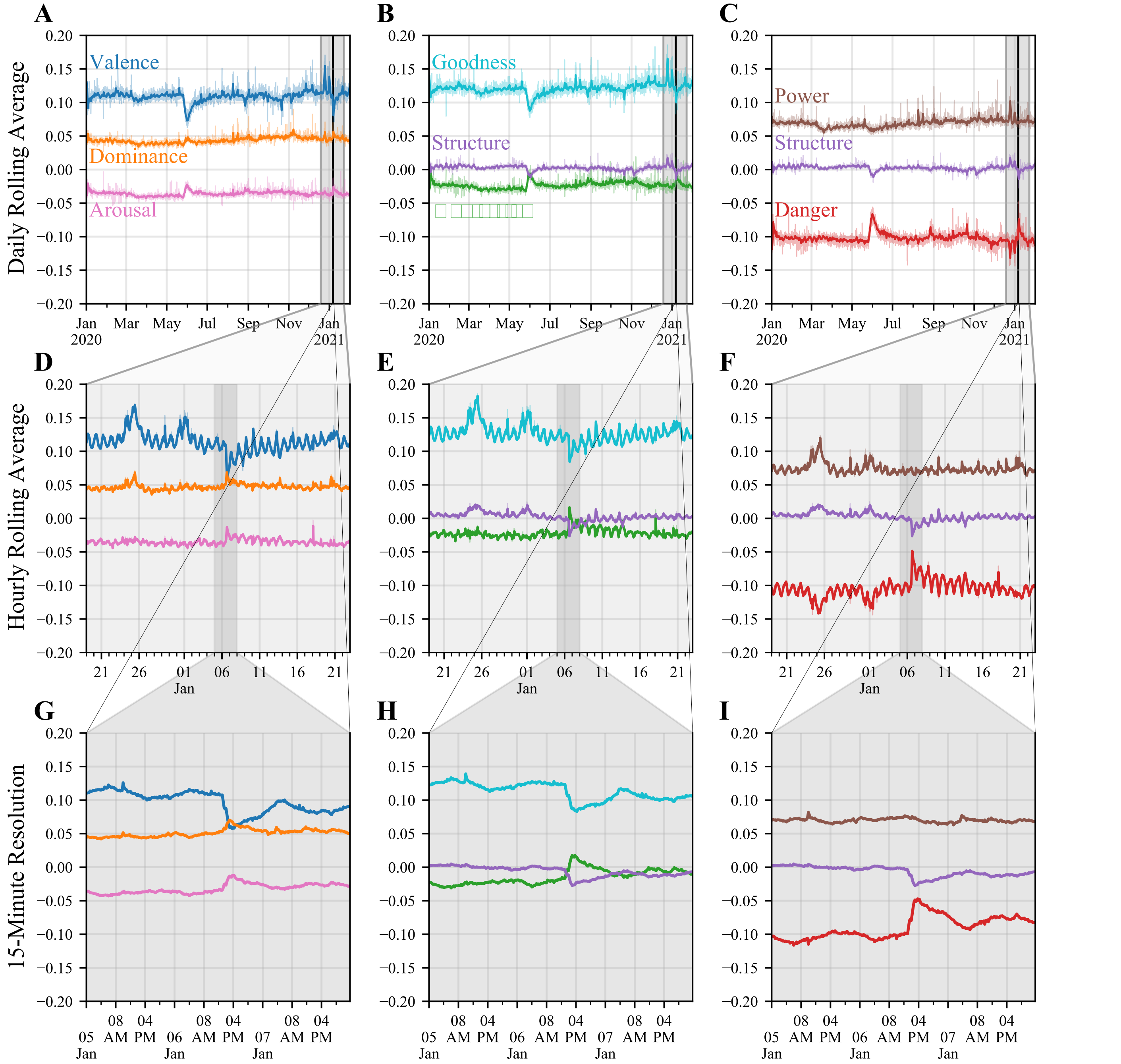

Figure S74: The ousiometer: Example essential meaning time series for Twitter, 2020/01–2021/01. The three columns correspond to average meaning scores for the frameworks of VAD, GAS, and PDS, computed per Eq. (5). The first row shows time series for the 13 months covering all of 2020 and January, 2021. The second and third rows focus in on the attack on the US Capitol on 2021/01/06 by supporters of President Trump. The scale for the second row is 5 weeks (2020/12/19 to 2021/01/23) and 3 days (2021/01/05 to 2021/01/07) for the third row. All underlying time series are 15 minute time scales with day-scale and hour-scale smoothing overlaid in the first and second rows. Major events with spikes and/or durable memory are the US's assassination of the Iranian general Soleimani, the COVID pandemic, George Floyd's murder, and events related to the 2020 US presidential election, including the attack on the US Capitol. Because dominance is relatively stable throughout, the GAS and PDS dimensions effectively vary as functions only of valence and arousal (see Eqs. 2 and 4). In particular, goodness and aggression track valence and arousal closely. For the 2021/01/06 attack, the danger time series spikes while power remains stable (panels F and I). Structure drops indicating increased seriousness. In total, even though only three are independent, power, danger, goodness, aggression, and structure are all valuable time series. Notes: We constructed the Twitter 1-gram corpus from approximately 10% of all English tweets [57, 65], with all 1-grams moved to lower case. We form a lexical lens $\mathcal{L}$ by taking 1-grams from the NRC VAD lexicon and adding a hashtag version of each 1-gram. As such, the ousiometer is not specifically tailored for Twitter during the time period covered. As we have done for the hedonometer [14, 80], our ousiometer could be readily improved by expanding the lexical lens to incorporate missing salient 1-grams.

REFERENCES

1. G. Lakoff, M. Johnson, *Metaphors We Live By* (University of Chicago Press, 1980).

2. G. A. Miller, WordNet: A lexical database for English. *Commun. ACM* **38**, 39–41 (1995).

3. T. Mikolov, I. Sutskever, K. Chen, G. S. Corrado, J. Dean, Distributed representations of words and phrases and their compositionality. In *Advances in Neural Information Processing Systems 26* (Curran Associates, Inc., Red Hook, NY, NIPS2013), pp. 3111–3119.

4. C. E. Osgood, The nature and measurement of meaning. *Psychol. Bull.* **49**, 197–237 (1952).

5. L. M. Solomon, "A factorial study of complex auditory stimuli (passive sonar sounds)," thesis, University of Illinois (1954).

6. C. Osgood, G. Suci, P. Tannenbaum, *The Measurement of Meaning* (University of Illinois, 1957).

7. C. E. Osgood, On the whys and wherefores of E, P, and A. *J. Pers. Soc. Psychol.* **12**, 194–199 (1969).

8. C. E. Osgood, W. H. May, M. S. Miron, M. S. Miron, *Cross-Cultural Universals of Affective Meaning* (University of Illinois Press, 1975).

9. J. A. Russell, A circumplex model of affect. *J. Pers. Soc. Psychol.* **39**, 1161–1178 (1980).

10. J. A. Russell, Core affect and the psychological construction of emotion. *Psychol. Rev.* **110**, 145–172 (2003).

11. A. Mehrabian, J. A. Russell, *An Approach to Environmental Psychology* (MIT Press, 1974).

12. A. Mehrabian, J. A. Russell, The basic emotional impact of environments. *Percept. Mot. Skills* **38**, 283–301 (1974).

13. M. M. Bradley, P. J. Lang, Affective norms for English words (ANEW): Stimuli, instruction manual and affective ratings. Technical report c-1, University of Florida, Gainesville, FL (1999).

14. P. S. Dodds, K. D. Harris, I. M. Kloumann, C. A. Bliss, C. M. Danforth, Temporal patterns of happiness and information in a global social network: Hedonometrics and Twitter. *PLOS ONE* **6**, e26752 (2011).

15. A. B. Warriner, V. Kuperman, M. Brysbaert, Norms of valence, arousal, and dominance for 13,915 English lemmas. *Behav. Res. Methods* **45**, 1191–1207 (2013).

16. A. B. Warriner, V. Kuperman, Affective biases in English are bi-dimensional. *Cogn. Emot.* **29**, 1147–1167 (2015).

17. A. B. Warriner, D. I. Shore, L. A. Schmidt, C. L. Imbault, V. Kuperman, Sliding into happiness: A new tool for measuring affective responses to words. *Can. J. Exp. Psychol.* **71**, 71–88 (2017).

18. S. M. Mohammad, "Word affect intensities," in *Proceedings of the 11th Edition of the Language Resources and Evaluation Conference (LREC-2018)* (European Language Resources Association, 2018), pp. 1–10.

19. S. M. Mohammad, "Obtaining reliable human ratings of valence, arousal, and dominance for 20,000 English words," in *Proceedings of The Annual Conference of the Association for Computational Linguistics (ACL)* (Association for Computational Linguistics, 2018), pp. 174–184.

20. T. F. Karwoski, H. S. Odbert, Color-music. *Psychol. Monogr.* **50**, i–60 (1938).

21. G. Grand, I. A. Blank, F. Pereira, E. Fedorenko, Semantic projection recovers rich human knowledge of multiple object features from word embeddings. *Nat. Hum. Behav.* **6**, 975–987 (2022).

22. L. L. Thurstone, Multiple factor analysis. *Psychol. Rev.* **38**, 406–427 (1931).

23. K. G. Jöreskog, A general approach to confirmatory maximum likelihood factor analysis. *Psychometrika* **34**, 183–202 (1969).

24. L. R. Fabrigar, D. T. Wegener, R. C. MacCallum, E. J. Strahan, Evaluating the use of exploratory factor analysis in psychological research. *Psychol. Methods* **4**, 272–299 (1999).

25. L. L. Visinescu, N. Evangelopoulos, Orthogonal rotations in latent semantic analysis: An empirical study. *Decis. Support Syst.* **62**, 131–143 (2014).

26. R. I. Jennrich, A simple general method for oblique rotation. *Psychometrika* **67**, 7–19 (2002).

27. L. F. Barrett, J. A. Russell, The structure of current affect: Controversies and emerging consensus. *Curr. Dir. Psychol. Sci.* **8**, 10–14 (1999).

28. M. Yik, J. A. Russell, J. H. Steiger, A 12-point circumplex structure of core affect. *Emotion* **11**, 705–731 (2011).

29. W. M. Wundt, *Grundriss der Psychologie* (Kröner, 1922).

30. R. Reisenzein, Wundt's three-dimensional theory of emotion. *Poznan Stud. Philos. Sci. Hum.* **75**, 219–250 (2000).

31. M. M. Bradley, P. J. Lang, Measuring emotion: The self-assessment manikin and the semantic differential. *J. Behav. Ther. Exp. Psychiatry* **25**, 49–59 (1994).

32. S. Gannouni, A. Aledaily, K. Belwafi, H. Aboalsamh, Adaptive emotion detection using the valence-arousal-dominance model and eeg brain rhythmic activity changes in relevant brain lobes. *IEEE Access* **8**, 67444–67455 (2020).

33. P. J. Lang, M. M. Bradley, B. N. Cuthbert, International Affective Picture System (IAPS): Technical manual and affective ratings. Technical report, NIMH Center for the Study of Emotion and Attention (1997).

34. M. El-Haj, R. Takanami, Unifying emotion analysis datasets using Valence Arousal Dominance (VAD), in *Proceedings of the 4th Conference on Language, Data and Knowledge,* Vienna, Austria, 2023. NOVA CLUNL, Portugal.

35. S. M. Mohammad, "Obtaining reliable human ratings of valence, arousal, and dominance for 20,000 English words," in *Proceedings of the 56th Annual Meeting of the Association for Computational Linguistics* (Association for Computational Linguistics, 2018), pp. 174–184.

36. M. Mäntylä, B. Adams, G. Destefanis, D. Graziotin, M. Ortu, Mining valence, arousal, anddominance: Possibilities for detecting burnout andproductivity? in *Proceedings of the 13thInternational Conference on Mining Software Repositories, ICSE '16* (ACM, 2016), pp. 247–258.

37. S. M. Mohammad, NRC VAD Lexicon v2: Norms for valence, arousal, and dominance for over 55k English terms. arXiv:2503.23547 [cs.CL] (2025).

38. K. Yang, T. Zhang, S. Ananiadou, , Disentangled variational autoencoder for emotion recognition in conversations. *IEEE Trans. Affect. Comput.* **15**, 508–518 (2024).

39. S. Park, J. Kim, S. Ye, J. Jeon, H. Y. Park, A. Oh, Dimensional emotion detection from categorical emotion, in *Proceedings of the 2021 Conference on Empirical Methods in Natural Language Processing* (Association for Computational Linguistics, 2021), pp. 4367–4380.

40. P. J. Lang, M. K. Greenwald, M. M. Bradley, A. O. Hamm, Looking at pictures: Affective, facial, visceral, and behavioral reactions. *Psychophysiology* **30**, 261–273 (1993).

41. G. K. Verma, U. S. Tiwary, Affect representation and recognition in 3d continuous valence–arousal–dominance space. *Multimed. Tools Appl.* **76**, 2159–2183 (2017).

42. M. Li, Application of sentence-level text analysis: The role of emotion in an experimental learning intervention. *J. Exp. Soc. Psychol.* **99**, 104278 (2017).

43. Z. Guendil, Z. Lachiri, C. Maaoui, Computational framework for emotional VAD prediction using regularized Extreme Learning Machine. *Int. J. Multimed. Inf. Retr.* **6**, 251–261 (2017).

44. I. Bakker, T. Van Der Voordt, P. Vink, J. De Boon, Pleasure, arousal, dominance: Mehrabian and Russell revisited. *Curr. Psychol.* **33**, 405–421 (2014).

45. P. S. Dodds, E. M. Clark, S. Desu, M. R. Frank, A. J. Reagan, J. R. Williams, L. Mitchell, K. D. Harris, I. M. Kloumann, J. P. Bagrow, K. Megerdoomian, M. T. McMahon, B. F. Tivnan, C. M. Danforth, Human language reveals a universal positivity bias. *Proc. Natl. Acad. Sci. U.S.A.* **112**, 2389–2394 (2015).

46. G. Herdan, *Type-Token Mathematics: A Textbook of Mathematical Linguistics* (Mouton, 1960).

47. A. J. Reagan, C. M. Danforth, B. F. Tivnan, J. R. Williams, P. S. Dodds, Sentiment analysis methods for understanding large-scale texts: A case for using continuum-scored words and word shift graphs. *EPJ Data Sci.* **6**, 28 (2017).

48. J. Boucher, C. E. Osgood, The Pollyanna hypothesis. *J. Verbal Learn. Verbal Behav.* **8**, 1–8 (1969).

49. J. J. Louviere, T. N. Flynn, A. A. J. Marley, *Best-Worst Scaling: Theory, Methods and Applications* (Cambridge Univ. Press, 2015).

50. P. S. Dodds, J. W. Zimmerman, C. G. Beauregard, A. M. A. Fehr, M. I. Fudolig, T. R. Tangherlini, C. M. Danforth, Archetypometrics, a Pragmateia: Empirical determination of the fundamental archetypes of fictional characters. Working paper, Zenodo (2025); https://doi.org/10.5281/zenodo.17117974.

51. G. K. Zipf, *Human Behaviour and the Principle of Least-Effort* (Addison-Wesley, 1949).

52. J.-B. Michel, Y. K. Shen, A. P. Aiden, A. Veres, M. K. Gray, The Google Books Team, J. P. Pickett, D. Hoiberg, D. Clancy, P. Norvig, J. Orwant, S. Pinker, M. A. Nowak, E. A. Lieberman, Quantitative analysis of culture using millions of digitized books. *Science* **331**, 176–182 (2011).

53. E. A. Pechenick, C. M. Danforth, P. S. Dodds, Characterizing the Google Books corpus: Strong limits to inferences of socio-cultural and linguistic evolution. *PLOS ONE* **10**, e0137041 (2015).

54. E. Sandhaus, *The New York Times Annotated Corpus* (Linguistic Data Consortium, 2008); https://doi.org/10.35111/77ba-9x74.

55. I. Semenov, Wikipedia word frequency (2019); https://github.com/IlyaSemenov/wikipedia-word-frequency [accessed 2021 April 2].

56. D. Beeferman, W. Brannon, D. Roy, RadioTalk: A large-scale corpus of talk radio transcripts. arXiv:1907.07073 [cs.CL] (2019).

57. T. Alshaabi, J. L. Adams, M. V. Arnold, J. R. Minot, D. R. Dewhurst, A. J. Reagan, C. M. Danforth, P. S. Dodds, Storywrangler: A massive exploratorium for sociolinguistic, cultural, socioeconomic, and political timelines using Twitter. *Sci. Adv.* **7**, eabe6534 (2021).

58. H. A. Simon, On a class of skew distribution functions. *Biometrika* **42**, 425–440 (1955).

59. J. R. Williams, P. R. Lessard, S. Desu, E. M. Clark, J. P. Bagrow, C. M. Danforth, P. S. Dodds, Zipf's law holds for phrases, not words. *Nat. Sci. Rep.* **5**, 12209 (2015).

60. M. E. J. Newman, Power laws, Pareto distributions and Zipf's law. *Contemp. Phys.* **46**, 323–351 (2005).

61. P. S. Dodds, J. R. Minot, M. V. Arnold, T. Alshaabi, J. L. Adams, D. R. Dewhurst, T. J. Gray, M. R. Frank, A. J. Reagan, C. M. Danforth, Allotaxonometry and rank-turbulence divergence: A universal instrument for comparing complex systems. *EPJ Data Sci.* **12**, 37 (2023).

62. G. Strang, *Introduction to Linear Algebra* (Cambridge Wellesley Press, ed. 4, 2009).

63. P. S. Dodds, C. M. Danforth, Measuring the happiness of large-scale written expression: Songs, blogs, and presidents. *J. Happiness Stud.* **11**, 441–456 (2009).

64. I. M. Kloumann, C. M. Danforth, K. D. Harris, C. A. Bliss, P. S. Dodds, Positivity of the English language. *PLOS ONE* **7**, e29484 (2012).

65. H. Schlosberg, The description of facial expressions in terms of two dimensions. *J. Exp. Psychol.* **44**, 229–237 (1952).

66. H. Schlosberg, Three dimensions of emotion. *Psychol. Rev.* **61**, 81–88 (1954).

67. J. A. Russell, “How shall an emotion be called?,” in *Circumplex Models of Personality and Emotions*, R. Plutchik, H. R. Conte, Eds. (American Psychological Association, 1997), pp. 205–220.

68. L. Feldman Barrett, J. A. Russell, Independence and bipolarity in the structure of current affect. *J. Pers. Soc. Psychol.* **74**, 967–984 (1998).

69. S. Fillenbaum, *Structures in the Subjective Lexicon* (Academic Press, 1971).

70. P. S. Dodds, Archetypometrics dataset. Zenodo dataset (2025); https://doi.org/10.5281/zenodo.16953724.

71. F. Y. Edgeworth, *Mathematical Psychics: An Essay into the Application of Mathematics to Moral Sciences* (Kegan Paul, 1881).

72. A. J. Reagan, L. Mitchell, C. M. Danforth, P. S. Dodds, The emotional arcs of stories are dominated by six basic shapes. *EPJ Data Sci.* **5**, 31 (2016).

73. R. J. Gallagher, M. R. Frank, L. Mitchell, A. J. Schwartz, A. J. Reagan, C. M. Danforth, P. S. Dodds, Generalized word shift graphs: A method for visualizing and explaining pairwise comparisons between texts. *EPJ Data Sci.* **10**, 4 (2021).

74. Kurt Vonnegut on the shapes of stories (2010); https://youtube.com/watch?v=oP3c1h8v2ZQ [accessed 15 May 2014].

75. M. I. Fudolig, T. Alshaabi, K. Cramer, C. M. Danforth, P. S. Dodds, A decomposition of book structure through ousiometric fluctuations in cumulative word-time. *Humanit. Soc. Sci. Commun.* **10**, 187 (2023).

76. G. Miller, Social scientists wade into the tweet stream. *Science* **333**, 1814–1815 (2011).

77. A. Bermingham, A. Smeaton, “On using Twitter to monitor political sentiment and predict election results,” in *Proceedings of the Workshop on Sentiment Analysis where AI meets Psychology (SAAIP 2011)* (Asian Federation of Natural Language Processing, 2011), pp. 2–10.

78. D. Gayo-Avello, I wanted to predict elections with Twitter and all I got was this lousy paper: A balanced survey on election prediction using Twitter data. *Commun. ACM* **55**, 89–96 (2012).

79. A. Jungherr, P. Jürgens, Forecasting the pulse: How deviations from regular patterns in online data can identify offline phenomena. *Internet Res.* **23**, 589–607 (2013).

80. T. Alshaabi, M. V. Arnold, J. R. Minot, J. L. Adams, D. R. Dewhurst, A. J. Reagan, R. Muhamad, C. M. Danforth, P. S. Dodds, How the world's collective attention is being paid to a pandemic: COVID-19 related *n*-gram time series for 24 languages on Twitter. *PLOS ONE* **16**, e0244476 (2021).

81. P. S. Dodds, J. R. Minot, M. V. Arnold, T. Alshaabi, J. L. Adams, A. J. Reagan, C. M. Danforth, Computational timeline reconstruction of the stories surrounding Trump: Story turbulence, narrative control, and collective chronopathy. *PLOS ONE* **16**, e0260592, 2021.

82. T. Alshaabi, D. R. Dewhurst, J. R. Minot, M. V. Arnold, J. L. Adams, C. M. Danforth, P. S. Dodds, The growing amplification of social media: Measuring temporal and social contagion dynamics for over 150 languages on Twitter for 2009–2020. *EPJ Data Sci.* **10**, 15 (2021).

83. P. S. Dodds. Essential meaning word scores for ousiometric framework of goodness-power-aggression-danger-structure (GPADS). Zenodo dataset (2026); https://doi.org/10.5281/zenodo.15784610.
